# Supplementary material for: Unequal access? Use of sickness absence benefits by precariously employed workers with common mental disorders: a register-based cohort study in Sweden
Source: BMJ Open. 2023 Jul 19;13(7):e072459. doi: 10.1136/bmjopen-2023-072459 (PMC10357787; doi:10.1136/bmjopen-2023-072459)
Supplement: Supplementary data [file bmjopen-2023-072459supp001.pdf]

Supplementary Table 1. Sociodemographic and employment-related characteristics at baseline (2016) of the Swedish working population and the study population with common mental disorders.

	**Population***	**Study population****
	N (%)	N (%)
**Age**		
27-40 years	1,044,971 (37.4)	30,675 (39.2)
41-51 years	976,697 (34.9)	28,541 (36.5)
52-61 years	774,838 (27.7)	18,999 (24.3)
**Gender**		
Male	1,412,310 (50.5)	25,894 (33.1)
Female	1,384,196 (49.5)	52,321 (66.9)
**Education level**		
Elementary/high school education	1,518,999 (54.4)	41,072 (52.5)
Higher education <3 years	429,648 (15.4)	11,456 (14.7)
Higher education ≥3 years	845,355 (30.3)	25,651 (32.8)
**Country of birth**		
Sweden	2,459,096 (88.0)	69,736 (89.2)
Not Sweden	335,518 (12.0)	8,438 (10.8)
**Family composition**		
Couple with children	1,400,506 (50.1)	37,471 (47.9)
Couple without children	352,101 (12.6)	8,421 (10.8)
Single with children	260,276 (9.3)	10,243 (13.1)
Single without children	783,622 (28.0)	22,080 (28.2)
**Unemployment**		
0 days unemployed	2,718,662 (97.2)	76,090 (97.3)
From 1 to 90 days unemployed	77,844 (2.8)	2,125 (2.7)
**Economic sector**		
Private	1,741,419 (62.3)	41,653 (53.3)
Public	1,055,087 (37.7)	36,562 (46.7)
TOTAL	2,796,506 (100.0)	78,215 (100.0)

*The entire study population meeting the inclusion and exclusion criteria (except having Selective Serotonin Reuptake Inhibitors prescriptions in 2017); **The final study population meeting the whole inclusion and exclusion criteria. Missing values in educational level (population: 2,504, 0.09%; study population: 41, 0.05%), country of birth (population: 1892, 0.07%; study population: 41, 0.05%) and family composition (population: 1, <0.01%).

Hernando-Rodriguez JC, *et al. BMJ Open* 2023; 13:e072459. doi: 10.1136/bmjopen-2023-072459

Supplementary Table 2. Incidence of a first sickness absence episode due to common mental disorders in a cohort of 78,215 individuals with common mental disorders in 2017, aged 27-61, entitled to sickness absence benefits in 2016, by sociodemographic and employment characteristics at baseline (2016)

	**Sickness absence benefits (7.48%)**
	N (%)
**Age**	
27-40 years	2,808 (9.2)
41-51 years	1,901 (6.7)
52-61 years	1,139 (6.0)
**Gender**	
Male	2,136 (8.2)
Female	3,712 (7.1)
**Education level**	
Elementary/high school education	3,502 (8.5)
Higher education <3 years	793 (6.9)
Higher education ≥3 years	1,547 (6.0)
**Country of birth**	
Sweden	5,058 (7.3)
Not Sweden	783 (9.3)
**Family composition**	
Couple with children	2,678 (7.1)
Couple with no children	519 (6.2)
Single with children	808 (7.9)
Single	1,843 (8.3)
**Unemployment**	
0 days unemployed	5,697 (7.5)
From 1 to 90 days unemployed	151 (7.1)
**Economic sector**	
Private	3218 (7.7)
Public	2630 (7.2)
**Employment quality typology**	
Standard Employment Relationship/HI	593 (5.2)
Standard Employment Relationship	2,268 (7.6)
Sub-standard Employment Relationship	2,667 (8.1)
Precarious Employment Relationship	320 (8.0)
**Contractual relationship insecurity**	
Directly employed by an employer	5,788 (7.5)
Employed by an agency	60 (8.2)
**Contractual temporariness**	
Stable employment	4,351 (7.4)
Unstable employment	1,497 (7.8)
**Multiple jobs/economic sectors**	
1-2 jobs	5,436 (7.4)
3 or more jobs	279 (7.8)
3 or more jobs in 3 or more sectors	133 (8.2)
**Income level***	
<60% of the median	395 (7.6)
60-79% of the median	1,415 (8.9)
80-119% of the median	3,113 (7.7)
120-200% of the median	831 (5.7)
>200% of the median	94 (4.0)
**Unionization level**	
<70 %	124 (7.0)
70-90%	315 (6.8)
>90%	5,409 (7.5)

Missing values in the educational level (36, 0.05%) and country of birth (41, 0.05%). Note: Persons entitled to sickness absence benefits in 2016 are those having an annual income from paid employment of at least 24% of the price base amount of 2016; that is, 10,632 SEK. *Income level categories based on the median of the Swedish working population (375,300 SEK) after applying several inclusion and exclusion criteria (see methods section). Income category boundaries: <60% of the median (225,180 SEK), 60-79% of the median (225,180-296,487 SEK), 80-119% of the median (300,240-446,607 SEK), 120-199% of the median (450,360-746,847 SEK), and ≥200% of the median (750,600 SEK).

Supplementary Table 3. Incidence of a first sickness absence episode due to common mental disorders in 2017 in women and men in a cohort of 85,164 individuals with common mental disorders, aged 27-61, entitled to sickness absence benefits in 2016, by sociodemographic and employment characteristics at baseline (2016).

	Sickness absence benefits	
	**Women (7.1%)**	**Men (8.2%)**
	N (%)	N (%)
**Age**		
27-40 years	1782 (8.7)	1026 (10.2)
41-51 years	1204 (6.3)	697 (7.4)
52-61 years	726 (5.8)	413 (6.4)
**Education level**		
Elementary/high school education	2016 (8.0)	1486 (9.3)
Higher education <3 years	484 (6.3)	309 (8.2)
Higher education ≥3 years	1208 (6.2)	339 (5.5)
**Country of birth**		
Sweden	3195 (6.9)	1863 (8.0)
Not Sweden	512 (8.8)	271 (10.4)
**Family composition**		
Couple with children	1735 (6.8)	943 (7.8)
Couple without children	357 (6.2)	162 (6.2)
Single with children	647 (7.6)	161 (9.2)
Single without children	973 (7.7)	870 (9.2)
**Unemployment**		
0 days unemployed	3632 (7.1)	2065 (8.2)
From 1 to 90 days unemployed	80 (6.0)	71 (8.9)
**Economic sector**		
Private	1,594 (7.0)	1,624 (8.5)
Public	2,118 (7.1)	512 (7.4)
**Employment quality typology**		
Standard Employment Relationship/HI	247 (4.5)	346 (5.8)
Standard Employment Relationship	1375 (7.0)	893 (8.8)
Sub-standard Employment Relationship	1892 (7.7)	775 (9.1)
Precarious Employment Relationship	198 (7.1)	122 (10.1)
**Contractual relationship insecurity**		
Directly employed by an employer	3683 (7.1)	2105 (8.2)
Employed by an agency	29 (6.5)	31 (10.9)
**Contractual temporariness**		
Stable employment	2792 (7.0)	1559 (8.0)
Unstable employment	920 (7.2)	577 (9.0)
**Multiple jobs/economic sectors**		
1-2 jobs	3463 (7.1)	1973 (8.2)
3 or more jobs	175 (7.0)	104 (9.6)
3 or more jobs in 3 or more sectors	74 (7.3)	59 (9.6)
**Income level**		
<60% of the median	319 (7.3)	76 (8.9)
60-79% of the median	1111 (8.4)	304 (10.9)
80-119% of the median	1876 (7.1)	1237 (8.9)
120-200% of the median	378 (5.1)	453 (6.4)
>200% of the median	28 (2.9)	66 (4.9)
**Unionization level**		
<70 %	54 (5.8)	70 (8.2)
70-90%	161 (6.1)	154 (7.7)
>90%	3497 (7.2)	1912 (8.3)

Missing values in education level (women: 19, 0.04%; men: 17, 0.07%) and country of birth (women: 19, 0.04%; men: 22, 0.08%). Note: Persons entitled to sickness absence benefits in 2016 are those having an annual income from paid employment of at least 24% of the price base amount of 2016; that is, 10,632 SEK. *Income level categories based on the median of the Swedish working population (375,300 SEK) after applying several inclusion and exclusion criteria (see methods section). Income category boundaries: <60% of the median (225,180 SEK), 60-79% of the median (225,180-296,487 SEK), 80-119% of the median (300,240-446,607 SEK), 120-199% of the median (450,360-746,847 SEK), and ≥200% of the median (750,600 SEK).

Supplementary Table 4. Incidence of a first sickness absence episode due to common mental disorders in a cohort of 85,164 individuals with common mental disorders in 2017, aged 27-61, entitled to sickness absence benefits in 2016, by employment quality typology (Standard Employment Relationship, SER; Standard Employment Relationship with high income, SER/HI; Sub-standard Employment Relationship, SSER; Precarious Employment Relationship, PER; baseline, 2016).

	Sickness absence benefits			
	**SER/HI (5.2%)**	**SER (7.6%)**	**SSER (8.1%)**	**PER (8%)**
	N (%)	N (%)	N (%)	N (%)
**Age**				
27-40 years	180 (6.3)	938 (9.6)	1481 (9.5)	209 (8.8)
41-51 years	253 (4.8)	794 (6.9)	773 (7.1)	81 (7.9)
52-61 years	160 (4.8)	536 (6.3)	413 (6.3)	30 (5.1)
**Gender**				
Male	346 (5.8)	893 (8.8)	775 (9.1)	122 (10.1)
Female	247 (4.5)	1375 (7.0)	1892 (7.7)	198 (7.1)
**Education level**				
Elementary/high school education	209 (6.2)	1340 (8.5)	1731 (9.0)	222 (8.8)
Higher education <3 years	116 (5.8)	321 (7.3)	311 (7.0)	45 (7.1)
Higher education ≥3 years	268 (4.4)	606 (6.4)	620 (6.7)	53 (6.4)
**Country of birth**				
Sweden	527 (5.0)	1962 (7.4)	2282 (7.8)	287 (8.1)
Not Sweden	66 (6.4)	305 (9.6)	379 (10.1)	33 (7.1)
**Family composition**				
Couple with children	288 (4.8)	971 (7.3)	1286 (7.9)	133 (7.5)
Couple without children	65 (4.8)	241 (6.6)	202 (6.4)	11 (3.8)
Single with children	59 (4.7)	304 (7.8)	396 (8.7)	49 (8.8)
Single without children	181 (6.4)	752 (8.4)	783 (8.7)	127 (9.3)
**Unemployment**				
0 days unemployed	590 (5.2)	2253 (7.6)	2589 (8.2)	265 (7.8)
From 1 to 90 days unemployed	3 (5.6)	15 (6.3)	78 (6.2)	55 (9.6)
**Economic sector**				
Private	384 (5.5)	1,186 (8.5)	1,383 (7.9)	265 (8.3)
Public	209 (4.7)	1,082 (6.8)	1,284 (8.3)	55 (6.8)
**Contractual relationship insecurity**				
Directly employed by an employer	593 (5.2)	2263 (7.6)	2636 (8.1)	296 (7.9)
Employed by an agency	0	5 (13.2)	31 (6.7)	24 (10.9)
**Contractual temporariness**				
Stable employment	593 (5.2)	2246 (7.7)	1503 (8.3)	9 (3.5)
Unstable employment	0	22 (4.7)	1164 (7.8)	311 (8.3)
**Multiple jobs/economic sectors**				
1-2 jobs	579 (5.2)	2220 (7.6)	2479 (8.1)	158 (7.3)
3 or more jobs	11 (5.0)	36 (8.2)	155 (8.1)	77 (7.7)
3 or more jobs in 3 or more sectors	3 (2.5)	12 (6.3)	33 (6.7)	85 (10.3)
**Income level**				
<60% of the median	0	0	270 (8.1)	125 (6.6)
60-79% of the median	0	0	1313 (8.9)	102 (8.8)
80-119% of the median	0	2203 (7.7)	822 (7.7)	88 (10.0)
120-200% of the median	536 (5.4)	42 (7.1)	249 (6.3)	4 (6.5)
>200% of the median	57 (3.5)	23 (4.5)	13 (7.1)	1 (100.0)
**Unionization level**				
<70%	0	1 (5.0)	58 (6.8)	65 (7.2)
70-90%	2 (3.6)	26 (6.3)	223 (6.8)	64 (7.4)
>90%	591 (5.2)	2241 (7.6)	2386 (8.3)	191 (8.6)

Missing values in education level (SER/HI: 7, 0.06%; SER: 10, 0.03%; SSER: 17, 0.05%; PE: 2, 0.05%) and country of birth (SER/HI: 3, 0.03%; SER: 18, 0.06%; SSER: 20, 0.06%). Note: Persons entitled to sickness absence benefits in 2016 are those having an annual income from paid employment of at least 24% of the price base amount of 2016; that is, 10,632 SEK. *Income level categories based on the median of the Swedish working population (375,300 SEK) after applying several inclusion and exclusion criteria (see methods section). Income category boundaries: <60% of the median (225,180 SEK), 60-79% of the median (225,180-296,487 SEK), 80-119% of the median (300,240-446,607 SEK), 120-199% of the median (450,360-746,847 SEK), and ≥200% of the median (750,600 SEK).

Supplementary Table 5. Association between employment quality dimensions (2016, baseline) and sickness absence benefits due to CMD in 2017 (N=78,215) among people entitled to sickness absence benefits in 2016.

					**Women (N=52,321)**				**Men (N=25,894)**			
	**Cases N(%)**	**OR (95% CI)**	**aOR (95% CI) ¹**	**aOR (95% CI) ²**	**Cases N(%)**	**OR (95% CI)**	**aOR (95% CI) ¹**	**aOR (95% CI) ²**	**Cases N(%)**	**OR (95% CI)**	**aOR (95% CI) ¹**	**aOR (95% CI) ²**
**Contractual relationship insecurity**												
Directly employed by an employer	5,788 (7.5)	1	1	1	3,683 (7.1)	1	1	1	2,105 (8.2)	1	1	1
Employed by an agency	60 (8.2)	1.10 (0.85-1.44)	1.05 (0.80-1.37)	0.96 (0.73-1.25)	29 (6.5)	0.91 (0.62-1.32)	0.89 (0.61-1.31)	0.85 (0.58-1.24)	31 (10.9)	1.36 (0.94-1.98)	1.19 (0.81-1.73)	1.08 (0.74-1.58)
**Contractual temporariness**												
Stable employment	4,351 (7.4)	1	1	1	2,792 (7.1)	1	1	1	1,559 (8.0)	1	1	1
Unstable employment	1,497 (7.8)	1.07 (1.01-1.14)	1.08 (1.02-1.15)	1.02 (0.95-1.09)	920 (7.2)	1.03 (0.95-1.11)	1.06 (0.98-1.15)	1.01 (0.92-1.10)	577 (9.0)	1.14 (1.03-1.26)	1.12 (1.01-1.24)	1.04 (0.93-1.16)
**Multiple jobs/economic sectors**												
1-2 jobs	5,436 (7.4)	1	1	1	3,463 (7.1)	1	1	1	1,973 (8.2)	1	1	1
3 or more jobs	279 (7.8)	1.05 (0.93-1.19)	1.06 (0.93-1.20)	1.05 (0.93-1.20)	175 (7.0)	0.99 (0.85-1.16)	1.02 (0.87-1.20)	1.00 (0.85-1.18)	104 (9.6)	1.19 (0.97-1.47)	1.17 (0.95-1.45)	1.18 (0.95-1.46)
3 or more jobs in 3 or more sectors	133 (8.2)	1.11 (0.93-1.33)	1.11 (0.93-1.34)	1.09 (0.91-1.31)	74 (7.3)	1.04 (0.82-1.32)	1.07 (0.84-1.36)	1.04 (0.81-1.32)	59 (9.6)	1.19 (0.91-1.56)	1.16 (0.88-1.53)	1.19 (0.90-1.57)
**Income level**												
80-119% of the median	3,113 (7.7)	1	1	1	1,876 (7.1)	1	1	1	1,237 (8.9)	1	1	1
<60% of the median	395 (7.6)	0.98 (0.88-1.09)	0.98 (0.88-1.10)	0.92 (0.82-1.03)	319 (7.3)	1.03 (0.91-1.17)	1.05 (0.93-1.19)	0.91 (0.81-1.04)	76 (8.9)	1.00 (0.78-1.27)	0.99 (0.77-1.26)	0.94 (0.73-1.20)
60-79% of the median	1,415 (8.9)	1.16 (1.09-1.24)	1.17 (1.09-1.24)	1.10 (1.03-1.18)	1,111 (8.4)	1.20 (1.11-1.30)	1.21 (1.12-1.31)	1.08 (1.00-1.17)	304 (10.9)	1.24 (1.09-1.42)	1.25 (1.10-1.43)	1.16 (1.01-1.33)
120-200% of the median	831 (5.8)	0.73 (0.67-0.79)	0.72 (0.67-0.78)	0.79 (0.72-0.85)	378 (5.1)	0.70 (0.63-0.79)	0.70 (0.63-0.79)	0.79 (0.70-0.88)	453 (6.4)	0.70 (0.62-0.78)	0.69 (0.62-0.78)	0.80 (0.71-0.89)
>200% of the median	94 (4.1)	0.50 (0.41-0.62)	0.50 (0.40-0.62)	0.61 (0.49-0.75)	28 (2.9)	0.39 (0.26-0.57)	0.39 (0.26-0.56)	0.48 (0.33-0.71)	66 (4.9)	0.52 (0.41-0.68)	0.51 (0.40-0.66)	0.71 (0.54-0.92)
**Unionization level**												
>90%	5,409 (7.5)	1	1	1	3,497 (7.2)	1	1	1	1,912 (8.3)	1	1	1
70-90%	315 (6.8)	0.90 (0.80-1.01)	0.86 (0.77-0.97)	0.82 (0.73-0.92)	161 (6.1)	0.84 (0.72-0.99)	0.82 (0.70-0.97)	0.83 (0.70-0.98)	154 (7.7)	0.92 (0.78-1.10)	0.84 (0.71-1.00)	0.80 (0.67-0.96)
<70%	124 (7.0)	0.92 (0.77-1.11)	0.87 (0.72-1.05)	0.82 (0.68-0.99)	54 (5.8)	0.80 (0.61-1.06)	0.76 (0.58-1.01)	0.77 (0.58-1.03)	70 (8.2)	0.99 (0.77-1.27)	0.87 (0.67-1.12)	0.84 (0.65-1.09)

OR: unadjusted Odds Ratio; ¹ aOR: Odds Ratio adjusted mutually by the other employment quality dimensions in the baseline (2016); ² aOR: Odds Ratio adjusted mutually by the other employment quality dimensions and age, gender, education, country of birth, family composition, economic sector, unemployment, and SA due to diagnoses other than CMD in the baseline (2016); Note: Persons entitled to sickness absence benefits are those having an annual income from paid employment of at least 24% of the price base amount of 2016; that is, 10,632 SEK. *Income level categories based on the median of the Swedish working population after applying the inclusion and some of the main exclusion criteria (see methods section). *Income level categories based on the median of the Swedish working population (375,300 SEK) after applying several inclusion and exclusion criteria (see methods section). Income category boundaries: <60% of the median (225,180 SEK), 60-79% of the median (225,180-296,487 SEK), 80-119% of the median (300,240-446,607 SEK), 120-199% of the median (450,360-746,847 SEK), and ≥200% of the median (750,600 SEK). Area of residence and occupation barely changed the estimates; therefore, they were not included in the models as confounders.
