# Supplementary material for: Unequal access? Use of sickness absence benefits by precariously employed workers with common mental disorders: a register-based cohort study in Sweden
Source: BMJ Open. 2023 Jul 19;13(7):e072459. doi: 10.1136/bmjopen-2023-072459 (PMC10357787; doi:10.1136/bmjopen-2023-072459)
Supplement: Author's manuscript [file bmjopen-2023-072459.draft_revisions.pdf]

BMJ Open

BMJ Open is committed to open peer review. As part of this commitment we make the peer review history of every article we publish publicly available.

When an article is published we post the peer reviewers' comments and the authors' responses online. We also post the versions of the paper that were used during peer review. These are the versions that the peer review comments apply to.

The versions of the paper that follow are the versions that were submitted during the peer review process. They are not the versions of record or the final published versions. They should not be cited or distributed as the published version of this manuscript.

BMJ Open is an open access journal and the full, final, typeset and author-corrected version of record of the manuscript is available on our site with no access controls, subscription charges or pay-per-view fees (http://bmjopen.bmj.com).

If you have any questions on BMJ Open's open peer review process please email info.bmjopen@bmj.com

BMJ Open

Unequal access? Use of sickness absence benefits by precarious workers with common mental disorders: A register-based cohort study in Sweden

Journal:	*BMJ Open*
Manuscript ID	bmjopen-2023-072459
Article Type:	Original research
Date Submitted by the Author:	03-Feb-2023

Complete List of Authors:	Hernando-Rodriguez, Julio C.; Karolinska Institutet, Unit of Occupational and Environmental Medicine, Institute of Environmental Medicine<br>Matilla-Santander, Nuria; Karolinska Institutet, Unit of Occupational and Environmental Medicine, Institute of Environmental Medicine<br>Murley, Chantelle; Karolinska Institutet, Division of Insurance Medicine, Department of Clinical Neuroscience<br>Blindow, Katrina; Karolinska Institutet, Unit of Occupational and Environmental Medicine, Institute of Environmental Medicine<br>Kvart, Signild; Karolinska Institutet, Unit of Occupational and Environmental Medicine, Institute of Environmental Medicine<br>Almroth, Melody; Karolinska Institutet, Unit of Occupational and Environmental Medicine, Institute of Environmental Medicine; Stockholm University, Department of Public Health Sciences<br>Kreshpaj, Bertina; Karolinska Institutet, Unit of Occupational and Environmental Medicine, Institute of Environmental Medicine; University of Copenhagen, Section of Epidemiology, Department of Publich Health<br>Thern, Emelie; Karolinska Institute, Unit of Occupational Medicine, Institute of Environmental Medicine<br>Badarin, Kathryn; Karolinska Institutet, Unit of Occupational Medicine, Institute of Environmental Medicine<br>Muntaner, Carles; University of Toronto, Lawrence S. Bloomberg Faculty of Nursing; University of Toronto, Dalla Lana School of Public Health<br>Gunn, Virginia; Karolinska Institutet, Unit of Occupational and Environmental Medicine, Institute of Environmental Medicine; St Michael's Hospital Centre for Urban Health Solutions, Li Ka Shing Knowledge Institute, Unity Health Toronto<br>Padrosa, Eva; Consorci Parc de Salut MAR de Barcelona, IMIM, SDHEd (Social Determinants and Health Education Research)<br>Julià, Mireia; Consorci Parc de Salut MAR de Barcelona, IMIM, SDHEd (Social Determinants and Health Education Research)<br>Méndez-Rivero, Fabrizio; Universitat Pompeu Fabra<br>Bodin, Theo; Karolinska Institutet, Unit of Occupational and Environmental Medicine, Institute of Environmental Medicine; Centre for Occupational and Environmental Medicine, Stockholm Region

Keywords:	MENTAL HEALTH, EPIDEMIOLOGY, OCCUPATIONAL & INDUSTRIAL MEDICINE, PUBLIC HEALTH

For peer review only - http://bmjopen.bmj.com/site/about/guidelines.xhtml

I, the Submitting Author has the right to grant and does grant on behalf of all authors of the Work (as defined in the below author licence), an exclusive licence and/or a non-exclusive licence for contributions from authors who are: i) UK Crown employees; ii) where BMJ has agreed a CC-BY licence shall apply, and/or iii) in accordance with the terms applicable for US Federal Government officers or employees acting as part of their official duties; on a worldwide, perpetual, irrevocable, royalty-free basis to BMJ Publishing Group Ltd ("BMJ") its licensees and where the relevant Journal is co-owned by BMJ to the co-owners of the Journal, to publish the Work in this journal and any other BMJ products and to exploit all rights, as set out in our licence.

The Submitting Author accepts and understands that any supply made under these terms is made by BMJ to the Submitting Author unless you are acting as an employee on behalf of your employer or a postgraduate student of an affiliated institution which is paying any applicable article publishing charge ("APC") for Open Access articles. Where the Submitting Author wishes to make the Work available on an Open Access basis (and intends to pay the relevant APC), the terms of reuse of such Open Access shall be governed by a Creative Commons licence – details of these licences and which Creative Commons licence will apply to this Work are set out in our licence referred to above.

Other than as permitted in any relevant BMJ Author's Self Archiving Policies, I confirm this Work has not been accepted for publication elsewhere, is not being considered for publication elsewhere and does not duplicate material already published. I confirm all authors consent to publication of this Work and authorise the granting of this licence.

For peer review only

Unequal access? Use of sickness absence benefits by precarious workers with common mental disorders: A register-based cohort study in Sweden

Julio C. Hernando-Rodriguez, PhD[1], Nuria Matilla-Santander, PhD[1], Chantelle Murley, PhD[2], Katrina Blindow, MPH[1], Signild Kvart, MSc[1], Melody Almroth, PhD[1,3], Bertina Kreshpaj, PhD[1,4], Emelie Thern, PhD[1], Kathryn Badarin, PhD[1], Carles Muntaner, PhD[5,6,7], Virginia Gunn, PhD[1,8,9], Eva Padrosa, PhD[10,11,12], Mireia Julià, PhD[10,11,12], Fabrizio Méndez-Rivero, MSc[12], Theo Bodin, PhD[1,13]

[1]Unit of Occupational Medicine, Institute of Environmental Medicine, Karolinska Institutet, Stockholm, Sweden; [2]Division of Insurance Medicine, Department of Clinical Neuroscience, Karolinska Institutet; Stockholm, Sweden; [3]Department of Public Health Sciences, Stockholm University; [4]Section of Epidemiology, Department of Public Health, University of Copenhagen, Copenhagen, Denmark; [5]Lawrence S. Bloomberg Faculty of Nursing, St. George Campus, Toronto, Canada; [6]Dalla Lana School of Public Health, University of Toronto, Toronto, Canada; [7]Department of Mental Health, The Johns Hopkins University Bloomberg School of Public Health, Baltimore, USA; [8]MAP Centre for Urban Health Solutions, Li Ka Shing Knowledge Institute, Unity Health Toronto, Toronto, Canada; [9]School of Nursing, Cape Breton University, Nova Scotia, Canada; [10]ESIMar (Mar Nursing School), Parc de Salut Mar, Universitat Pompeu Fabra-affiliated, Barcelona, Spain; [11]SDHEd (Social Determinants and Health Education Research Group), IMIM (Hospital del Mar Medical Research Institute), Barcelona, Spain; [12]GREDS (Research Group on Health Inequalities, Environment, and Employment Conditions Network), Universitat Pompeu Fabra, Barcelona, Spain; [13]Centre for Occupational and Environmental Medicine, Stockholm Region, Stockholm, Sweden

Corresponding author: Unit of Occupational Medicine, The Institute of Environmental Medicine (IMM), Karolinska Institutet, Solnavägen 4, 113 65, Stockholm, Sweden. julio.hernando@ki.se

Word count text (from introduction to conclusion): 3,533.

Number of tables: 3 (also 4 tables in the supplementary material in another file).

Number of figures: 2 (in another file).

For peer review only

For peer review only - http://bmjopen.bmj.com/site/about/guidelines.xhtml

ABSTRACT

Unequal access? Use of sickness absence benefits by precariously employed workers with common mental disorders: A register-based cohort study in Sweden

Objective

This study compares the use of sickness absence benefits (SABs) due to a common mental disorder (CMD) between precarious and non-precarious workers with CMD.

Design

Register-based cohort study.

Participants

The study included 78,215 Swedish workers between the ages of 27 and 61 residing in Sweden who experienced CMD in 2017, indicated by a new treatment with Selective Serotonin Reuptake Inhibitors (SSRI). Excluded were those who emigrated or immigrated, were self-employed, had an annual employment-based income < 100 Swedish krona, had > 90 days of unemployment per year, had student status, had SABs due to CMD during the exposure measurement (2016) and the two previous years, had an SSRI prescription one year or less before the start of the SSRI prescription in 2017, had packs of > 100 pills of SSRI medication, had a disability pension before 2017, were not entitled to SABs due to CMD in 2016, and had no information about the exposure.

Outcome

The first incidence of SABs due to CMD in 2017.

Results

The use of SABs due to a CMD was slightly lower among workers in PER compared to those in SER [adjusted Odds Ratio (aOR) 0.92, 95% confidence interval (CI) 0.81-1.05]. Particularly, women with three consecutive years in PER had reduced SABs use [aOR 0.48, 95% CI 0.26-0.89], while men in PER showed weaker evidence of association. Those in SER/HI also showed a lower use of SABs [aOR 0.74, 95% CI 0.67-0.81]. Low unionization and low and high-income levels were associated with lower use of SABs, particularly among women.

Conclusions

Given that workers with CMD in PER have a lower probability of using SABs, especially women, this study indicates a need for policies aimed at (i) guaranteeing access to SABs for people in PER and/or (ii) reducing involuntary forms of presenteeism.

Key terms: employment quality; precarious employment; SSRI; anxiety; depression; stress-related disorders; mental disorders; mental health; sick leave; welfare state; social insurance system; labour market; epidemiology; Sweden; longitudinal study; presenteeism.

For peer review only - http://bmjopen.bmj.com/site/about/guidelines.xhtml

Strengths and limitations of this study:

- This study is based on population registers with high validity and low attrition rates, which allows for an accurate and objective measure of precarious employment.

- The linkage of work disability and pharmaceutical registers allows for creating an accurate measurement of sickness absence for people with a new treatment for CMD.

- The longitudinal design of this study reduces inverse causation.

- People with three years of exposure to precarious employment before sickness absence tend to be healthier than those with less exposure time, which could lead to an underestimation of the results. However, we also analysed one year of exposure, finding comparable results that may overcome this limitation.

For peer review only

For peer review only - http://bmjopen.bmj.com/site/about/guidelines.xhtml

BACKGROUND

In the past decades, many countries, including Sweden, have experienced changes in labour market policies that prioritize organizational flexibility over employment security [1]. This led to an increase in precarious employment (PE) [2], which is characterized by employment insecurity, insufficient income, and a lack of rights and social protection [3,4]. The impact of these labour market changes on the health of the workforce and the use of sickness absence benefits (SABs) is not fully understood, especially, among workers in PE.

Mental disorders are one of the major causes of the global burden of disease [5]. In Sweden, half of the working-age population experiences a mental health condition during their lifetime; every year, 1 million workers have a mental disorder [6]. As a result, mental disorders are one of the leading diagnoses causing sickness absence (SA), especially among young people [7]. SA rates are not only driven by the health and work capacity of the working population but are influenced by contextual factors. Therefore, it has been suggested that low rates of SA in insecure jobs may reflect higher levels of presenteeism (i.e., working while being ill) due to workers' fear of dismissal rather than better health status [8,9].

In Sweden, the proportion of the working-age population who are at risk of not being entitled to SA is among the lowest (less than 1%) in the European Union [10]. Those who are not entitled to SA are often family workers employed in small family businesses and who do not receive a formal salary and, therefore, do not pay social contributions [10]. However, in 2016, the Swedish Social Insurance Inspectorate (ISF) deemed temporary employees' access to SABs to be legally uncertain, mainly due to the design of regulations that leave room for interpretation [11]. Workers without fixed schedules are particularly affected because this causes ambiguity as to what hours they would have worked had they not been sick, which opens the possibility to consider non-working periods as unemployment. The regulations of SABs are different for employees and unemployed persons, including entitlement requirements and the level of compensation. Moreover, the ISF found that temporary employees had fewer days of SABs than those permanently employed.

Previous studies have examined demographic characteristics such as age and gender, singular employment characteristics, or work environment factors in relation to SA [12]. To our knowledge, only two previous cross-sectional studies have analysed the relationship between PE and SA, and both of which found a positive association [13,14]. This is in line with previous studies confirming a relationship between PE and poor health, including a systematic review of longitudinal studies [15]. A recent Swedish study using the same data sources as the present study found that trajectories with constant PE increased the risk of common mental disorders (CMD – stress-related disorders, depression, and anxiety) [16].

Accordingly, we hypothesise that workers in PE would have a higher likelihood of using SABs due to CMD when compared to those in standard employment due to their worse mental health status. However, workers in PE who formally qualify for SABs may still experience barriers to accessing their rights. To investigate the use of SABs, we include only individuals who have recently initiated treatment for CMD and who formally qualify for SABs.

This study aims to compare the use of SABs due to CMD between precariously employed and non-precariously employed workers with CMD.

METHODS

For peer review only - http://bmjopen.bmj.com/site/about/guidelines.xhtml

Study population

This register-based study is based on the Swedish Work, Illness, and Labour-market Participation (SWIP) cohort of the total Swedish population. The SWIP cohort is obtained through the linkage of multiple registers and includes all individuals aged 16-65 years who were registered in Sweden in 2005 (around 5.7 million individuals), with follow-up until 2017. This study uses a subpopulation of 78,215 individuals aged 27-61 years in 2016 who experienced a CMD in 2017, indicated by the initiation of treatment with Selective Serotonin Reuptake Inhibitors (SSRIs). Included individuals had similar characteristics to the Swedish workforce, except for the higher share of women (Supplementary Table 1).

Sociodemographic and employment data were obtained from the Longitudinal Integration Database for Health Insurance and Labour Market Studies (LISA) for 2016. Data on SA (from day 15 onwards) were taken from the Micro Data for Analysis of Social Insurance (MiDAS) registers and included information such as ICD-10 diagnosis and length of SA for 2017. The Swedish Prescribed Drug Register informed the prescribed medicines dispensed from pharmacies for 2017.

Exclusion criteria were: (i) emigration or immigration (2012-2016), (ii) self-employment during 2014-2017 because SABs regulations differ [17], (iii) annual employment-based income < 100 SEK to ensure working status, (iv) > 90 days of unemployment per year to avoid measuring the effect of unemployment on the outcome, (v) student status during the autumn term to avoid misclassification as PE, (vi) SA due to CMD during the exposure measurement (2016) and the two previous years (2014-2015) to ensure that previous SA due to CMD does not influence the outcome, (vii) SSRI prescription one year or less before the start of SSRI prescription in 2017 [18,19], (viii) packs of > 100 pills of SSRI medication in 2017, as this is uncommon for new SSRI treatment, (ix) disability pension before 2017, (x) no entitlement to SABs due to CMD in 2016, and (xi) incomplete information on the exposure variable in 2016 (2014-2016 in the sensitivity analysis) (Figure 1).

Exposure variable

The PE measure was based on the second version of the Swedish Register-based Operationalization of Precarious Employment (SWE-ROPE) [20]. The SWE-ROPE considers five items (i.e., contractual relationship insecurity, contractual temporariness, multiple jobs/economic sectors, income level and lack of unionization) that represent the three dimensions of PE (i.e., employment insecurity, income inadequacy and lack of rights and protection). For each individual, an annual score for each of the five items was calculated [21]. Scores ranged by item: contractual employment insecurity (agency employed to directly employed: from -1 and 0); contractual temporariness (unstable employment and stable employment: -2 and 0); multiple jobs/economic sectors (multiple jobs and sectors, multiple jobs, a single job: -2 to 0), income level (< 60% of the median, 60-79%, 80-119%, 120-200% and > 200%: -2 to +2) and lack of unionization (< 70% of collective bargaining agreement coverage, 70-90% and > 90%: -2 to 0). The median income (375,300 SEK in 2016) was based on the Swedish working population after applying the inclusion and some of the main exclusion criteria (i to vii and xi described above).

A final summative score was obtained by summing the total score of the five items. Lower final scores suggest PE, while a higher score suggests non-PE. The final summative score for 2016 was categorised into four employment quality typologies: precarious employment relationship (PER; score<-3), sub-standard employment relationship (SSER; a score between -3 and -1), standard

For peer review only - http://bmjopen.bmj.com/site/about/guidelines.xhtml

employment relationship (SER; score=0) and standard employment relationship with high income (SER/HI; score>0). The SER/HI group was defined in light of previous literature [16,22] and included more than 85% of individuals with high-income levels (120-199% of the median income).

Outcome variable

The outcome was the first occurrence of SABs due to CMD starting in the 2 months spanning the month before and after the SSRI prescription date in 2017 (categorical outcome: 0, having SSRI and no SABs; 1, having SSRI co-occurring with SABs). This period was selected based on literature observing the effect of SSRI treatment after 3-4 weeks, so the risk of experiencing a new SA spell after that time may decrease [23–25]. The SSRI prescriptions included antidepressants from N06AB02 to N06AB10 according to the Anatomical Therapeutic Chemical (ATC) classification system. SA due to CMD was identified by ICD-10 codes: depression (F32-F33), anxiety (F41) and stress-related disorders (F43) including exhaustion disorder, which is conceptually similar to burnout. This definition of CMD is based on a previous paper about PE and the risk of CMD [16]. Regarding the SABs, all people living in Sweden aged 16 and over who receive a minimum annual income from work of at least 24% of the price base amount (10,752 SEK in 2017) and who have a reduced work capacity of at least 25% of their usual working hours due to disease or injury are covered by the SA insurance system [26]. A physician's certificate is required after the first seven days of SA. SABs replace up to 80% of lost work income. After the first qualifying day without compensation, the employer pays SA for the first 13 days. Therefore, SA spells of less than 15 days are not registered in MiDAS. From the 15th day onwards, SABs are paid by the Social Insurance Agency. For the present study, SABs due to CMD included only SA spells of at least 15 days.

Potential confounding variables

At baseline, age, education level, country of birth, family composition (civil status and having children), unemployment duration, economic sector, area of residence, occupation, and comorbidity (2016). Comorbidity was measured as having SA due to any diagnoses other than CMD since SA episodes in the previous years showed to be a predictor for future episodes of SA [27].

Statistical analysis

Logistic regression models estimated the association between employment quality typologies and the use of SABs due to CMD in 2016. A sensitivity analysis was conducted by conditioning models to have the same exposure for three consecutive years (2014-2016) to observe if stability could influence the results. Models were adjusted for the potential confounders mentioned above and identified using a Directed Acyclic Graph (DAG) (Figure 2). All analyses were conducted for the total population as well as women and men separately. The analysis is separated by gender because previous studies have found different patterns in SA among women and men [28,29]. Women have higher SA rates compared to men possibly due to the higher exposure to an unequal division of domestic work combined with paid work [28]. Work-family conflicts are related to higher ORs to report short SA (1-7 days) among men and longer SA among women (8-30 days) [29].

Data management and statistical analysis were performed using Stata SE v17.

For peer review only - http://bmjopen.bmj.com/site/about/guidelines.xhtml

Patient and public involvement

Patients and/or the public were not involved in the design, or conduct, or reporting, or dissemination plans of this research.

RESULTS

People in PER (5.10%) had a higher proportion of young-middle aged individuals (27-40 years) (59.4%), women (69.7%), low educational attainment (63.2%), born in Sweden (88.4%), couples with children (44.4%), zero days unemployed (85.6%), private sector (79.6%), unstable employment (93.5%), multiple job holding in different economic sectors (20.7%), income level below 60% of the median (47.5%), and low unionization (22.6%) (Table 1). The proportion of people having a first spell of SABs due to CMD in 2017 was 7.5% among our study population and showed differences according to sociodemographic and employment characteristics in general (Supplementary Table 2), and by gender and employment quality typology (Supplementary Tables 3 and 4, respectively).

For peer review only

Table 1. Sociodemographic and employment characteristics at baseline (2016) in a cohort of 78,215 individuals with common mental disorders, aged 27-61, entitled to sickness absence benefits in 2016 by employment quality typology (Standard Employment Relationship, SER; Standard Employment Relationship with high income, SER/HI; Sub-standard Employment Relationship, SSER; Precarious Employment Relationship, PER; baseline, 2016).

	SER/HI (14.68%)	SER (38.04%)	SSER (42.18%)	PER (5,10%)
	N (%)	N (%)	N (%)	N (%)
**Age**				
27-40 years	2,878 (25.1)	9,817 (33.0)	15,612 (47.3)	2,368 (59.4)
41-51 years	5,239 (45.6)	11,432 (38.4)	10,844 (32.9)	1,026 (25.7)
52-61 years	3,366 (29.3)	8,504 (28.6)	6,537 (19.8)	592 (14.9)
**Gender**				
Male	5,999 (52.2)	10,133 (34.1)	8,554 (25.9)	1,208 (30.3)
Female	5,484 (47.8)	19,620 (65.9)	24,439 (74.1)	2,778 (69.7)
**Education level**				
Elementary/high school education	3,395 (29.6)	15,831 (53.2)	19,329 (58.6)	2,517 (63.2)
Higher education <3 years	1,989 (17.3)	4,384 (14.7)	4,446 (13.5)	637 (16.0)
Higher education ≥3 years	6,092 (53.1)	9,528 (32.0)	9,201 (27.9)	830 (20.8)
**Country of birth**				
Sweden	10,449 (91.0)	26,561 (89.3)	29,203 (88.6)	3,523 (88.4)
Not Sweden	1,031 (9.0)	3,174 (10.7)	3,770 (11.4)	463 (11.6)
**Family composition**				
Couple with children	6,045 (52.6)	13,320 (44.8)	16,338 (49.5)	1,768 (44.4)
Couple without children	1,354 (11.8)	3,630 (12.2)	3,151 (9.6)	286 (7.2)
Single with children	1,252 (10.9)	3,903 (13.1)	4,528 (13.7)	560 (14.0)
Single without children	2,832 (24.7)	8,900 (29.9)	8,976 (27.2)	1,372 (34.4)
**Unemployment**				
0 days unemployed	11,429 (99.5)	29,514 (99.2)	31,733 (96.2)	3,414 (85.6)
From 1 to 90 days unemployed	54 (0.5)	239 (0.8)	1260 (3.8)	572 (14.4)
**Economic sector**				
Private	6,995 (60.9)	13,908 (46.7)	17,578 (53.3)	3,172 (79.6)
Public	4,488 (39.1)	15,845 (53.3)	15,415 (46.7)	814 (20.4)
**Contractual relationship insecurity**				
Directly employed by an employer	11,473 (99.9)	29,715 (99.9)	32,529 (98.6)	3,765 (94.5)
Employed by an agency	10 (0.1)	38 (0.1)	464 (1.4)	221 (5.5)
**Contractual temporariness**				
Stable employment	11,483 (100.0)	29,282 (98.4)	18,070 (54.8)	260 (6.5)
Unstable employment	(0.0)	471 (1.6)	14,923 (45.2)	3,726 (93.5)
**Multiple jobs/economic sectors**				
1-2 jobs	11,143 (97.0)	29,123 (97.9)	30,584 (92.7)	2,168 (54.4)
3 or more jobs	221 (1.9)	440 (1.5)	1,916 (5.8)	994 (24.9)
3 or more jobs in 3 or more sectors	119 (1.0)	190 (0.6)	493 (1.5)	824 (20.7)
**Income level***				
<60% of the median	(0.0)	(0.0)	3,320 (10.1)	1,892 (47.5)
60-79% of the median	(0.0)	(0.0)	14,823 (44.9)	1,153 (28.9)
80-119% of the median	(0.0)	28,657 (96.3)	10,708 (32.5)	878 (22.0)
120-200% of the median	9,849 (85.8)	590 (2.0)	3,960 (12.0)	62 (1.6)
>200% of the median	1,634 (14.2)	506 (1.7)	182 (0.6)	1 (<0.1)
**Unionization level**				
<70%	(0.0)	20 (0.1)	852 (2.6)	901 (22.6)
70-90%	55 (0.5)	412 (1.4)	3,297 (10.0)	866 (21.7)
>90%	11,428 (99.5)	29,321 (98.5)	28,844 (87.4)	2,219 (55.7)

Missing values in the educational level (SER/HI: 7, 0.06%; SER: 10, 0.03%; SSER: 17, 0.05%; PE: 2, 0.05%) and country of birth (SER/HI: 3, 0.03%; SER: 18, 0.06%; SSER: 20, 0.06). Persons entitled to sickness absence benefits in 2016 are those having an annual income from paid employment of at least 24% of the price base amount of 2016; that is, 10,632 SEK. *Income level categories based on the median of the Swedish working population (375,300 SEK) after applying several inclusion and exclusion criteria (see methods section). Income category boundaries: <60% of the median (225,180 SEK), 60-79% of the median (225,180-296,487 SEK), 80-119% of the median (300,240-446,607 SEK), 120-199% of the median (450,360-746,847 SEK), and ≥200% of the median (750,600 SEK).

For peer review only

The association between the employment quality typologies and the use of SABs due to CMD showed an inverted U-shape pattern, with lower adjusted Odds Ratio (aOR) estimates found among individuals with an SER/HI, followed by near-significance estimates of those in a PER, compared to the SER group (aOR: 0.74, 95% CI: 0.67-0.81 and aOR: 0.92, 95% CI: 0.81-1.05, respectively) (Table 2). The SSER group showed no significant differences compared to the SER group. Women were less likely to use SABs due to CMD in the PER (OR: 0.88, 95% CI: 0.75-1.04) and the SER/HI groups (aOR: 0.72, 95% CI: 0.63-0.83) compared to the SER group. Men in SSER and PER showed weaker evidence of association, except for those in the SER/HI group (aOR: 0.76, 95% CI: 0.66-0.86). Overall, regression models conditional on having the same exposure for three consecutive years showed similar results, in this case, lower use of SABs due to CMD occurred among individuals with SER/HI (aOR: 0.74, 95% CI: 0.65-0.85). Women with three consecutive years of PER had the lowest probability of using SABs due to CMD (aOR: 0.48, 95% CI: 0.26-0.89), followed by women and men with SER/HI compared to the SER group (aOR: 0.76, 95% CI: 0.62-0.92 and aOR: 0.73, 95% CI: 0.61-0.88, respectively). Given that area of residence and occupation barely changed the estimates, they were not included in the models as confounders.

Table 2. Association between employment quality typologies (Standard Employment Relationship, SER; Standard Employment Relationship with high income, SER/HI; Sub-standard Employment Relationship, SSER; Precarious Employment Relationship, PER; baseline, 2016) and sickness absence benefits due to common mental disorders in 2016 among people entitled to sickness absence benefits in 2016 (N=78,215).

					Models with three years of continuous exposure* (N=38,761)			
	**N**	**Cases N (%)**	**OR (95% CI)**	**aOR (95% CI)**	**N**	**Cases N (%)**	**OR (95% CI)**	**aOR (95% CI)**
**Employment quality typology**								
SER	29,753	2,268 (7.6)	1	1	17,353	1,207 (7.0)	1	1
SER/HI	11,483	593 (5.2)	0.66 (0.60-0.72)	0.74 (0.67-0.81)	6,924	330 (4.8)	0.67 (0.59-0.76)	0.74 (0.65-0.85)
SSER	32,993	2,667 (8.1)	1.07 (1.01-1.13)	0.99 (0.93-1.05)	14,065	1,055 (7.5)	1.08 (1.00-1.18)	1.01 (0.92-1.10)
PER	3,986	320 (8.0)	1.06 (0.94-1.20)	0.92 (0.81-1.05)	419	24 (5.7)	0.81 (0.54-1.23)	0.74 (0.48-1.12)
**Women (N=52,321)**								
SER	19,620	1,375 (7.0)	1	1	11,137	715 (6.4)	1	1
SER/HI	5,484	247 (4.5)	0.63 (0.54-0.72)	0.72 (0.63-0.83)	2,954	129 (4.4)	0.67 (0.55-0.81)	0.76 (0.62-0.92)
SSER	24,439	1,892 (7.7)	1.11 (1.04-1.20)	1.00 (0.93-1.08)	11,138	810 (7.3)	1.14 (1.03-1.27)	1.00 (0.90-1.12)
PER	2,778	198 (7.1)	1.02 (0.87-1.19)	0.88 (0.75-1.04)	296	11 (3.7)	0.56 (0.31-1.03)	0.48 (0.26-0.89)
**Men (N=25,894)**								
SER	10,133	893 (8.8)	1	1	6,216	492 (7.9)	1	1
SER/HI	5,999	346 (5.8)	0.63 (0.56-0.72)	0.76 (0.66-0.86)	3,970	201 (5.1)	0.62 (0.52-0.73)	0.73 (0.61-0.88)
SSER	8,554	775 (9.1)	1.03 (0.93-1.14)	0.97 (0.88-1.08)	2,927	245 (8.4)	1.06 (0.91-1.25)	1.01 (0.86-1.19)
PER	1,208	122 (10.1)	1.16 (0.95-1.42)	1.02 (0.83-1.25)	123	13 (10.6)	1.37 (0.77-2.46)	1.31 (0.73-2.36)

OR: unadjusted Odds Ratio; aOR: Odds Ratio adjusted by age, sex, education, country of birth, family composition, economic sector, unemployment, and SA due to diagnoses other than CMD in the baseline (2016); *Models conditioned to have the same exposure for three consecutive years (2014-2016). Note: Persons entitled to sickness absence benefits are those having an annual income from paid employment of at least 24% of the price base amount of 2016; that is, 10,632 SEK. Area of residence and occupation barely changed the estimates; therefore, they were not included in the models as confounders.

For peer review only - http://bmjopen.bmj.com/site/about/guidelines.xhtml

The employment quality items most strongly related to the use of SABs due to CMD were income and unionization levels (Table 3). In the fully adjusted model, high income was associated with a reduced probability of SABs due to CMD (120-199% of the median income - aOR: 0.79, 95% CI: 0.72-0.85; and more than 199% of the median income - aOR: 0.61, 95% CI: 0.49-0.75), while the lowest-income level showed slight differences (lower than 60% of the median income – aOR: 0.92, 95% CI: 0.82-1.03). Women with the highest-income levels exhibit lower aORs compared to men (aOR: 0.48, 95% CI: 0.33-0.71 and aOR: 0.71, 95% CI: 0.54-0.92, respectively). Low unionization coverage was associated with lower use of SABs (less than 70% of unionization level - aOR: 0.82, 95% CI: 0.68-0.99), particularly among women (0.77, 95% CI: 0.58-1.03, near to significance). Similar results were found for women and men with medium unionization coverage.

For peer review only

Table 3. Association between employment quality dimensions (2016, baseline) and sickness absence benefits due to CMD in 2016 (N=78,215) among people entitled to sickness absence benefits in 2016.

					Women (N=52,321)				Men (N=25,894)			
	**Cases N(%)**	**OR (95% CI)**	**aOR (95% CI) [1]**	**aOR (95% CI) [2]**	**Cases N(%)**	**OR (95% CI)**	**aOR (95% CI) [1]**	**aOR (95% CI) [2]**	**Cases N(%)**	**OR (95% CI)**	**aOR (95% CI) [1]**	**aOR (95% CI) [2]**
**Contractual relationship insecurity**												
Directly employed by an employer	5,788 (7.5)	1	1	1	3,683 (7.1)	1	1	1	2,105 (8.2)	1	1	1
Employed by an agency	60 (8.2)	1.10 (0.85-1.44)	1.05 (0.80-1.37)	0.96 (0.73-1.25)	29 (6.5)	0.91 (0.62-1.32)	0.89 (0.61-1.31)	0.85 (0.58-1.24)	31 (10.9)	1.36 (0.94-1.98)	1.19 (0.81-1.73)	1.08 (0.74-1.58)
**Contractual temporariness**												
Stable employment	4,351 (7.4)	1	1	1	2,792 (7.1)	1	1	1	1,559 (8.0)	1	1	1
Unstable employment	1,497 (7.8)	1.07 (1.01-1.14)	1.08 (1.02-1.15)	1.02 (0.95-1.09)	920 (7.2)	1.03 (0.95-1.11)	1.06 (0.98-1.15)	1.01 (0.92-1.10)	577 (9.0)	1.14 (1.03-1.26)	1.12 (1.01-1.24)	1.04 (0.93-1.16)
**Multiple jobs/economic sectors**												
1-2 jobs	5,436 (7.4)	1	1	1	3,463 (7.1)	1	1	1	1,973 (8.2)	1	1	1
3 or more jobs	279 (7.8)	1.05 (0.93-1.19)	1.06 (0.93-1.20)	1.05 (0.93-1.20)	175 (7.0)	0.99 (0.85-1.16)	1.02 (0.87-1.20)	1.00 (0.85-1.18)	104 (9.6)	1.19 (0.97-1.47)	1.17 (0.95-1.45)	1.18 (0.95-1.46)
3 or more jobs in 3 or more sectors	133 (8.2)	1.11 (0.93-1.33)	1.11 (0.93-1.34)	1.09 (0.91-1.31)	74 (7.3)	1.04 (0.82-1.32)	1.07 (0.84-1.36)	1.04 (0.81-1.32)	59 (9.6)	1.19 (0.91-1.56)	1.16 (0.88-1.53)	1.19 (0.90-1.57)
**Income level**												
80-119% of the median	3,113 (7.7)	1	1	1	1,876 (7.1)	1	1	1	1,237 (8.9)	1	1	1
<60% of the median	395 (7.6)	0.98 (0.88-1.09)	0.98 (0.88-1.10)	0.92 (0.82-1.03)	319 (7.3)	1.03 (0.91-1.17)	1.05 (0.93-1.19)	0.91 (0.81-1.04)	76 (8.9)	1.00 (0.78-1.27)	0.99 (0.77-1.26)	0.94 (0.73-1.20)
60-79% of the median	1,415 (8.9)	1.16 (1.09-1.24)	1.17 (1.09-1.24)	1.10 (1.03-1.18)	1,111 (8.4)	1.20 (1.11-1.30)	1.21 (1.12-1.31)	1.08 (1.00-1.17)	304 (10.9)	1.24 (1.09-1.42)	1.25 (1.10-1.43)	1.16 (1.01-1.33)
120-200% of the median	831 (5.8)	0.73 (0.67-0.79)	0.72 (0.67-0.78)	0.79 (0.72-0.85)	378 (5.1)	0.70 (0.63-0.79)	0.70 (0.63-0.79)	0.79 (0.70-0.88)	453 (6.4)	0.70 (0.62-0.78)	0.69 (0.62-0.78)	0.80 (0.71-0.89)
>200% of the median	94 (4.1)	0.50 (0.41-0.62)	0.50 (0.40-0.62)	0.61 (0.49-0.75)	28 (2.9)	0.39 (0.26-0.57)	0.39 (0.26-0.56)	0.48 (0.33-0.71)	66 (4.9)	0.52 (0.41-0.68)	0.51 (0.40-0.66)	0.71 (0.54-0.92)
**Unionization level**												
>90%	5,409 (7.5)	1	1	1	3,497 (7.2)	1	1	1	1,912 (8.3)	1	1	1
70-90%	315 (6.8)	0.90 (0.80-1.01)	0.86 (0.77-0.97)	0.82 (0.73-0.92)	161 (6.1)	0.84 (0.72-0.99)	0.82 (0.70-0.97)	0.83 (0.70-0.98)	154 (7.7)	0.92 (0.78-1.10)	0.84 (0.71-1.00)	0.80 (0.67-0.96)
<70%	124 (7.0)	0.92 (0.77-1.11)	0.87 (0.72-1.05)	0.82 (0.68-0.99)	54 (5.8)	0.80 (0.61-1.06)	0.76 (0.58-1.01)	0.77 (0.58-1.03)	70 (8.2)	0.99 (0.77-1.27)	0.87 (0.67-1.12)	0.84 (0.65-1.09)

OR: unadjusted Odds Ratio; [1] aOR: Odds Ratio adjusted mutually by the other employment quality dimensions in the baseline (2016); [2] aOR: Odds Ratio adjusted mutually by the other employment quality dimensions and age, sex, education, country of birth, family composition, economic sector, unemployment, and SA due to diagnoses other than CMD in the baseline (2016); Note: Persons entitled to sickness absence benefits are those having an annual income from paid employment of at least 24% of the price base amount of 2016; that is, 10,632 SEK. *Income level categories based on the median of the Swedish working population after applying the inclusion and some of the main exclusion criteria (see methods section). *Income level categories based on the median of the Swedish working population (375,300 SEK) after applying several inclusion and exclusion criteria (see methods section). Income category boundaries: <60% of the median (225,180 SEK), 60-79% of the median (225,180-296,487 SEK), 80-119% of the median (300,240-446,607 SEK), 120-199% of the median (450,360-746,847 SEK), and ≥200% of the median (750,600 SEK). Area of residence and occupation barely changed the estimates; therefore, they were not included in the models as confounders.

For peer review only - http://bmjopen.bmj.com/site/about/guidelines.xhtml

DISCUSSION

This study indicated that having a PER was associated with a lower probability of using SABs due to CMD compared to SER, especially among women. On the other side of the spectrum, we also found that SER/HI was strongly and negatively associated with SABs. The income and unionization level were the themes of employment quality most strongly related to using SABs due to CMD: having high income and low unionization levels were associated with lower use of SABs due to CMD, particularly, among women.

Our results on the negative association between PER and the use of SABs due to CMD contrast with previous studies that found an increased prevalence of self-reported SA due to health problems among precariously employed workers, in Sweden and other countries [13,14]. In our present study, sickness presenteeism (SP) may explain the lower odds of SABs due to CMD in the PER group. Precariously employed workers who typically have low-income jobs are likely to incur SP because they cannot afford to use SABs. Using SABs results in a period of reduced income since the SABs only partially replace wages [30]. Additionally, SP may stem from the fear of being dismissed due to employment insecurity or from the lack of union protection [9,31]. An earlier study found that workers who transitioned from temporary to permanent employment had an increased probability of SA [8]. Precariously employed workers in our study population had the lowest unionization levels and low unionization was related to lower use of SABs due to CMD. These findings are consistent with previous studies in Germany and Norway where trade union members had higher SA than non-members (45,46). A second possible explanation for the low use of SABs among precariously employed workers is related to legal uncertainty when the Social Insurance Agency assesses entitlement to SABs for non-standard employees [32]. If temporary employees have scheduled work, they receive SABs for the days that they would have worked had the illness not reduced their work capacity by at least 25%. Non-standard employees however often lack a formal schedule covering the days in question, e.g., on-demand employees get their shifts assigned on a day-to-day basis or have a schedule based on an oral agreement, and risk being considered unemployed in the event of sickness and cannot access SABs from the employer. To access SABs, work capacity must be assessed, and this is done differently according to employment status. The work capacity of employees is assessed only relative to their current employment for the first 90 days of SA and only later against all jobs in the entire labour market, but unemployed (or non-standard workers without scheduled hours) will be assessed relative to all jobs in the entire labour market from the start. In practice, this often means that they do not qualify for SABs, since other jobs may exist in the labour market where their current functional ability does not impose a sufficient reduction in work capacity. However, we may bear in mind that this explanation is less plausible for workers with CMD that may have a reduced work capacity for any task.

In general, women had lower use of SABs in any employment quality typology compared to the SER, and the association was more pronounced in women than in men. These results were unexpected since women usually have higher SA rates than men [33]. However, our study population only included individuals with SSRI treatment (two-thirds of them women), which has not been studied before with this study design [34]. The lower use of SABs among women is plausible because it has been suggested that women may feel a greater responsibility to their colleagues and avoid burdening them by cooperating more when performing work tasks compared to men [30]. Another study showed that female-dominated jobs related to jobs with responsibility for others such as education, human health and social work activities are more prone to SP [35]. Women with a consecutive 3-year period in PER had the lowest use of SABs.

For peer review only

This could be explained by the fact that women are more frequently employed in temporary employment, which has been related to fewer days of SABs than permanently employed and, probably, to lower access to SABs for the reasons previously mentioned [32].

SER/HI showed the highest income levels and we found that having a high income was related to a lower likelihood of using SABs. In this line, a Finnish study found that a higher position by education, occupational class and income was related to lower SA [36]. Another possible explanation for the lower use of SABs in employees in SER/HI is also SP, but for different reasons than the PER group. In a study conducted in Norway and Sweden, having supervisory responsibilities at work that no one else can perform was an influential factor for SP, which could be the case for the high earners in the present study [30]. Another driver for SP among high earners could be work satisfaction since they probably have jobs with more creativity, freedom and control [30,37]. Another explanation is that high earners have more knowledge-intensive occupations where they can work from home rather than take SA [38]. Lastly, individuals in SER/HI could plausibly be more likely to combine SSRIs with cognitive behavioural therapy (CBT) or mindfulness which could be, to a certain extent, protective of work capacity [39,40].

SA can also be conceptualised as a health status measure. Accordingly, an important consequence of SP is an increased risk of more severe and long-lasting health problems that, in turn, lead to longer periods of SA, regardless of the employment quality typology followed [41].

LIMITATIONS AND STRENGTHS

Limitations of this study could arise from the design of the study when analysing individuals with three years with the same exposure since those with PE are healthier enough to not exit the labour market, which could underestimate the results. However, we also analysed one year of exposure before SABs, finding comparable results which may overcome the previous limitation. We cannot rule out that some people in the study population were not entitled to SABs since apart from the prior income requirement, it is necessary to have a reduced work capacity due to the CMD of at least 25%, which is not possible to assess in the datasets. Selection bias could be introduced among SER/HI because CBT and other psychotherapies could be more prevalent in competing with SSRIs. It could be thought that we could have included a longer period for outcome measurement. However, our main interest is using SSRI as an indicator of having a new diagnosis of CMD and our approach is conservative in considering SABs close to SSRI since there is mixed evidence about the long-term efficacy of SSRIs [25,42]. Another limitation is that we did not have access to a level of detail of work to explore whether a person in non-standard arrangements is scheduled for work or is between work periods. Further research with alternative data sources is needed in this regard.

One of the strengths of this study is that it is based on population registers with high validity and low attrition rates, which allows for accurate and objective measurement of PE through a multi-dimensional measure [43]. Similarly, the linkage of pharmaceutical and work disability registers with the exact dates of SSRI prescription and SABs allowed for an accurate assessment of the outcome. The study is based on a large subpopulation of the Swedish working population. Lastly, the longitudinal study design reduces inverse causation.

GENERALIZABILITY

For peer review only - http://bmjopen.bmj.com/site/about/guidelines.xhtml

The results of this study are specific to our Swedish study population with a prescription of SSRIs and may not be generalisable to other countries with large variations in the treatments available for CMDs, subsidisation of SSRIs costs, and SAB regulations.

CONCLUDING REMARKS

This study found lower use of SABs among people with CMD in PER and SER/HI than in SER, particularly, women with a period of three consecutive years in PER, indicating that there could be barriers to accessing SABs among precariously employed. More inclusive policies are recommended to reduce these obstacles and guarantee SABs for those who are in PER and/or reduce involuntary forms of SP.

Contributors

TB conceived the initial idea of the study and act as a guarantor of the work. All authors contributed to the design of the study. TB and JCH-R further developed the study design and discussed it with NM-S. Data management and data analysis were performed by JCH-R with support from NM-S. All authors contributed to the interpretation of the results. JCH-R drafted the first version of the manuscript, and all authors made subsequent revisions of the work, agreed on the text and findings, and approved the final version. The corresponding author certifies that all listed authors meet authorship criteria.

Funding

This research was funded by FORTE—Swedish Research Council for Health Working Life and Welfare. Grant number 2021-00034.

Acknowledgements

The authors are grateful to all the researchers of the PWR Consortium and acknowledge Anette Linnersjö for ordering and receiving data and the valuable input on the data management and Cecilia Orellana for her feedback on the study design.

Competing interests

The authors declare no conflict of interest. The funder had no role in the design of the study; in the collection, analysis, or interpretation of data; in the writing of the manuscript, or in the decision to publish the results.

Patient and public involvement

Patients and/or the public were not involved in the design, or conduct, or reporting, or dissemination plans of this research.

Patient consent for publication

Not required.

Ethics approval

The Regional Ethics Board of Stockholm granted ethical permission for the study (no. 2017/1224-31/2 and 2018/1675-32). This is a register-based study and informed consent is not applicable.

Data availability statement

Data may be obtained from a third party and are not publicly available.

For peer review only - http://bmjopen.bmj.com/site/about/guidelines.xhtml

REFERENCES

1 Rodgers G. Labour Market Flexibility and Decent Work. ;:11.

2 Berglund T, Håkansson K, Isidorsson T. Occupational change on the dualised Swedish labour market. *Econ Ind Democr* 2022;**43**:918–42. doi:10.1177/0143831X20959714

3 Kreshpaj B, Orellana C, Burström B, *et al.* What is precarious employment? A systematic review of definitions and operationalizations from quantitative and qualitative studies. *Scand J Work Environ Health* 2020;**46**:235–47. doi:10.5271/sjweh.3875

4 Benach J, Muntaner C. Precarious employment and health: developing a research agenda. *J Epidemiol Community Health* 2007;**61**:276–7. doi:10.1136/jech.2005.045237

5 Global, regional, and national burden of 12 mental disorders in 204 countries and territories, 1990–2019: a systematic analysis for the Global Burden of Disease Study 2019. *Lancet Psychiatry* 2022;**9**:137–50. doi:10/gn7nxg

6 OECD. Mental Health and Work: Sweden. 2013. https://doi.org/10.1787/9789264188730-en (accessed 12 Jul 2022).

7 Swedish Social Insurance Agency [Försäkringskassan]. Social Insurance in Figures 2021. 2021.

8 Virtanen M, Kivimäki M, Elovainio M, *et al.* From insecure to secure employment: changes in work, health, health related behaviours, and sickness absence. *Occup Environ Med* 2003;**60**:948–53. doi:10.1136/oem.60.12.948

9 Johns G. Presenteeism in the workplace: A review and research agenda. *J Organ Behav* 2010;**31**:519–42. doi:10.1002/job.630

10 Matsaganis M, Özdemir E, Ward T, *et al.* Non-standard employment and access to social security benefits. 2015;:43.

11 Schill HP, Bjurström S, Pietrek J, *et al.* The Swedish sickness insurance and temporary employment. 2016;:5.

12 Allebeck P, Mastekaasa A. Chapter 5. Risk factors for sick leave - general studies. *Scand J Public Health* 2004;**32**:49–108. doi:10/dvr35c

13 Oke A, Braithwaite P, Antai D. Sickness Absence and Precarious Employment: A Comparative Cross-National Study of Denmark, Finland, Sweden, and Norway. *Int J Occup Environ Med* 2016;**7**:125–47. doi:10/gmx883

14 Matilla-Santander N, González-Marrón A, Martín-Sánchez JC, *et al.* Precarious employment and health-related outcomes in the European Union: a cross-sectional study. *Crit Public Health* 2020;**30**:429–40. doi:10.1080/09581596.2019.1587385

For peer review only

For peer review only - http://bmjopen.bmj.com/site/about/guidelines.xhtml

15 Ronnblad T, Gronholm E, Jonsson J, *et al.* Precarious employment and mental health: a systematic review and meta-analysis of longitudinal studies. *Scand J Work Env Health* 2019;**45**:429–43. doi:10.5271/sjweh.3797

16 Jonsson J, Muntaner C, Bodin T, *et al.* Low-quality employment trajectories and risk of common mental disorders, substance use disorders and suicide attempt: a longitudinal study of the Swedish workforce. *Scand J Work Env Health* 2021;**47**:208–16. doi:10/gmprvj

17 Sickness benefit for those with sole proprietorship. http://www.forsakringskassan.se/english/sick/sole-proprietorship/sickness-benefit-for-those-with-sole-proprietorship (accessed 29 Sep 2022).

18 Hutchins DS, Zeber JE, Roberts CS, *et al.* Initial Medication Adherence—Review and Recommendations for Good Practices in Outcomes Research: An ISPOR Medication Adherence and Persistence Special Interest Group Report. *Value Health* 2015;**18**:690–9. doi:10.1016/j.jval.2015.02.015

19 Aznar-Lou I, Iglesias-González M, Gil-Girbau M, *et al.* Impact of initial medication non-adherence to SSRIs on medical visits and sick leaves. *J Affect Disord* 2018;**226**:282–6. doi:10.1016/j.jad.2017.09.057

20 Bodin T, Jonsson J. Swedish Register Operationalization of Precarious Employment. 2020.https://github.com/TBodin/swe-rope (accessed 15 Mar 2021).

21 Jonsson J, Matilla-Santander N, Kreshpaj B, *et al.* Exploring multidimensional operationalizations of precarious employment in Swedish register data – a typological approach and a summative score approach. *Scand J Work Environ Health* 2021;**47**:117–26. doi:10.5271/sjweh.3928

22 Pollack R, Kreshpaj B, Jonsson J, *et al.* Low-quality employment trajectories and the risk of common mental health disorders among individuals with Swedish and foreign background - a register-based cohort study. *Scand J Work Environ Health* 2022;**48**:351–60. doi:10.5271/sjweh.4029

23 Overview - SSRI antidepressants. nhs.uk. 2021.https://www.nhs.uk/mental-health/talking-therapies-medicine-treatments/medicines-and-psychiatry/ssri-antidepressants/overview/ (accessed 21 Jul 2022).

24 Machado-Vieira R, Baumann J, Wheeler-Castillo C, *et al.* The Timing of Antidepressant Effects: A Comparison of Diverse Pharmacological and Somatic Treatments. *Pharmaceuticals* 2010;**3**:19–41. doi:10.3390/ph3010019

25 Cheng Q, Huang J, Xu L, *et al.* Analysis of Time-Course, Dose-Effect, and Influencing Factors of Antidepressants in the Treatment of Acute Adult Patients With Major Depression. *Int J Neuropsychopharmacol* 2020;**23**:76–87. doi:10.1093/ijnp/pyz062

26 Swedish Social Insurance Agency [Försäkringskassan]. Social Insurance in Figures 2020. 2020.

For peer review only - http://bmjopen.bmj.com/site/about/guidelines.xhtml

27 Roelen C a. M, Koopmans PC, Schreuder J a. H, *et al.* The history of registered sickness absence predicts future sickness absence. *Occup Med Oxf Engl* 2011;**61**:96–101. doi:10/ct43k7

28 Ármannsdóttir B, Mårdby A-C, Haukenes I, *et al.* Cumulative incidence of sickness absence and disease burden among the newly sick-listed, a cross-sectional population-based study. *BMC Public Health* 2013;**13**:329. doi:10.1186/1471-2458-13-329

29 Staland-Nyman C, Houkes I, de Rijk A, *et al.* Gender equality in domestic work and sickness absence - a population-based study on women and men in Sweden. *Women Health* 2021;**61**:325–36. doi:10.1080/03630242.2021.1872759

30 Johansen V, Aronsson G, Marklund S. Positive and negative reasons for sickness presenteeism in Norway and Sweden: a cross-sectional survey. *BMJ Open* 2014;**4**:e004123. doi:10.1136/bmjopen-2013-004123

31 Aronsson G, Gustafsson K. Sickness Presenteeism: Prevalence, Attendance-Pressure Factors, and an Outline of a Model for Research. *J Occup Environ Med* 2005;**47**:958–66.

32 Inspektionen för socialförsäkringen. Tidsbegränsade anställningar och sjukförsäkringen 2016. 2016.

33 *Socialförsäkringen i siffror 2022*. Stockholm: : Försäkringskassan 2022.

34 Lytsy P, Alexanderson K, Friberg E. Gender differences in treatment with antidepressants during first weeks of a sick-leave spell due to depressive episode. *Eur J Public Health* 2020;**30**:299–304. doi:10.1093/eurpub/ckz172

35 Marklund S, Gustafsson K, Bergström G, *et al.* Reasons for presenteeism in different occupational branches in Sweden: a population based cross-sectional study. *Int Arch Occup Environ Health* 2021;**94**:1385–95. doi:10.1007/s00420-021-01701-2

36 Piha K, Laaksonen M, Martikainen P, *et al.* Interrelationships between education, occupational class, income and sickness absence. *Eur J Public Health* 2010;**20**:276–80. doi:10.1093/eurpub/ckp162

37 Hansen CD, Andersen JH. Going ill to work – What personal circumstances, attitudes and work-related factors are associated with sickness presenteeism? *Soc Sci Med* 2008;**67**:956–64. doi:10.1016/j.socscimed.2008.05.022

38 Steidelmüller C, Meyer S-C, Müller G. Home-Based Telework and Presenteeism Across Europe. *J Occup Environ Med* 2020;**62**:998–1005. doi:10.1097/JOM.0000000000001992

39 Roshanaei-Moghaddam B, Pauly MC, Atkins DC, *et al.* Relative effects of CBT and pharmacotherapy in depression versus anxiety: is medication somewhat better for depression, and CBT somewhat better for anxiety? *Depress Anxiety* 2011;**28**:560–7. doi:10.1002/da.20829

For peer review only - http://bmjopen.bmj.com/site/about/guidelines.xhtml

For peer review only

40 Grossman P, Niemann L, Schmidt S, *et al.* Mindfulness-based stress reduction and health benefits: A meta-analysis. *J Psychosom Res* 2004;**57**:35–43. doi:10.1016/S0022-3999(03)00573-7

41 Taloyan M, Aronsson G, Leineweber C, *et al.* Sickness Presenteeism Predicts Suboptimal Self-Rated Health and Sickness Absence: A Nationally Representative Study of the Swedish Working Population. *PLOS ONE* 2012;**7**:e44721. doi:10.1371/journal.pone.0044721

42 The serotonin theory of depression: a systematic umbrella review of the evidence | Molecular Psychiatry. https://www.nature.com/articles/s41380-022-01661-0 (accessed 4 Oct 2022).

43 Ludvigsson JF, Svedberg P, Olén O, *et al.* The longitudinal integrated database for health insurance and labour market studies (LISA) and its use in medical research. *Eur J Epidemiol* 2019;**34**:423–37. doi:10.1007/s10654-019-00511-8

For peer review only

Figure 1. Flow chart of the total study population.

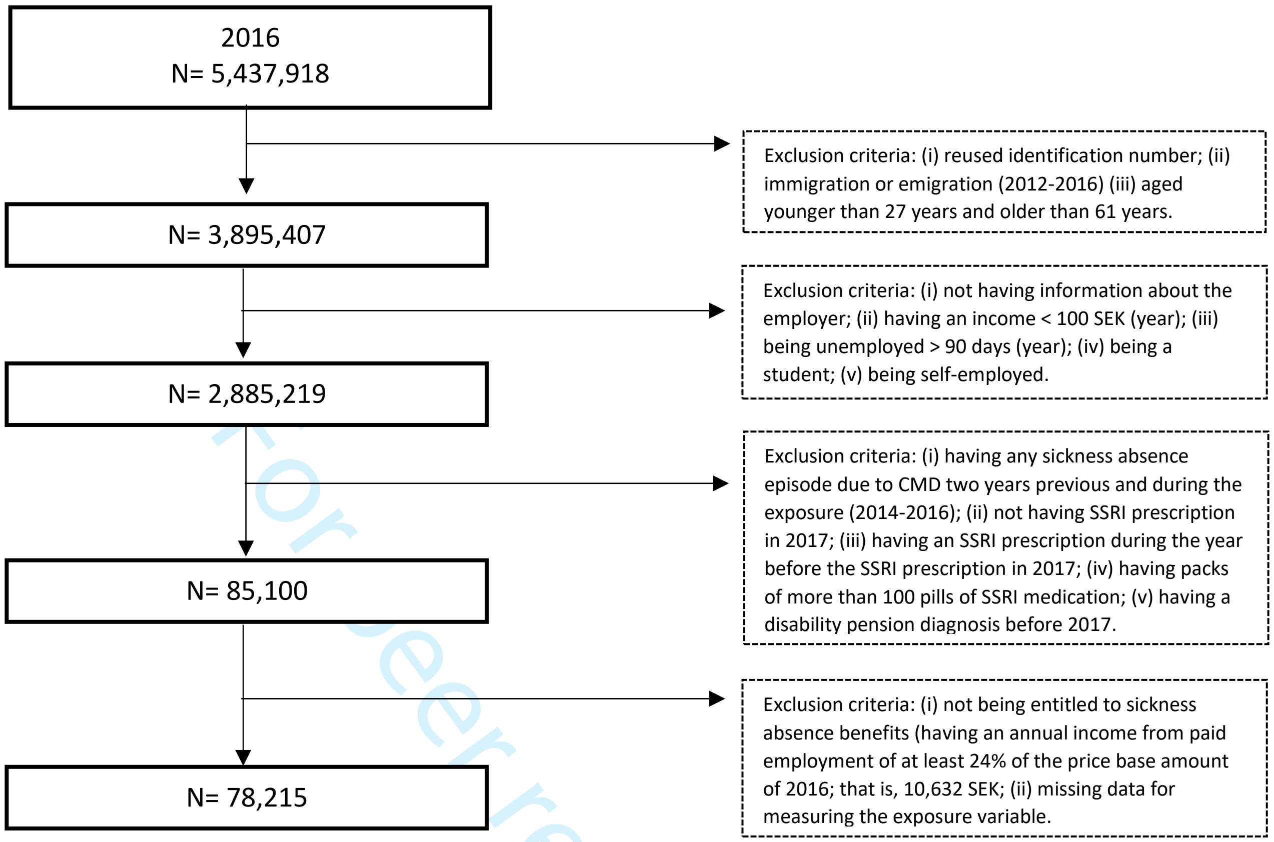

For peer review only

For peer review only - http://bmjopen.bmj.com/site/about/guidelines.xhtml

Figure 2. Directed Acyclic Graph (DAG) of the relation between employment quality typologies (EQT) and sickness absence benefits (SABs) due to common mental disorders (CMD).

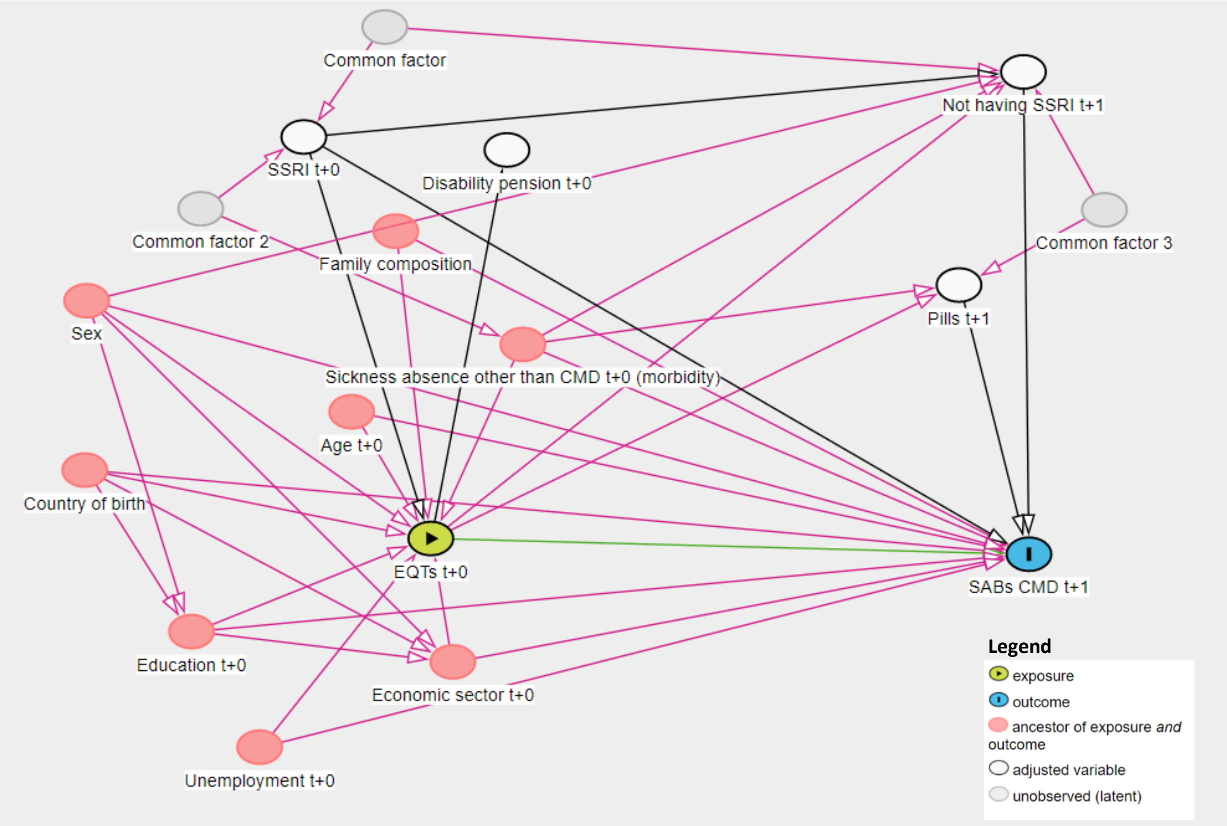

Minimal sufficient adjustment sets for estimating the direct effect of EQTs t+0 (baseline, 2016) on SABs due to CMD t+1 is age, gender, education, country of birth, family composition, economic sector, unemployment, and sickness absence due to diagnoses other than CMD, not having disability pension before 2017, having Selective Serotonin Reuptake Inhibitors (SSRI) prescriptions close to the SABs in 2017, not having SSRI prescription during the year before the SSRI prescription in 2017 and not having packs of more than 100 pills in 2017.

For peer review only - http://bmjopen.bmj.com/site/about/guidelines.xhtml

Supplementary Table 1. Sociodemographic and employment-related characteristics at baseline (2016) of the Swedish working population and the study population with common mental disorders.

	Population*	Study population**
	N (%)	N (%)
**Age**		
27-40 years	1,044,971 (37.4)	30,675 (39.2)
41-51 years	976,697 (34.9)	28,541 (36.5)
52-61 years	774,838 (27.7)	18,999 (24.3)
**Gender**		
Male	1,412,310 (50.5)	25,894 (33.1)
Female	1,384,196 (49.5)	52,321 (66.9)
**Education level**		
Elementary/high school education	1,518,999 (54.4)	41,072 (52.5)
Higher education <3 years	429,648 (15.4)	11,456 (14.7)
Higher education ≥3 years	845,355 (30.3)	25,651 (32.8)
**Country of birth**		
Sweden	2,459,096 (88.0)	69,736 (89.2)
Not Sweden	335,518 (12.0)	8,438 (10.8)
**Family composition**		
Couple with children	1,400,506 (50.1)	37,471 (47.9)
Couple without children	352,101 (12.6)	8,421 (10.8)
Single with children	260,276 (9.3)	10,243 (13.1)
Single without children	783,622 (28.0)	22,080 (28.2)
**Unemployment**		
0 days unemployed	2,718,662 (97.2)	76,090 (97.3)
From 1 to 90 days unemployed	77,844 (2.8)	2,125 (2.7)
**Economic sector**		
Private	1,741,419 (62.3)	41,653 (53.3)
Public	1,055,087 (37.7)	36,562 (46.7)
TOTAL	2,796,506 (100.0)	78,215 (100.0)

*The entire study population meeting the inclusion and exclusion criteria (except having SSRI prescriptions in 2017); **The final study population meeting the whole inclusion and exclusion criteria. Missing values in educational level (population: 2,504, 0.09%; study population: 41, 0.05%), country of birth (population: 1892, 0.07%; study population: 41, 0.05%) and family composition (population: 1, <0.01%).

For peer review only

Supplementary Table 2. Incidence of a first sickness absence episode due to common mental disorders in a cohort of 78,215 individuals with common mental disorders, aged 27-61, entitled to sickness absence benefits in 2016, by sociodemographic and employment characteristics at baseline (2016)

	Sickness absence benefits (7.48%)
	N (%)
**Age**	
27-40 years	2,808 (9.2)
41-51 years	1,901 (6.7)
52-61 years	1,139 (6.0)
**Gender**	
Male	2,136 (8.2)
Female	3,712 (7.1)
**Education level**	
Elementary/high school education	3,502 (8.5)
Higher education <3 years	793 (6.9)
Higher education ≥3 years	1,547 (6.0)
**Country of birth**	
Sweden	5,058 (7.3)
Not Sweden	783 (9.3)
**Family composition**	
Couple with children	2,678 (7.1)
Couple with no children	519 (6.2)
Single with children	808 (7.9)
Single	1,843 (8.3)
**Unemployment**	
0 days unemployed	5,697 (7.5)
From 1 to 90 days unemployed	151 (7.1)
**Economic sector**	
Private	3218 (7.7)
Public	2630 (7.2)
**Employment quality typology**	
Standard Employment Relationship/HI	593 (5.2)
Standard Employment Relationship	2,268 (7.6)
Sub-standard Employment Relationship	2,667 (8.1)
Precarious Employment Relationship	320 (8.0)
**Contractual relationship insecurity**	
Directly employed by an employer	5,788 (7.5)
Employed by an agency	60 (8.2)
**Contractual temporariness**	
Stable employment	4,351 (7.4)
Unstable employment	1,497 (7.8)
**Multiple jobs/economic sectors**	
1-2 jobs	5,436 (7.4)
3 or more jobs	279 (7.8)
3 or more jobs in 3 or more sectors	133 (8.2)
**Income level***	
<60% of the median	395 (7.6)
60-79% of the median	1,415 (8.9)
80-119% of the median	3,113 (7.7)
120-200% of the median	831 (5.7)
>200% of the median	94 (4.0)
**Unionization level**	
<70 %	124 (7.0)
70-90%	315 (6.8)
>90%	5,409 (7.5)

Missing values in the educational level (36, 0.05%) and country of birth (41, 0.05%). Note: Persons entitled to sickness absence benefits in 2016 are those having an annual income from paid employment of at least 24% of the price base amount of 2016; that is, 10,632 SEK. *Income level categories based on the median of the Swedish working population (375,300 SEK) after applying several inclusion and exclusion criteria (see methods section). Income category boundaries: <60% of the median (225,180 SEK), 60-79% of the median (225,180-296,487 SEK), 80-119% of the median (300,240-446,607 SEK), 120-199% of the median (450,360-746,847 SEK), and ≥200% of the median (750,600 SEK).

For peer review only

Supplementary Table 3. Incidence of a first sickness absence episode due to common mental disorders in women and men in a cohort of 85,164 individuals with common mental disorders, aged 27-61, entitled to sickness absence benefits in 2016, by sociodemographic and employment characteristics at baseline (2016).

	Sickness absence benefits	
	**Women (7.1%)**	**Men (8.2%)**
	N (%)	N (%)
**Age**		
27-40 years	1782 (8.7)	1026 (10.2)
41-51 years	1204 (6.3)	697 (7.4)
52-61 years	726 (5.8)	413 (6.4)
**Education level**		
Elementary/high school education	2016 (8.0)	1486 (9.3)
Higher education <3 years	484 (6.3)	309 (8.2)
Higher education ≥3 years	1208 (6.2)	339 (5.5)
**Country of birth**		
Sweden	3195 (6.9)	1863 (8.0)
Not Sweden	512 (8.8)	271 (10.4)
**Family composition**		
Couple with children	1735 (6.8)	943 (7.8)
Couple without children	357 (6.2)	162 (6.2)
Single with children	647 (7.6)	161 (9.2)
Single without children	973 (7.7)	870 (9.2)
**Unemployment**		
0 days unemployed	3632 (7.1)	2065 (8.2)
From 1 to 90 days unemployed	80 (6.0)	71 (8.9)
**Economic sector**		
Private	1,594 (7.0)	1,624 (8.5)
Public	2,118 (7.1)	512 (7.4)
**Employment quality typology**		
Standard Employment Relationship/HI	247 (4.5)	346 (5.8)
Standard Employment Relationship	1375 (7.0)	893 (8.8)
Sub-standard Employment Relationship	1892 (7.7)	775 (9.1)
Precarious Employment Relationship	198 (7.1)	122 (10.1)
**Contractual relationship insecurity**		
Directly employed by an employer	3683 (7.1)	2105 (8.2)
Employed by an agency	29 (6.5)	31 (10.9)
**Contractual temporariness**		
Stable employment	2792 (7.0)	1559 (8.0)
Unstable employment	920 (7.2)	577 (9.0)
**Multiple jobs/economic sectors**		
1-2 jobs	3463 (7.1)	1973 (8.2)
3 or more jobs	175 (7.0)	104 (9.6)
3 or more jobs in 3 or more sectors	74 (7.3)	59 (9.6)
**Income level**		
<60% of the median	319 (7.3)	76 (8.9)
60-79% of the median	1111 (8.4)	304 (10.9)
80-119% of the median	1876 (7.1)	1237 (8.9)
120-200% of the median	378 (5.1)	453 (6.4)
>200% of the median	28 (2.9)	66 (4.9)
**Unionization level**		
<70 %	54 (5.8)	70 (8.2)
70-90%	161 (6.1)	154 (7.7)
>90%	3497 (7.2)	1912 (8.3)

Missing values in education level (women: 19, 0.04%; men: 17, 0.07%) and country of birth (women: 19, 0.04%; men: 22, 0.08%). Note: Persons entitled to sickness absence benefits in 2016 are those having an annual income from paid employment of at least 24% of the price base amount of 2016; that is, 10,632 SEK. *Income level categories based on the median of the Swedish working population (375,300 SEK) after applying several inclusion and exclusion criteria (see methods section). Income category boundaries: <60% of the median (225,180 SEK), 60-79% of the median (225,180-296,487 SEK), 80-119% of the median (300,240-446,607 SEK), 120-199% of the median (450,360-746,847 SEK), and ≥200% of the median (750,600 SEK).

For peer review only

Supplementary Table 4. Incidence of a first sickness absence episode due to common mental disorders in a cohort of 85,164 individuals with common mental disorders, aged 27-61, entitled to sickness absence benefits in 2016, by employment quality typology (Standard Employment Relationship, SER; Standard Employment Relationship with high income, SER/HI; Sub-standard Employment Relationship, SSER; Precarious Employment Relationship, PER; baseline, 2016).

	Sickness absence benefits			
	SER/HI (5.2%)	SER (7.6%)	SSER (8.1%)	PER (8%)
	N (%)	N (%)	N (%)	N (%)
**Age**				
27-40 years	180 (6.3)	938 (9.6)	1481 (9.5)	209 (8.8)
41-51 years	253 (4.8)	794 (6.9)	773 (7.1)	81 (7.9)
52-61 years	160 (4.8)	536 (6.3)	413 (6.3)	30 (5.1)
**Gender**				
Male	346 (5.8)	893 (8.8)	775 (9.1)	122 (10.1)
Female	247 (4.5)	1375 (7.0)	1892 (7.7)	198 (7.1)
**Education level**				
Elementary/high school education	209 (6.2)	1340 (8.5)	1731 (9.0)	222 (8.8)
Higher education <3 years	116 (5.8)	321 (7.3)	311 (7.0)	45 (7.1)
Higher education ≥3 years	268 (4.4)	606 (6.4)	620 (6.7)	53 (6.4)
**Country of birth**				
Sweden	527 (5.0)	1962 (7.4)	2282 (7.8)	287 (8.1)
Not Sweden	66 (6.4)	305 (9.6)	379 (10.1)	33 (7.1)
**Family composition**				
Couple with children	288 (4.8)	971 (7.3)	1286 (7.9)	133 (7.5)
Couple without children	65 (4.8)	241 (6.6)	202 (6.4)	11 (3.8)
Single with children	59 (4.7)	304 (7.8)	396 (8.7)	49 (8.8)
Single without children	181 (6.4)	752 (8.4)	783 (8.7)	127 (9.3)
**Unemployment**				
0 days unemployed	590 (5.2)	2253 (7.6)	2589 (8.2)	265 (7.8)
From 1 to 90 days unemployed	3 (5.6)	15 (6.3)	78 (6.2)	55 (9.6)
**Economic sector**				
Private	384 (5.5)	1,186 (8.5)	1,383 (7.9)	265 (8.3)
Public	209 (4.7)	1,082 (6.8)	1,284 (8.3)	55 (6.8)
**Contractual relationship insecurity**				
Directly employed by an employer	593 (5.2)	2263 (7.6)	2636 (8.1)	296 (7.9)
Employed by an agency	0	5 (13.2)	31 (6.7)	24 (10.9)
**Contractual temporariness**				
Stable employment	593 (5.2)	2246 (7.7)	1503 (8.3)	9 (3.5)
Unstable employment	0	22 (4.7)	1164 (7.8)	311 (8.3)
**Multiple jobs/economic sectors**				
1-2 jobs	579 (5.2)	2220 (7.6)	2479 (8.1)	158 (7.3)
3 or more jobs	11 (5.0)	36 (8.2)	155 (8.1)	77 (7.7)
3 or more jobs in 3 or more sectors	3 (2.5)	12 (6.3)	33 (6.7)	85 (10.3)
**Income level**				
<60% of the median	0	0	270 (8.1)	125 (6.6)
60-79% of the median	0	0	1313 (8.9)	102 (8.8)
80-119% of the median	0	2203 (7.7)	822 (7.7)	88 (10.0)
120-200% of the median	536 (5.4)	42 (7.1)	249 (6.3)	4 (6.5)
>200% of the median	57 (3.5)	23 (4.5)	13 (7.1)	1 (100.0)
**Unionization level**				
<70%	0	1 (5.0)	58 (6.8)	65 (7.2)
70-90%	2 (3.6)	26 (6.3)	223 (6.8)	64 (7.4)
>90%	591 (5.2)	2241 (7.6)	2386 (8.3)	191 (8.6)

Missing values in education level (SER/HI: 7, 0.06%; SER: 10, 0.03%; SSER: 17, 0.05%; PE: 2, 0.05%) and country of birth (SER/HI: 3, 0.03%; SER: 18, 0.06%; SSER: 20, 0.06%). Note: Persons entitled to sickness absence benefits in 2016 are those having an annual income from paid employment of at least 24% of the price base amount of 2016; that is, 10,632 SEK. *Income level categories based on the median of the Swedish working population (375,300 SEK) after applying several inclusion and exclusion criteria (see methods section). Income category boundaries: <60% of the median (225,180 SEK), 60-79% of the median (225,180-296,487 SEK), 80-119% of the median (300,240-446,607 SEK), 120-199% of the median (450,360-746,847 SEK), and ≥200% of the median (750,600 SEK).

For peer review only - http://bmjopen.bmj.com/site/about/guidelines.xhtml

STROBE Statement—checklist of items that should be included in reports of observational studies

	Item No.	Recommendation	Page No.	Relevant text from manuscript
**Title and abstract**	1	(*a*) Indicate the study's design with a commonly used term in the title or the abstract	2	
		(*b*) Provide in the abstract an informative and balanced summary of what was done and what was found	2	
**Introduction**				
Background/rationale	2	Explain the scientific background and rationale for the investigation being reported	4	
Objectives	3	State specific objectives, including any prespecified hypotheses	4	
**Methods**				
Study design	4	Present key elements of study design early in the paper	5	
Setting	5	Describe the setting, locations, and relevant dates, including periods of recruitment, exposure, follow-up, and data collection	5	
Participants	6	(*a*) *Cohort study*—Give the eligibility criteria, and the sources and methods of selection of participants. Describe methods of follow-up<br>*Case-control study*—Give the eligibility criteria, and the sources and methods of case ascertainment and control selection. Give the rationale for the choice of cases and controls<br>*Cross-sectional study*—Give the eligibility criteria, and the sources and methods of selection of participants	5	
		(*b*) *Cohort study*—For matched studies, give matching criteria and number of exposed and unexposed<br>*Case-control study*—For matched studies, give matching criteria and the number of controls per case		
Variables	7	Clearly define all outcomes, exposures, predictors, potential confounders, and effect modifiers. Give diagnostic criteria, if applicable	5,6	
Data sources/ measurement	8*	For each variable of interest, give sources of data and details of methods of assessment (measurement). Describe comparability of assessment methods if there is more than one group	5-6	
Bias	9	Describe any efforts to address potential sources of bias	2 (fig. file)	Figure 2
Study size	10	Explain how the study size was arrived at	1 (fig. file)	Figure 1
Continued on next	11	Explain how quantitative variables were handled in the analyses. If applicable, describe which	6	

For peer review only - http://bmjopen.bmj.com/site/about/guidelines.xhtml

page		groupings were chosen and why		
Quantitative variables				
Statistical methods	12	(*a*) Describe all statistical methods, including those used to control for confounding	6	
		(*b*) Describe any methods used to examine subgroups and interactions	6	
		(*c*) Explain how missing data were addressed	N/A	
		(*d*) *Cohort study*—If applicable, explain how loss to follow-up was addressed<br>*Case-control study*—If applicable, explain how matching of cases and controls was addressed<br>*Cross-sectional study*—If applicable, describe analytical methods taking account of sampling strategy	N/A	
		(*e*) Describe any sensitivity analyses	6	
**Results**				
Participants	13*	(a) Report numbers of individuals at each stage of study—eg numbers potentially eligible, examined for eligibility, confirmed eligible, included in the study, completing follow-up, and analysed	1 (fig and supp files)	Figure 1 and Suppl. Table 1
		(b) Give reasons for non-participation at each stage	1 (fig file)	Figure 1
		(c) Consider use of a flow diagram	1 (fig file)	Figure 1
Descriptive data	14*	(a) Give characteristics of study participants (eg demographic, clinical, social) and information on exposures and potential confounders	8	Table 1
		(b) Indicate number of participants with missing data for each variable of interest	8 and 1-4 (supp file)	Footnote Table 1 and Suppl. Tables 1-4 (supp file)
		(c) *Cohort study*—Summarise follow-up time (eg, average and total amount)	N/A	
Outcome data	15*	*Cohort study*—Report numbers of outcome events or summary measures over time	9,11	Tables 2 and 3
		Case-control study—Report numbers in each exposure category, or summary measures of exposure		
		Cross-sectional study—Report numbers of outcome events or summary measures		
Main results	16	(*a*) Give unadjusted estimates and, if applicable, confounder-adjusted estimates and their precision (eg, 95% confidence interval). Make clear which confounders were adjusted for and why they were included	9,11	Tables 2 and 3
		(*b*) Report category boundaries when continuous variables were categorized	8,11 and 2-4 (supp file)	Tables 1,3 and Suppl. Tables 2-4 (supp file)
		(*c*) If relevant, consider translating estimates of relative risk into absolute risk for a meaningful time period	N/A	

For peer review only - http://bmjopen.bmj.com/site/about/guidelines.xhtml
For peer review only

Continued on next page

Other analyses	17	Report other analyses done—eg analyses of subgroups and interactions, and sensitivity analyses	N/A	
**Discussion**				
Key results	18	Summarise key results with reference to study objectives	12	First paragraph of the discussion
Limitations	19	Discuss limitations of the study, taking into account sources of potential bias or imprecision. Discuss both direction and magnitude of any potential bias	13	
Interpretation	20	Give a cautious overall interpretation of results considering objectives, limitations, multiplicity of analyses, results from similar studies, and other relevant evidence	12-13	
Generalisability	21	Discuss the generalisability (external validity) of the study results	14	
**Other information**				
Funding	22	Give the source of funding and the role of the funders for the present study and, if applicable, for the original study on which the present article is based	14	

*Give information separately for cases and controls in case-control studies and, if applicable, for exposed and unexposed groups in cohort and cross-sectional studies.

Note: An Explanation and Elaboration article discusses each checklist item and gives methodological background and published examples of transparent reporting. The STROBE checklist is best used in conjunction with this article (freely available on the Web sites of PLoS Medicine at http://www.plosmedicine.org/, Annals of Internal Medicine at http://www.annals.org/, and Epidemiology at http://www.epidem.com/). Information on the STROBE Initiative is available at www.strobe-statement.org.

For peer review only

For peer review only - http://bmjopen.bmj.com/site/about/guidelines.xhtml

BMJ Open

Unequal access? Use of sickness absence benefits by precariously employed workers with common mental disorders: A register-based cohort study in Sweden

Journal:	*BMJ Open*
Manuscript ID	bmjopen-2023-072459.R1
Article Type:	Original research
Date Submitted by the Author:	31-May-2023
Complete List of Authors:	Hernando-Rodriguez, Julio C.; Karolinska Institutet, Unit of Occupational and Environmental Medicine, Institute of Environmental Medicine<br>Matilla-Santander, Nuria; Karolinska Institutet, Unit of Occupational and Environmental Medicine, Institute of Environmental Medicine<br>Murley, Chantelle; Karolinska Institutet, Division of Insurance Medicine, Department of Clinical Neuroscience<br>Blindow, Katrina; Karolinska Institutet, Unit of Occupational and Environmental Medicine, Institute of Environmental Medicine<br>Kvart, Signild; Karolinska Institutet, Unit of Occupational and Environmental Medicine, Institute of Environmental Medicine<br>Almroth, Melody; Karolinska Institutet, Unit of Occupational and Environmental Medicine, Institute of Environmental Medicine; Stockholm University, Department of Public Health Sciences<br>Kreshpaj, Bertina; Karolinska Institutet, Unit of Occupational and Environmental Medicine, Institute of Environmental Medicine; University of Copenhagen, Section of Epidemiology, Department of Publich Health<br>Thern, Emelie; Karolinska Institute, Unit of Occupational Medicine, Institute of Environmental Medicine<br>Badarin, Kathryn; Karolinska Institutet, Unit of Occupational Medicine, Institute of Environmental Medicine<br>Muntaner, Carles; University of Toronto, Lawrence S. Bloomberg Faculty of Nursing; University of Toronto, Dalla Lana School of Public Health<br>Gunn, Virginia; Karolinska Institutet, Unit of Occupational and Environmental Medicine, Institute of Environmental Medicine; St Michael's Hospital Centre for Urban Health Solutions, Li Ka Shing Knowledge Institute, Unity Health Toronto<br>Padrosa, Eva; Consorci Parc de Salut MAR de Barcelona<br>Julià, Mireia; Consorci Parc de Salut MAR de Barcelona<br>Bodin, Theo; Karolinska Institutet, Unit of Occupational and Environmental Medicine, Institute of Environmental Medicine; Centre for Occupational and Environmental Medicine, Stockholm Region
**Primary Subject Heading**:	Occupational and environmental medicine
Secondary Subject Heading:	Public health, Epidemiology
Keywords:	MENTAL HEALTH, EPIDEMIOLOGY, OCCUPATIONAL & INDUSTRIAL MEDICINE, PUBLIC HEALTH

SCHOLARONE™
Manuscripts

I, the Submitting Author has the right to grant and does grant on behalf of all authors of the Work (as defined in the below author licence), an exclusive licence and/or a non-exclusive licence for contributions from authors who are: i) UK Crown employees; ii) where BMJ has agreed a CC-BY licence shall apply, and/or iii) in accordance with the terms applicable for US Federal Government officers or employees acting as part of their official duties; on a worldwide, perpetual, irrevocable, royalty-free basis to BMJ Publishing Group Ltd ("BMJ") its licensees and where the relevant Journal is co-owned by BMJ to the co-owners of the Journal, to publish the Work in this journal and any other BMJ products and to exploit all rights, as set out in our licence.

The Submitting Author accepts and understands that any supply made under these terms is made by BMJ to the Submitting Author unless you are acting as an employee on behalf of your employer or a postgraduate student of an affiliated institution which is paying any applicable article publishing charge ("APC") for Open Access articles. Where the Submitting Author wishes to make the Work available on an Open Access basis (and intends to pay the relevant APC), the terms of reuse of such Open Access shall be governed by a Creative Commons licence – details of these licences and which Creative Commons licence will apply to this Work are set out in our licence referred to above.

Other than as permitted in any relevant BMJ Author's Self Archiving Policies, I confirm this Work has not been accepted for publication elsewhere, is not being considered for publication elsewhere and does not duplicate material already published. I confirm all authors consent to publication of this Work and authorise the granting of this licence.

For peer review only

Unequal access? Use of sickness absence benefits by precariously employed workers with common mental disorders: A register-based cohort study in Sweden

Julio C. Hernando-Rodriguez, PhD[1], Nuria Matilla-Santander, PhD[1], Chantelle Murley, PhD[2], Katrina Blindow, MPH[1], Signild Kvart, MSc[1], Melody Almroth, PhD[1,3], Bertina Kreshpaj, PhD[1,4], Emelie Thern, PhD[1], Kathryn Badarin, PhD[1], Carles Muntaner, PhD[5,6,7], Virginia Gunn, PhD[1,8,9], Eva Padrosa, PhD[,10,11,12], Mireia Julià, PhD[10,11,12], Theo Bodin, PhD[1,13]

[1]Unit of Occupational Medicine, Institute of Environmental Medicine, Karolinska Institutet, Stockholm, Sweden; [2]Division of Insurance Medicine, Department of Clinical Neuroscience, Karolinska Institutet; Stockholm, Sweden; [3]Department of Public Health Sciences, Stockholm University; [4]Section of Epidemiology, Department of Public Health, University of Copenhagen, Copenhagen, Denmark; [5]Lawrence S. Bloomberg Faculty of Nursing, St. George Campus, Toronto, Canada; [6]Dalla Lana School of Public Health, University of Toronto, Toronto, Canada; [7]Department of Mental Health, The Johns Hopkins University Bloomberg School of Public Health, Baltimore, USA; [8]MAP Centre for Urban Health Solutions, Li Ka Shing Knowledge Institute, Unity Health Toronto, Toronto, Canada; [9]School of Nursing, Cape Breton University, Nova Scotia, Canada; [10]ESIMar (Mar Nursing School), Parc de Salut Mar, Universitat Pompeu Fabra-affiliated, Barcelona, Spain; [11]SDHEd (Social Determinants and Health Education Research Group), IMIM (Hospital del Mar Medical Research Institute), Barcelona, Spain; [12]GREDS (Research Group on Health Inequalities, Environment, and Employment Conditions Network), Universitat Pompeu Fabra, Barcelona, Spain; [13]Centre for Occupational and Environmental Medicine, Stockholm Region, Stockholm, Sweden

Corresponding author: Unit of Occupational Medicine, The Institute of Environmental Medicine (IMM), Karolinska Institutet, Solnavägen 4, 113 65, Stockholm, Sweden. julio.hernando@ki.se

Word count text (from introduction to conclusion): 3,814.

Number of tables: 3 (also 5 tables in the supplementary material in another file).

Number of figures: 2 (in another file).

For peer review only

For peer review only - http://bmjopen.bmj.com/site/about/guidelines.xhtml

ABSTRACT

Unequal access? Use of sickness absence benefits by precariously employed workers with common mental disorders: A register-based cohort study in Sweden

Objective

This study compares the use of sickness absence benefits (SABs) due to a common mental disorder (CMD) between precarious and non-precarious workers with CMD.

Design

Register-based cohort study.

Participants

The study included 78,215 Swedish workers aged 27-61 who experienced CMD in 2017, indicated by a new treatment with Selective Serotonin Reuptake Inhibitors (SSRI). Excluded were those who emigrated or immigrated, were self-employed, had an annual employment-based income < 100 Swedish krona, had > 90 days of unemployment per year, had student status, had SABs due to CMD during the exposure measurement (2016) and the two previous years, had an SSRI prescription one year or less before the start of the SSRI prescription in 2017, had packs of > 100 pills of SSRI medication, had a disability pension before 2017, were not entitled to SABs due to CMD in 2016, and had no information about the exposure.

Outcome

The first incidence of SABs due to CMD in 2017.

Results

The use of SABs due to a CMD was slightly lower among precariously employed workers compared to those in standard employment [adjusted Odds Ratio (aOR) 0.92, 95% confidence interval (CI) 0.81-1.05]. Particularly, women with three consecutive years in precarious employment had reduced SABs use [aOR 0.48, 95% CI 0.26-0.89], while men in precarious employment showed weaker evidence of association. Those in standard employment with high income also showed a lower use of SABs [aOR 0.74, 95% CI 0.67-0.81]. Low unionization and both low and high-income levels were associated with lower use of SABs, particularly among women.

Conclusions

The study indicates that workers with CMD in precarious employment may use SABs to a lower extent. Accordingly, there is a need for (i) guaranteeing access to SABs for people in precarious employment and/or (ii) reducing involuntary forms of presenteeism.

Key terms: employment quality; precarious employment; SSRI; anxiety; depression; stress-related disorders; mental disorders; mental health; sick leave; welfare state; social insurance system; labour market; epidemiology; Sweden; longitudinal study; presenteeism.

For peer review only - http://bmjopen.bmj.com/site/about/guidelines.xhtml

Strengths and limitations of this study:

- This study utilizes population registers with high validity and low attrition rates.

- The linkage of work disability and pharmaceutical registers enables accurate measurement of sickness absence in individuals in formal employment receiving new CMD treatment.

- The longitudinal design of this study minimizes the potential for reverse causation.

- Potential underestimation of findings by requiring three years of exposure to precarious employment (those with longer exposure tend to be healthier) is mitigated in the one-year analysis.

For peer review only

For peer review only - http://bmjopen.bmj.com/site/about/guidelines.xhtml

BACKGROUND

In the past decades, many countries, including Sweden, have experienced changes in labour market policies that prioritize organizational flexibility over employment security [1]. This led to an increase in precarious employment (PE) [2], which is characterized by employment insecurity, insufficient income, and a lack of rights and social protection [3,4]. The impact of these labour market changes on the health of the workforce and the use of sickness absence benefits (SABs) is not fully understood, especially, among workers in PE.

Mental disorders are one of the major causes of the global burden of disease [5]. In Sweden, half of the working-age population experiences a mental health condition during their lifetime; every year, 1 million workers have a mental disorder [6]. As a result, mental disorders are one of the leading diagnoses causing sickness absence (SA), especially among young people [7]. SA rates are not only driven by the health and work capacity of the working population but are influenced by contextual factors. Therefore, it has been suggested that low rates of SA in insecure jobs may reflect higher levels of presenteeism (i.e., working while being ill) due to workers' fear of dismissal rather than better health status [8,9].

In Sweden, the proportion of the working-age population who are at risk of not being entitled to SA is among the lowest (less than 1%) in the European Union [10]. Those who are not entitled to SA are often family workers employed in small family businesses and who do not receive a formal salary and, therefore, do not pay social contributions [10]. However, in 2016, the Swedish Social Insurance Inspectorate (ISF) deemed temporary employees' access to SABs to be legally uncertain, mainly due to the design of regulations that leave room for interpretation [11]. Workers without fixed schedules are particularly affected because this causes ambiguity as to what hours they would have worked had they not been sick, which opens the possibility to consider non-working periods as unemployment. The regulations of SABs are different for employees and unemployed persons, including entitlement requirements and the level of compensation. Moreover, the ISF found that temporary employees had fewer days of SABs than those permanently employed.

Previous studies have examined demographic characteristics such as age and gender, singular employment characteristics, trade union membership or work environment factors in relation to SA [12–15]. Some of these determinants of SA have been examined specifically among people with common mental disorders (CMD – stress-related disorders, depression, and anxiety) [16]. To our knowledge, only two previous cross-sectional studies have analysed the relationship between PE and SA, and both of which found a positive association [17,18]. This is in line with previous studies confirming a relationship between PE and poor health, including a systematic review of longitudinal studies [19]. A recent Swedish study using the same data sources as the present study found that trajectories with constant PE increased the risk of CMD [20].

Accordingly, we hypothesise that workers in PE would have a higher likelihood of using SABs due to CMD when compared to those in standard employment due to their worse mental health status. However, workers in PE who formally qualify for SABs may still experience barriers to accessing their rights. To investigate the use of SABs, we include only individuals who have recently initiated treatment for CMD and who formally qualify for SABs.

This study aims to compare the use of SABs due to CMD between precariously employed and non-precariously employed workers with CMD.

For peer review only - http://bmjopen.bmj.com/site/about/guidelines.xhtml

METHODS

Study population

This register-based study is based on the Swedish Work, Illness, and Labour-market Participation (SWIP) cohort of the total Swedish population. The SWIP cohort is obtained through the linkage of multiple registers and includes all individuals aged 16-65 years who were registered in Sweden in 2005 (around 5.7 million individuals), with follow-up until 2017. This study uses a subpopulation of 78,215 individuals aged 27-61 years in 2016 who experienced a CMD in 2017, indicated by the initiation of treatment with Selective Serotonin Reuptake Inhibitors (SSRIs). Included individuals had similar characteristics to the Swedish workforce, except for the higher share of women (Supplementary Table 1).

Sociodemographic and employment data were obtained from the Longitudinal Integration Database for Health Insurance and Labour Market Studies (LISA) for 2016. Data on SA (from day 15 onwards) were taken from the Micro Data for Analysis of Social Insurance (MiDAS) registers and included information such as ICD-10 diagnosis and length of SA for 2017. The Swedish Prescribed Drug Register informed the prescribed medicines dispensed from pharmacies for 2017.

Exclusion criteria were: (i) emigration or immigration (2012-2016), (ii) self-employment during 2014-2017 because SABs regulations differ [21], (iii) annual employment-based income < 100 SEK to ensure working status, (iv) > 90 days of unemployment per year to avoid measuring the effect of unemployment on the outcome, (v) student status during the autumn term to avoid misclassification as PE, (vi) SA due to CMD during the exposure measurement (2016) and the two previous years (2014-2015) to reduce the risk of reverse causation, (vii) SSRI prescription one year or less before the start of SSRI prescription in 2017 [22,23], (viii) packs of > 100 pills of SSRI medication in 2017, as this is uncommon for new SSRI treatment, (ix) disability pension before 2017, (x) no entitlement to SABs due to CMD in 2016, and (xi) incomplete information on the exposure variable in 2016 (2014-2016 in the sensitivity analysis) (Figure 1).

Exposure variable

The PE measure was based on the second version of the Swedish Register-based Operationalization of Precarious Employment (SWE-ROPE) [24]. The SWE-ROPE considers five items (i.e., contractual relationship insecurity, contractual temporariness, multiple jobs/economic sectors, income level and lack of unionization) that represent the three dimensions of PE (i.e., employment insecurity, income inadequacy and lack of rights and protection). For each individual, an annual score for each of the five items was calculated [25]. Scores ranged by item: contractual employment insecurity (agency employed to directly employed: from -1 and 0); contractual temporariness (unstable employment and stable employment: -2 and 0); multiple jobs/economic sectors (multiple jobs and sectors, multiple jobs, a single job: -2 to 0), income level (< 60% of the median, 60-79%, 80-119%, 120-200% and > 200%: -2 to +2) and lack of unionization (< 70% of collective bargaining agreement coverage, 70-90% and > 90%: -2 to 0). The median income (375,300 SEK in 2016) was based on the Swedish working population after applying the inclusion and some of the main exclusion criteria (i to vii and xi described above).

A final summative score was obtained by summing the total score of the five items. Lower final scores suggest PE, while a higher score suggests non-PE. The final summative score for 2016 was categorised into four employment quality typologies: precarious employment relationship (PER;

For peer review only - http://bmjopen.bmj.com/site/about/guidelines.xhtml

score<-3), sub-standard employment relationship (SSER; a score between -3 and -1), standard employment relationship (SER; score=0) and standard employment relationship with high income (SER/HI; score>0). The SER/HI group was defined in light of previous literature [20,26] and included more than 85% of individuals with high-income levels (120-199% of the median income).

Outcome variable

The outcome was the first occurrence of SABs due to CMD starting in the 2 months spanning the month before and after the SSRI prescription date in 2017 (categorical outcome: 0, having SSRI and no SABs; 1, having SSRI co-occurring with SABs). This period was selected based on literature observing the effect of SSRI treatment after 3-4 weeks, so the risk of experiencing a new SA spell after that time may decrease [27–29]. The SSRI prescriptions included antidepressants from N06AB02 to N06AB10 according to the Anatomical Therapeutic Chemical (ATC) classification system. SA due to CMD was identified by ICD-10 codes: depression (F32-F33), anxiety (F41) and stress-related disorders (F43) including exhaustion disorder, which is conceptually similar to burnout. This definition of CMD is based on a previous paper about PE and the risk of CMD [20]. Regarding the SABs, all people living in Sweden aged 16 and over who receive a minimum annual income from work of at least 24% of the price base amount (10,752 SEK in 2017) and who have a reduced work capacity of at least 25% of their usual working hours due to disease or injury are covered by the SA insurance system [30]. A physician's certificate is required after the first seven days of SA. SABs replace up to 80% of lost work income. After the first qualifying day without compensation, the employer pays SA for the first 13 days. Therefore, SA spells of less than 15 days are not registered in MiDAS. From the 15th day onwards, SABs are paid by the Social Insurance Agency. For the present study, SABs due to CMD included only SA spells of at least 15 days.

Potential confounding variables

At baseline, age, education level, country of birth, family composition (civil status and having children), unemployment duration, economic sector, area of residence, occupation, and comorbidity (2016). Comorbidity was measured as having SA due to any diagnoses other than CMD since SA episodes in the previous years showed to be a predictor for future episodes of SA [31].

Statistical analysis

Logistic regression models estimated the association between employment quality typologies and the use of SABs due to CMD in 2017. A sensitivity analysis was conducted by conditioning models to have the same exposure for three consecutive years (2014-2016) to observe if stability could influence the results. Models were adjusted for the potential confounders mentioned above and identified using a Directed Acyclic Graph (DAG) (Figure 2). All analyses were conducted for the total population as well as women and men separately. The analysis is separated by gender because previous studies have found different patterns in SA among women and men [32,33]. Women have higher SA rates compared to men possibly due to the higher exposure to an unequal division of domestic work combined with paid work [32]. Work-family conflicts are related to higher ORs to report short SA (1-7 days) among men and longer SA among women (8-30 days) [33]. Interaction effects of gender and the main exposure were tested. We would like to clarity our approach to interpreting the results of the models by considering confidence intervals as "compatibility intervals" as recommended by Amrhein et al.

For peer review only - http://bmjopen.bmj.com/site/about/guidelines.xhtml

[34]. Rather than solely focusing on statistical significance, we examined the effect sizes that are most compatible with our data.

Data management and statistical analysis were performed using Stata SE v17.

Patient and public involvement

Patients and/or the public were not involved in the design, or conduct, or reporting, or dissemination plans of this research.

RESULTS

People in PER (5.10%) had a higher proportion of young-middle aged individuals (27-40 years) (59.4%), women (69.7%), low educational attainment (63.2%), born in Sweden (88.4%), couples with children (44.4%), zero days unemployed (85.6%), private sector (79.6%), unstable employment (93.5%), multiple job holding in different economic sectors (20.7%), income level below 60% of the median (47.5%), and low unionization (22.6%) (Table 1). The proportion of people having a first spell of SABs due to CMD in 2017 was 7.5% among our study population and showed differences according to sociodemographic and employment characteristics in general (Supplementary Table 2), and by gender and employment quality typology (Supplementary Tables 3 and 4, respectively).

For peer review only - http://bmjopen.bmj.com/site/about/guidelines.xhtml

Table 1. Sociodemographic and employment characteristics at baseline (2016) in a cohort of 78,215 individuals with common mental disorders in 2017, aged 27-61, entitled to sickness absence benefits in 2016 by employment quality typology (Standard Employment Relationship, SER; Standard Employment Relationship with high income, SER/HI; Sub-standard Employment Relationship, SSER; Precarious Employment Relationship, PER; baseline, 2016).

	SER/HI (14.68%)	SER (38.04%)	SSER (42.18%)	PER (5,10%)
	N (%)	N (%)	N (%)	N (%)
**Age**				
27-40 years	2,878 (25.1)	9,817 (33.0)	15,612 (47.3)	2,368 (59.4)
41-51 years	5,239 (45.6)	11,432 (38.4)	10,844 (32.9)	1,026 (25.7)
52-61 years	3,366 (29.3)	8,504 (28.6)	6,537 (19.8)	592 (14.9)
**Gender**				
Male	5,999 (52.2)	10,133 (34.1)	8,554 (25.9)	1,208 (30.3)
Female	5,484 (47.8)	19,620 (65.9)	24,439 (74.1)	2,778 (69.7)
**Education level**				
Elementary/high school education	3,395 (29.6)	15,831 (53.2)	19,329 (58.6)	2,517 (63.2)
Higher education <3 years	1,989 (17.3)	4,384 (14.7)	4,446 (13.5)	637 (16.0)
Higher education ≥3 years	6,092 (53.1)	9,528 (32.0)	9,201 (27.9)	830 (20.8)
**Country of birth**				
Sweden	10,449 (91.0)	26,561 (89.3)	29,203 (88.6)	3,523 (88.4)
Not Sweden	1,031 (9.0)	3,174 (10.7)	3,770 (11.4)	463 (11.6)
**Family composition**				
Couple with children	6,045 (52.6)	13,320 (44.8)	16,338 (49.5)	1,768 (44.4)
Couple without children	1,354 (11.8)	3,630 (12.2)	3,151 (9.6)	286 (7.2)
Single with children	1,252 (10.9)	3,903 (13.1)	4,528 (13.7)	560 (14.0)
Single without children	2,832 (24.7)	8,900 (29.9)	8,976 (27.2)	1,372 (34.4)
**Unemployment**				
0 days unemployed	11,429 (99.5)	29,514 (99.2)	31,733 (96.2)	3,414 (85.6)
From 1 to 90 days unemployed	54 (0.5)	239 (0.8)	1260 (3.8)	572 (14.4)
**Economic sector**				
Private	6,995 (60.9)	13,908 (46.7)	17,578 (53.3)	3,172 (79.6)
Public	4,488 (39.1)	15,845 (53.3)	15,415 (46.7)	814 (20.4)
**Contractual relationship insecurity**				
Directly employed by an employer	11,473 (99.9)	29,715 (99.9)	32,529 (98.6)	3,765 (94.5)
Employed by an agency	10 (0.1)	38 (0.1)	464 (1.4)	221 (5.5)
**Contractual temporariness**				
Stable employment	11,483 (100.0)	29,282 (98.4)	18,070 (54.8)	260 (6.5)
Unstable employment	(0.0)	471 (1.6)	14,923 (45.2)	3,726 (93.5)
**Multiple jobs/economic sectors**				
1-2 jobs	11,143 (97.0)	29,123 (97.9)	30,584 (92.7)	2,168 (54.4)
3 or more jobs	221 (1.9)	440 (1.5)	1,916 (5.8)	994 (24.9)
3 or more jobs in 3 or more sectors	119 (1.0)	190 (0.6)	493 (1.5)	824 (20.7)
**Income level***				
<60% of the median	(0.0)	(0.0)	3,320 (10.1)	1,892 (47.5)
60-79% of the median	(0.0)	(0.0)	14,823 (44.9)	1,153 (28.9)
80-119% of the median	(0.0)	28,657 (96.3)	10,708 (32.5)	878 (22.0)
120-200% of the median	9,849 (85.8)	590 (2.0)	3,960 (12.0)	62 (1.6)
>200% of the median	1,634 (14.2)	506 (1.7)	182 (0.6)	1 (<0.1)
**Unionization level**				
<70%	(0.0)	20 (0.1)	852 (2.6)	901 (22.6)
70-90%	55 (0.5)	412 (1.4)	3,297 (10.0)	866 (21.7)
>90%	11,428 (99.5)	29,321 (98.5)	28,844 (87.4)	2,219 (55.7)

Missing values in the educational level (SER/HI: 7, 0.06%; SER: 10, 0.03%; SSER: 17, 0.05%; PE: 2, 0.05%) and country of birth (SER/HI: 3, 0.03%; SER: 18, 0.06%; SSER: 20, 0.06). Persons entitled to sickness absence benefits in 2016 are those having an annual income from paid employment of at least 24% of the price base amount of 2016; that is, 10,632 SEK. *Income level categories based on the median of the Swedish working population (375,300 SEK) after applying several inclusion and exclusion criteria (see methods section). Income category boundaries: <60% of the median (225,180 SEK), 60-79% of the median (225,180-296,487 SEK), 80-119% of the median (300,240-446,607 SEK), 120-199% of the median (450,360-746,847 SEK), and ≥200% of the median (750,600 SEK).

For peer review only

The association between 1-year exposure to employment quality typologies and the use of SABs due to CMD exhibited an inverted U-shape pattern, with lower adjusted Odds Ratio (aOR) estimates found among individuals with an SER/HI (aOR: 0.74, 95% CI: 0.67-0.81), followed by near-significance estimates of those in a PER, compared to the SER group (aOR: 0.92, 95% CI: 0.81-1.05) (Table 2). This association between PER and SABs suggests 8% reduced risk. However, our data is also compatible with a range of risk differences, spanning from a reduction of 19% to a small increase of 5%. The SSER group showed no significant differences compared to the SER group. Women were less likely to use SABs due to CMD in the PER (OR: 0.88, 95% CI: 0.75-1.04) and the SER/HI groups (aOR: 0.72, 95% CI: 0.63-0.83) compared to the SER group. Men in SSER and PER showed weaker evidence of association, except for those in the SER/HI group (aOR: 0.76, 95% CI: 0.66-0.86). Overall, regression models conditional on having the same exposure for three consecutive years showed similar results, in this case, lower use of SABs due to CMD occurred among individuals with SER/HI (aOR: 0.74, 95% CI: 0.65-0.85). Women with three consecutive years of PER had the lowest probability of using SABs due to CMD (aOR: 0.48, 95% CI: 0.26-0.89), followed by women and men with SER/HI compared to the SER group (aOR: 0.76, 95% CI: 0.62-0.92 and aOR: 0.73, 95% CI: 0.61-0.88, respectively). Given that area of residence and occupation barely changed the estimates, they were not included in the models as confounders.

Table 2. Association between employment quality typologies (Standard Employment Relationship, SER; Standard Employment Relationship with high income, SER/HI; Sub-standard Employment Relationship, SSER; Precarious Employment Relationship, PER; baseline, 2016) and sickness absence benefits due to common mental disorders in 2017 among people entitled to sickness absence benefits in 2016 (N=78,215).

					Models with three years of continuous exposure* (N=38,761)			
	N	Cases N (%)	OR (95% CI)	aOR (95% CI)	N	Cases N (%)	OR (95% CI)	aOR (95% CI)
**Employment quality typology**								
SER	29,753	2,268 (7.6)	1	1	17,353	1,207 (7.0)	1	1
SER/HI	11,483	593 (5.2)	0.66 (0.60-0.72)	0.74 (0.67-0.81)	6,924	330 (4.8)	0.67 (0.59-0.76)	0.74 (0.65-0.85)
SSER	32,993	2,667 (8.1)	1.07 (1.01-1.13)	0.99 (0.93-1.05)	14,065	1,055 (7.5)	1.08 (1.00-1.18)	1.01 (0.92-1.10)
PER	3,986	320 (8.0)	1.06 (0.94-1.20)	0.92 (0.81-1.05)	419	24 (5.7)	0.81 (0.54-1.23)	0.74 (0.48-1.12)
**Women (N=52,321)**								
SER	19,620	1,375 (7.0)	1	1	11,137	715 (6.4)	1	1
SER/HI	5,484	247 (4.5)	0.63 (0.54-0.72)	0.72 (0.63-0.83)	2,954	129 (4.4)	0.67 (0.55-0.81)	0.76 (0.62-0.92)
SSER	24,439	1,892 (7.7)	1.11 (1.04-1.20)	1.00 (0.93-1.08)	11,138	810 (7.3)	1.14 (1.03-1.27)	1.00 (0.90-1.12)
PER	2,778	198 (7.1)	1.02 (0.87-1.19)	0.88 (0.75-1.04)	296	11 (3.7)	0.56 (0.31-1.03)	0.48 (0.26-0.89)
**Men (N=25,894)**								
SER	10,133	893 (8.8)	1	1	6,216	492 (7.9)	1	1
SER/HI	5,999	346 (5.8)	0.63 (0.56-0.72)	0.76 (0.66-0.86)	3,970	201 (5.1)	0.62 (0.52-0.73)	0.73 (0.61-0.88)
SSER	8,554	775 (9.1)	1.03 (0.93-1.14)	0.97 (0.88-1.08)	2,927	245 (8.4)	1.06 (0.91-1.25)	1.01 (0.86-1.19)
PER	1,208	122 (10.1)	1.16 (0.95-1.42)	1.02 (0.83-1.25)	123	13 (10.6)	1.37 (0.77-2.46)	1.31 (0.73-2.36)

OR: unadjusted Odds Ratio; aOR: Odds Ratio adjusted by age, gender, education, country of birth, family composition, economic sector, unemployment, and SA due to diagnoses other than CMD in the baseline (2016); *Models conditioned to have the same exposure for three consecutive years (2014-2016). Note: Persons entitled to sickness absence benefits are those having an annual income from paid employment of at least 24% of the price base amount of 2016; that is, 10,632 SEK. Area of residence and occupation barely changed the estimates; therefore, they were not included in the models as confounders.

For peer review only

For peer review only - http://bmjopen.bmj.com/site/about/guidelines.xhtml

We tested interaction effects between gender and employment quality. Women in precarious employment had a 27% lower risk of sickness absence benefits compared to men in standard employment after controlling for the same potential confounding factors (data not shown). The effect of the interaction term was somewhat pronounced for women in PER, but, in general, differences between gender and employment quality categories were relatively small. However, when comparing the model without the interaction term to the model containing it, the likelihood ratio test was not statistically significant (p-value>0.3955) (data not shown). This suggests that including the interaction term does not improve the fit of the model. Therefore, we have kept the original model without the interaction term.

The employment quality items most strongly related to the use of SABs due to CMD were income and unionization levels (Table 3). In the fully adjusted model, high income was associated with a reduced probability of SABs due to CMD (120-199% of the median income - aOR: 0.79, 95% CI: 0.72-0.85; and more than 199% of the median income - aOR: 0.61, 95% CI: 0.49-0.75), while the lowest-income level showed slight differences (lower than 60% of the median income – aOR: 0.92, 95% CI: 0.82-1.03). Women with the highest-income levels exhibit lower aORs compared to men (aOR: 0.48, 95% CI: 0.33-0.71 and aOR: 0.71, 95% CI: 0.54-0.92, respectively). Low unionization coverage was associated with lower use of SABs (less than 70% of unionization level - aOR: 0.82, 95% CI: 0.68-0.99), particularly among women (0.77, 95% CI: 0.58-1.03, near to significance). Similar results were found for women and men with medium unionization coverage. The unadjusted ORs and two different model adjustments can be observed in Supplementary Table 5.

For peer review only

For peer review only - http://bmjopen.bmj.com/site/about/guidelines.xhtml

Table 3. Association between employment quality dimensions (2016, baseline) and sickness absence benefits due to CMD in 2017 (N=78,215) among people entitled to sickness absence benefits in 2016.

			Women (N=52,321)		Men (N=25,894)	
	Cases N (%)	aOR (95% CI)	Cases N (%)	aOR (95% CI)	Cases N (%)	aOR (95% CI)
**Contractual relationship insecurity**						
Directly employed by employer	5,788 (7.5)	1	3,683 (7.1)	1	2,105 (8.2)	1
Employed by an agency	60 (8.2)	0.96 (0.73-1.25)	29 (6.5)	0.85 (0.58-1.24)	31 (10.9)	1.08 (0.74-1.58)
**Contractual temporariness**						
Stable employment	4,351 (7.4)	1	2,792 (7.1)	1	1,559 (8.0)	1
Unstable employment	1,497 (7.8)	1.02 (0.95-1.09)	920 (7.2)	1.01 (0.92-1.10)	577 (9.0)	1.04 (0.93-1.16)
**Multiple jobs/economic sectors**						
1-2 jobs	5,436 (7.4)	1	3,463 (7.1)	1	1,973 (8.2)	1
3 or more jobs	279 (7.8)	1.05 (0.93-1.20)	175 (7.0)	1.00 (0.85-1.18)	104 (9.6)	1.18 (0.95-1.46)
3 or more jobs in 3 or more sectors	133 (8.2)	1.09 (0.91-1.31)	74 (7.3)	1.04 (0.81-1.32)	59 (9.6)	1.19 (0.90-1.57)
**Income level**						
80-119% of the median	3,113 (7.7)	1	1,876 (7.1)	1	1,237 (8.9)	1
<60% of the median	395 (7.6)	0.92 (0.82-1.03)	319 (7.3)	0.91 (0.81-1.04)	76 (8.9)	0.94 (0.73-1.20)
60-79% of the median	1,415 (8.9)	1.10 (1.03-1.18)	1,111 (8.4)	1.08 (1.00-1.17)	304 (10.9)	1.16 (1.01-1.33)
120-200% of the median	831 (5.8)	0.79 (0.72-0.85)	378 (5.1)	0.79 (0.70-0.88)	453 (6.4)	0.80 (0.71-0.89)
>200% of the median	94 (4.1)	0.61 (0.49-0.75)	28 (2.9)	0.48 (0.33-0.71)	66 (4.9)	0.71 (0.54-0.92)
**Unionization level**						
>90%	5,409 (7.5)	1	3,497 (7.2)	1	1,912 (8.3)	1
70-90%	315 (6.8)	0.82 (0.73-0.92)	161 (6.1)	0.83 (0.70-0.98)	154 (7.7)	0.80 (0.67-0.96)
<70%	124 (7.0)	0.82 (0.68-0.99)	54 (5.8)	0.77 (0.58-1.03)	70 (8.2)	0.84 (0.65-1.09)

aOR: Odds Ratio adjusted mutually by the other employment quality dimensions and age, gender, education, country of birth, family composition, economic sector, unemployment, and SA due to diagnoses other than CMD in the baseline (2016); Note: Persons entitled to sickness absence benefits are those having an annual income from paid employment of at least 24% of the price base amount of 2016; that is, 10,632 SEK. *Income level categories based on the median of the Swedish working population after applying the inclusion and some of the main exclusion criteria (see methods section). *Income level categories based on the median of the Swedish working population (375,300 SEK) after applying several inclusion and exclusion criteria (see methods section). Income category boundaries: <60% of the median (225,180 SEK), 60-79% of the median (225,180-296,487 SEK), 80-119% of the median (300,240-446,607 SEK), 120-199% of the median (450,360-746,847 SEK), and ≥200% of the median (750,600 SEK). Area of residence and occupation barely changed the estimates; therefore, they were not included in the models as confounders.

For peer review only - http://bmjopen.bmj.com/site/about/guidelines.xhtml

DISCUSSION

This study indicated that having a PER was associated with a lower probability of using SABs due to CMD compared to SER, especially among women. On the other side of the spectrum, we also found that SER/HI was strongly and negatively associated with SABs. The income and unionization level were the themes of employment quality most strongly related to using SABs due to CMD: having high income and low unionization levels were associated with lower use of SABs due to CMD, particularly, among women.

Our results on the negative association between PER and the use of SABs due to CMD contrast with previous studies that found an increased prevalence of self-reported SA due to health problems among precariously employed workers, in Sweden and other countries [17,18]. In our present study, sickness presenteeism (SP) may explain the lower odds of SABs due to CMD in the PER group. Precariously employed workers who typically have low-income jobs are likely to incur SP because they cannot afford to use SABs. Using SABs results in a period of reduced income since the SABs only partially replace wages [35]. Additionally, SP may stem from the fear of being dismissed due to employment insecurity or from the lack of union protection [9,36]. An earlier study found that workers who transitioned from temporary to permanent employment had an increased probability of SA [8]. Precariously employed workers in our study population had the lowest unionization levels and low unionization was related to lower use of SABs due to CMD. These findings are consistent with previous studies in Germany and Norway where trade union members had higher SA than non-members (45,46). A second possible explanation for the low use of SABs among precariously employed workers is related to legal uncertainty when the Social Insurance Agency assesses entitlement to SABs for non-standard employees [37]. If temporary employees have scheduled work, they receive SABs for the days that they would have worked had the illness not reduced their work capacity by at least 25%. Non-standard employees however often lack a formal schedule covering the days in question, e.g., on-demand employees get their shifts assigned on a day-to-day basis or have a schedule based on an oral agreement, and risk being considered unemployed in the event of sickness and cannot access SABs from the employer. To access SABs, work capacity must be assessed, and this is done differently according to employment status. The work capacity of employees is assessed only relative to their current employment for the first 90 days of SA and only later against all jobs in the entire labour market, but unemployed (or non-standard workers without scheduled hours) will be assessed relative to all jobs in the entire labour market from the start. In practice, this often means that they do not qualify for SABs, since other jobs may exist in the labour market where their current functional ability does not impose a sufficient reduction in work capacity. However, we may bear in mind that this explanation is less plausible for workers with CMD that may have a reduced work capacity for any task.

In general, women had lower use of SABs in any employment quality typology compared to the SER, and the association was more pronounced in women than in men. These results were unexpected since women usually have higher SA rates than men [38]. However, our study population only included individuals with SSRI treatment (two-thirds of them women), which has not been studied before with this study design [39]. The lower use of SABs among women is plausible because it has been suggested that women may feel a greater responsibility to their colleagues and avoid burdening them by cooperating more when performing work tasks compared to men [35]. Furthermore, another study showed that female-dominated jobs related to jobs with responsibility for others such as education, human health and social work activities are more prone to SP [40]. However, this explanation is unlikely to apply to our study since we

For peer review only - http://bmjopen.bmj.com/site/about/guidelines.xhtml

accounted for the potential effect of women's concentration in certain sectors on SABs by controlling for the sector in the analysis. Women with a consecutive 3-year period in PER had the lowest use of SABs. This could be explained by the fact that women are more frequently employed in temporary employment, which has been related to fewer days of SABs than permanently employed and, probably, to lower access to SABs for the reasons previously mentioned [37].

SER/HI showed the highest income levels and we found that having a high income was related to a lower likelihood of using SABs. In this line, a Finnish study found that a higher position by education, occupational class and income was related to lower SA [41]. Another possible explanation for the lower use of SABs in employees in SER/HI is also SP, but for different reasons than the PER group. In a study conducted in Norway and Sweden, having supervisory responsibilities at work that no one else can perform was an influential factor for SP, which could be the case for the high earners in the present study [35]. Another driver for SP among high earners could be work satisfaction since they probably have jobs with more creativity, freedom and control [35,42]. Another explanation is that high earners have more knowledge-intensive occupations where they can work from home rather than take SA [43]. Lastly, individuals in SER/HI could plausibly be more likely to combine SSRIs with cognitive behavioural therapy (CBT) or mindfulness which could be, to a certain extent, protective of work capacity [44,45].

SA can also be conceptualised as a health status measure. Accordingly, an important consequence of SP is an increased risk of more severe and long-lasting health problems that, in turn, lead to longer periods of SA, regardless of the employment quality typology followed [46].

LIMITATIONS AND STRENGTHS

Limitations of this study could arise from the design of the study when analysing individuals with three years with the same exposure since those with PE are healthier enough to not exit the labour market, which could underestimate the results. However, we also analysed one year of exposure before SABs, finding comparable results which may overcome the previous limitation. We cannot rule out that some people in the study population were not entitled to SABs since apart from the prior income requirement, it is necessary to have a reduced work capacity due to the CMD of at least 25%, which is not possible to assess in the datasets. Selection bias could be introduced among SER/HI because CBT and other psychotherapies could be more prevalent in competing with SSRIs. It could be thought that we could have included a longer period for outcome measurement. However, our main interest is using SSRI as an indicator of having a new diagnosis of CMD and our approach is conservative in considering SABs close to SSRI since there is mixed evidence about the long-term efficacy of SSRIs [29,47]. Another limitation is that we did not have access to a level of detail of work to explore whether a person in non-standard arrangements is scheduled for work or is between work periods. Further research with alternative data sources is needed in this regard.

One of the strengths of this study is that it is based on population registers with high validity and low attrition rates, which allows for accurate and objective measurement of PE through a multi-dimensional measure [48]. Similarly, the linkage of pharmaceutical and work disability registers with the exact dates of SSRI prescription and SABs allowed for an accurate assessment of the outcome. The study is based on a large subpopulation of the Swedish working population. Lastly, the longitudinal study design reduces reverse causation (i.e., health selection into precarious employment).

For peer review only - http://bmjopen.bmj.com/site/about/guidelines.xhtml

GENERALIZABILITY

The results of this study are specific to our Swedish study population with a prescription of SSRIs and may not be generalisable to other countries with large variations in the treatments available for CMDs, subsidisation of SSRIs costs, and SAB regulations.

CONCLUDING REMARKS

This study observed a trend of lower use of SABs among people with CMD in PER and SER/HI than in SER, particularly, women with a period of three consecutive years in PER. Accordingly our findings suggest that precariously employed workers may face barriers to accessing SABs. More inclusive policies are recommended to reduce these obstacles and guarantee SABs for those who are in PER and/or reduce involuntary forms of SP.

Contributors

TB conceived the initial idea of the study and act as a guarantor of the work. All authors contributed to the design of the study. TB and JCH-R further developed the study design and discussed it with NM-S. Data management and data analysis were performed by JCH-R with support from NM-S. All authors contributed to the interpretation of the results. JCH-R drafted the first version of the manuscript, and all authors made subsequent revisions of the work, agreed on the text and findings, and approved the final version. The corresponding author certifies that all listed authors meet authorship criteria.

Funding

This research was funded by FORTE—Swedish Research Council for Health Working Life and Welfare. Grant number 2021-00034.

Acknowledgements

The authors are grateful to all the researchers of the PWR Consortium and acknowledge Anette Linnersjö for ordering and receiving data and the valuable input on the data management. The authors also acknowledge feedback provided by Cecilia Orellana and Fabrizio Méndez Rivero during various stages of the development of the study.

Competing interests

The authors declare no conflict of interest. The funder had no role in the design of the study; in the collection, analysis, or interpretation of data; in the writing of the manuscript, or in the decision to publish the results.

Patient and public involvement

Patients and/or the public were not involved in the design, or conduct, or reporting, or dissemination plans of this research.

Patient consent for publication

Not required.

Ethics approval

The Regional Ethics Board of Stockholm granted ethical permission for the study (no. 2017/1224-31/2 and 2018/1675-32). This is a register-based study and informed consent is not applicable.

For peer review only - http://bmjopen.bmj.com/site/about/guidelines.xhtml

For peer review only

Data availability statement

Data may be obtained from a third party and are not publicly available.

REFERENCES

1 Rodgers G. Labour Market Flexibility and Decent Work. ;:11.

2 Berglund T, Håkansson K, Isidorsson T. Occupational change on the dualised Swedish labour market. *Econ Ind Democr* 2022;**43**:918–42. doi:10.1177/0143831X20959714

3 Kreshpaj B, Orellana C, Burström B, *et al.* What is precarious employment? A systematic review of definitions and operationalizations from quantitative and qualitative studies. *Scand J Work Environ Health* 2020;**46**:235–47. doi:10.5271/sjweh.3875

4 Benach J, Muntaner C. Precarious employment and health: developing a research agenda. *J Epidemiol Community Health* 2007;**61**:276–7. doi:10.1136/jech.2005.045237

5 Global, regional, and national burden of 12 mental disorders in 204 countries and territories, 1990–2019: a systematic analysis for the Global Burden of Disease Study 2019. *Lancet Psychiatry* 2022;**9**:137–50. doi:10/gn7nxg

6 OECD. Mental Health and Work: Sweden. 2013. https://doi.org/10.1787/9789264188730-en (accessed 12 Jul 2022).

7 Swedish Social Insurance Agency [Försäkringskassan]. Social Insurance in Figures 2021. 2021.

8 Virtanen M, Kivimäki M, Elovainio M, *et al.* From insecure to secure employment: changes in work, health, health related behaviours, and sickness absence. *Occup Environ Med* 2003;**60**:948–53. doi:10.1136/oem.60.12.948

9 Johns G. Presenteeism in the workplace: A review and research agenda. *J Organ Behav* 2010;**31**:519–42. doi:10.1002/job.630

10 Matsaganis M, Özdemir E, Ward T, *et al.* Non-standard employment and access to social security benefits. 2015;:43.

11 Schill HP, Bjurström S, Pietrek J, *et al.* The Swedish sickness insurance and temporary employment. 2016;:5.

12 Allebeck P, Mastekaasa A. Chapter 5. Risk factors for sick leave - general studies. *Scand J Public Health* 2004;**32**:49–108. doi:10/dvr35c

13 Virtanen M, Kivimaki M, Joensuu M, *et al.* Temporary employment and health: a review. *Int J Epidemiol* 2005;**34**:610–22. doi:10.1093/ije/dyi024

14 Mastekaasa A. Unionization and Certified Sickness Absence: Norwegian Evidence. *ILR Rev* 2013;**66**:117–41. doi:10.1177/001979391306600105

For peer review only

15 Goerke L, Pannenberg M. Trade union membership and sickness absence: Evidence from a sick pay reform. *Labour Econ* 2015;**33**:13–25. doi:10.1016/j.labeco.2015.02.004

16 de Vries H, Fishta A, Weikert B, *et al.* Determinants of Sickness Absence and Return to Work Among Employees with Common Mental Disorders: A Scoping Review. *J Occup Rehabil* 2018;**28**:393–417. doi:10.1007/s10926-017-9730-1

17 Oke A, Braithwaite P, Antai D. Sickness Absence and Precarious Employment: A Comparative Cross-National Study of Denmark, Finland, Sweden, and Norway. *Int J Occup Environ Med* 2016;**7**:125–47. doi:10/gmx883

18 Matilla-Santander N, González-Marrón A, Martín-Sánchez JC, *et al.* Precarious employment and health-related outcomes in the European Union: a cross-sectional study. *Crit Public Health* 2020;**30**:429–40. doi:10.1080/09581596.2019.1587385

19 Ronnblad T, Gronholm E, Jonsson J, *et al.* Precarious employment and mental health: a systematic review and meta-analysis of longitudinal studies. *Scand J Work Env Health* 2019;**45**:429–43. doi:10.5271/sjweh.3797

20 Jonsson J, Muntaner C, Bodin T, *et al.* Low-quality employment trajectories and risk of common mental disorders, substance use disorders and suicide attempt: a longitudinal study of the Swedish workforce. *Scand J Work Env Health* 2021;**47**:208–16. doi:10/gmprvj

21 Sickness benefit for those with sole proprietorship. http://www.forsakringskassan.se/english/sick/sole-proprietorship/sickness-benefit-for-those-with-sole-proprietorship (accessed 29 Sep 2022).

22 Hutchins DS, Zeber JE, Roberts CS, *et al.* Initial Medication Adherence—Review and Recommendations for Good Practices in Outcomes Research: An ISPOR Medication Adherence and Persistence Special Interest Group Report. *Value Health* 2015;**18**:690–9. doi:10.1016/j.jval.2015.02.015

23 Aznar-Lou I, Iglesias-González M, Gil-Girbau M, *et al.* Impact of initial medication non-adherence to SSRIs on medical visits and sick leaves. *J Affect Disord* 2018;**226**:282–6. doi:10.1016/j.jad.2017.09.057

24 Bodin T, Jonsson J. Swedish Register Operationalization of Precarious Employment. 2020.https://github.com/TBodin/swe-rope (accessed 15 Mar 2021).

25 Jonsson J, Matilla-Santander N, Kreshpaj B, *et al.* Exploring multidimensional operationalizations of precarious employment in Swedish register data – a typological approach and a summative score approach. *Scand J Work Environ Health* 2021;**47**:117–26. doi:10.5271/sjweh.3928

26 Pollack R, Kreshpaj B, Jonsson J, *et al.* Low-quality employment trajectories and the risk of common mental health disorders among individuals with Swedish and foreign background - a register-based cohort study. *Scand J Work Environ Health* 2022;**48**:351–60. doi:10.5271/sjweh.4029

For peer review only - http://bmjopen.bmj.com/site/about/guidelines.xhtml

27 Overview - SSRI antidepressants. nhs.uk. 2021.https://www.nhs.uk/mental-health/talking-therapies-medicine-treatments/medicines-and-psychiatry/ssri-antidepressants/overview/ (accessed 21 Jul 2022).

28 Machado-Vieira R, Baumann J, Wheeler-Castillo C, *et al.* The Timing of Antidepressant Effects: A Comparison of Diverse Pharmacological and Somatic Treatments. *Pharmaceuticals* 2010;**3**:19–41. doi:10.3390/ph3010019

29 Cheng Q, Huang J, Xu L, *et al.* Analysis of Time-Course, Dose-Effect, and Influencing Factors of Antidepressants in the Treatment of Acute Adult Patients With Major Depression. *Int J Neuropsychopharmacol* 2020;**23**:76–87. doi:10.1093/ijnp/pyz062

30 Swedish Social Insurance Agency [Försäkringskassan]. Social Insurance in Figures 2020. 2020.

31 Roelen C a. M, Koopmans PC, Schreuder J a. H, *et al.* The history of registered sickness absence predicts future sickness absence. *Occup Med Oxf Engl* 2011;**61**:96–101. doi:10/ct43k7

32 Ármannsdóttir B, Mårdby A-C, Haukenes I, *et al.* Cumulative incidence of sickness absence and disease burden among the newly sick-listed, a cross-sectional population-based study. *BMC Public Health* 2013;**13**:329. doi:10.1186/1471-2458-13-329

33 Staland-Nyman C, Houkes I, de Rijk A, *et al.* Gender equality in domestic work and sickness absence - a population-based study on women and men in Sweden. *Women Health* 2021;**61**:325–36. doi:10.1080/03630242.2021.1872759

34 Amrhein V, Greenland S, McShane B. Scientists rise up against statistical significance. *Nature* 2019;**567**:305–7. doi:10.1038/d41586-019-00857-9

35 Johansen V, Aronsson G, Marklund S. Positive and negative reasons for sickness presenteeism in Norway and Sweden: a cross-sectional survey. *BMJ Open* 2014;**4**:e004123. doi:10.1136/bmjopen-2013-004123

36 Aronsson G, Gustafsson K. Sickness Presenteeism: Prevalence, Attendance-Pressure Factors, and an Outline of a Model for Research. *J Occup Environ Med* 2005;**47**:958–66.

37 Inspektionen för socialförsäkringen. Tidsbegränsade anställningar och sjukförsäkringen 2016. 2016.

38 *Socialförsäkringen i siffror 2022*. Stockholm: : Försäkringskassan 2022.

39 Lytsy P, Alexanderson K, Friberg E. Gender differences in treatment with antidepressants during first weeks of a sick-leave spell due to depressive episode. *Eur J Public Health* 2020;**30**:299–304. doi:10.1093/eurpub/ckz172

40 Marklund S, Gustafsson K, Bergström G, *et al.* Reasons for presenteeism in different occupational branches in Sweden: a population based cross-sectional

For peer review only

For peer review only - http://bmjopen.bmj.com/site/about/guidelines.xhtml

study. *Int Arch Occup Environ Health* 2021;**94**:1385–95. doi:10.1007/s00420-021-01701-2

41 Piha K, Laaksonen M, Martikainen P, *et al.* Interrelationships between education, occupational class, income and sickness absence. *Eur J Public Health* 2010;**20**:276–80. doi:10.1093/eurpub/ckp162

42 Hansen CD, Andersen JH. Going ill to work – What personal circumstances, attitudes and work-related factors are associated with sickness presenteeism? *Soc Sci Med* 2008;**67**:956–64. doi:10.1016/j.socscimed.2008.05.022

43 Steidelmüller C, Meyer S-C, Müller G. Home-Based Telework and Presenteeism Across Europe. *J Occup Environ Med* 2020;**62**:998–1005. doi:10.1097/JOM.0000000000001992

44 Roshanaei-Moghaddam B, Pauly MC, Atkins DC, *et al.* Relative effects of CBT and pharmacotherapy in depression versus anxiety: is medication somewhat better for depression, and CBT somewhat better for anxiety? *Depress Anxiety* 2011;**28**:560–7. doi:10.1002/da.20829

45 Grossman P, Niemann L, Schmidt S, *et al.* Mindfulness-based stress reduction and health benefits: A meta-analysis. *J Psychosom Res* 2004;**57**:35–43. doi:10.1016/S0022-3999(03)00573-7

46 Taloyan M, Aronsson G, Leineweber C, *et al.* Sickness Presenteeism Predicts Suboptimal Self-Rated Health and Sickness Absence: A Nationally Representative Study of the Swedish Working Population. *PLOS ONE* 2012;**7**:e44721. doi:10.1371/journal.pone.0044721

47 The serotonin theory of depression: a systematic umbrella review of the evidence | Molecular Psychiatry. https://www.nature.com/articles/s41380-022-01661-0 (accessed 4 Oct 2022).

48 Ludvigsson JF, Svedberg P, Olén O, *et al.* The longitudinal integrated database for health insurance and labour market studies (LISA) and its use in medical research. *Eur J Epidemiol* 2019;**34**:423–37. doi:10.1007/s10654-019-00511-8

For peer review only

For peer review only - http://bmjopen.bmj.com/site/about/guidelines.xhtml

Figures legend:

Figure 1. Flow chart of the total study population.

This is a flow chart of the exclusion criteria applied (there is no legend)

Figure 2. Directed Acyclic Graph (DAG) of the relation between employment quality typologies (EQT) and sickness absence benefits (SABs) due to common mental disorders (CMD).

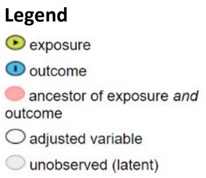

For peer review only

For peer review only - http://bmjopen.bmj.com/site/about/guidelines.xhtml

Figure 1. Flow chart of the total study population.

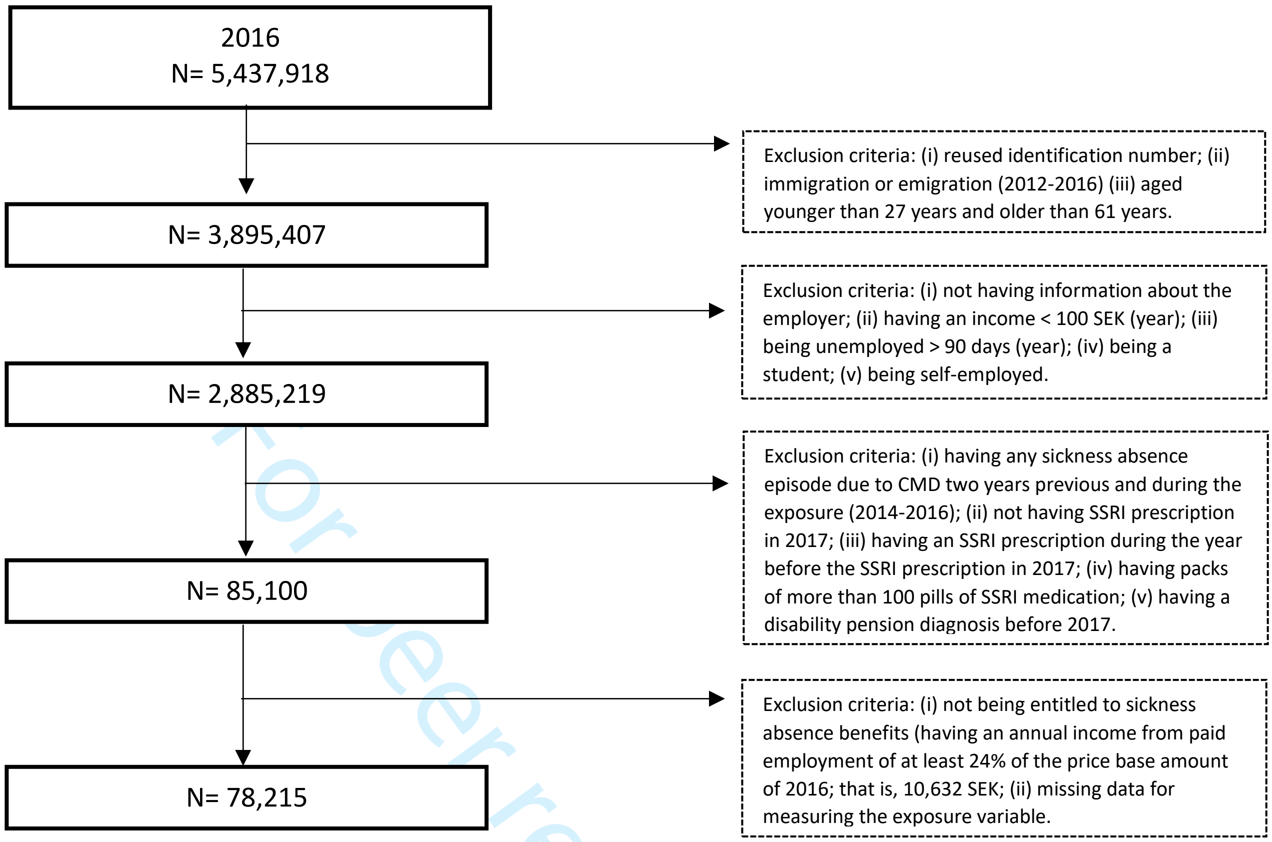

For peer review only

For peer review only - http://bmjopen.bmj.com/site/about/guidelines.xhtml

Figure 2. Directed Acyclic Graph (DAG) of the relation between employment quality typologies (EQT) and sickness absence benefits (SABs) due to common mental disorders (CMD).

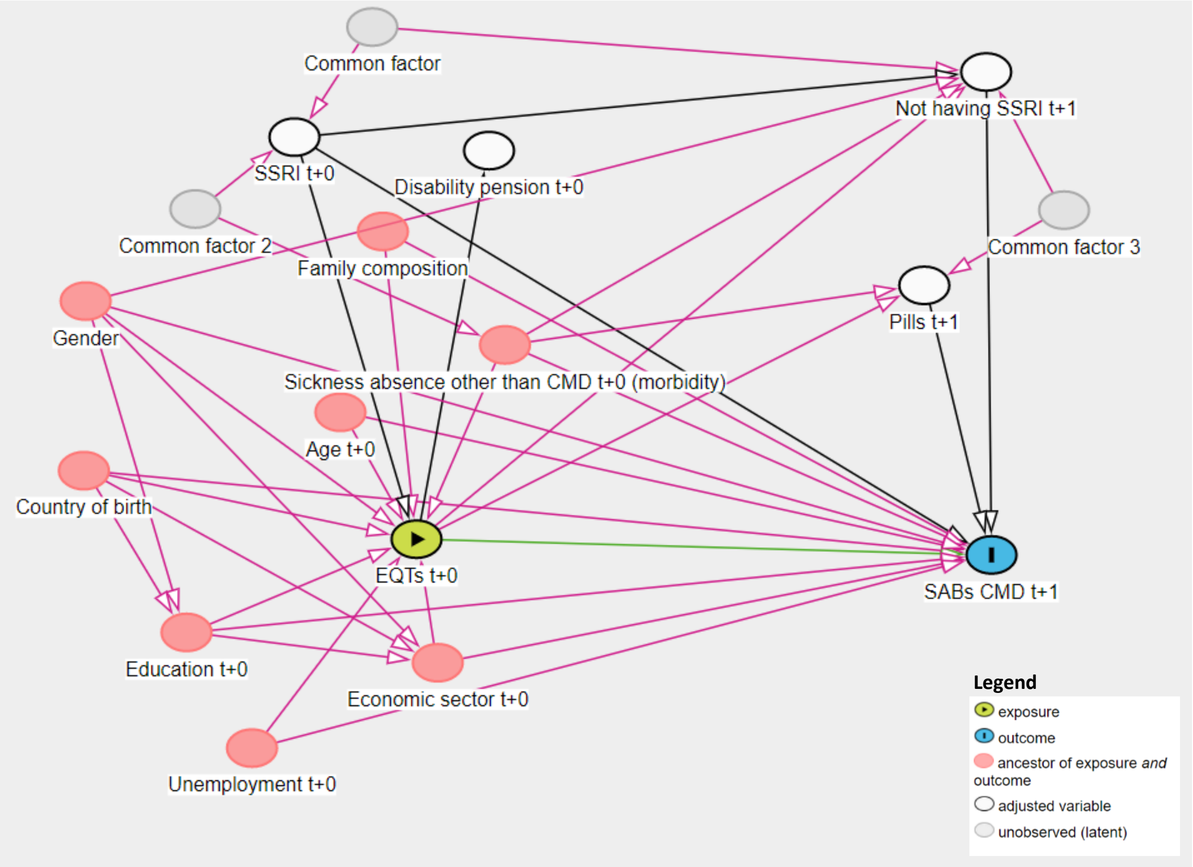

Minimal sufficient adjustment sets for estimating the direct effect of EQTs t+0 (baseline, 2016) on SABs due to CMD t+1 is age, gender, education, country of birth, family composition, economic sector, unemployment, and sickness absence due to diagnoses other than CMD, having Selective Serotonin Reuptake Inhibitors (SSRI) prescription during the year before the SSRI prescription in 2017, having disability pension before 2017, having packs of more than 100 pills in 2017 and not having Selective Serotonin Reuptake Inhibitors SSRI prescriptions close to the SABs in 2017.

For peer review only - http://bmjopen.bmj.com/site/about/guidelines.xhtml

Supplementary Table 1. Sociodemographic and employment-related characteristics at baseline (2016) of the Swedish working population and the study population with common mental disorders.

	**Population***	**Study population****
	N (%)	N (%)
**Age**		
27-40 years	1,044,971 (37.4)	30,675 (39.2)
41-51 years	976,697 (34.9)	28,541 (36.5)
52-61 years	774,838 (27.7)	18,999 (24.3)
**Gender**		
Male	1,412,310 (50.5)	25,894 (33.1)
Female	1,384,196 (49.5)	52,321 (66.9)
**Education level**		
Elementary/high school education	1,518,999 (54.4)	41,072 (52.5)
Higher education <3 years	429,648 (15.4)	11,456 (14.7)
Higher education ≥3 years	845,355 (30.3)	25,651 (32.8)
**Country of birth**		
Sweden	2,459,096 (88.0)	69,736 (89.2)
Not Sweden	335,518 (12.0)	8,438 (10.8)
**Family composition**		
Couple with children	1,400,506 (50.1)	37,471 (47.9)
Couple without children	352,101 (12.6)	8,421 (10.8)
Single with children	260,276 (9.3)	10,243 (13.1)
Single without children	783,622 (28.0)	22,080 (28.2)
**Unemployment**		
0 days unemployed	2,718,662 (97.2)	76,090 (97.3)
From 1 to 90 days unemployed	77,844 (2.8)	2,125 (2.7)
**Economic sector**		
Private	1,741,419 (62.3)	41,653 (53.3)
Public	1,055,087 (37.7)	36,562 (46.7)
TOTAL	2,796,506 (100.0)	78,215 (100.0)

*The entire study population meeting the inclusion and exclusion criteria (except having SSRI prescriptions in 2017); **The final study population meeting the whole inclusion and exclusion criteria. Missing values in educational level (population: 2,504, 0.09%; study population: 41, 0.05%), country of birth (population: 1892, 0.07%; study population: 41, 0.05%) and family composition (population: 1, <0.01%).

For peer review only

Supplementary Table 2. Incidence of a first sickness absence episode due to common mental disorders in a cohort of 78,215 individuals with common mental disorders in 2017, aged 27-61, entitled to sickness absence benefits in 2016, by sociodemographic and employment characteristics at baseline (2016)

	**Sickness absence benefits (7.48%)**
	N (%)
**Age**	
27-40 years	2,808 (9.2)
41-51 years	1,901 (6.7)
52-61 years	1,139 (6.0)
**Gender**	
Male	2,136 (8.2)
Female	3,712 (7.1)
**Education level**	
Elementary/high school education	3,502 (8.5)
Higher education <3 years	793 (6.9)
Higher education ≥3 years	1,547 (6.0)
**Country of birth**	
Sweden	5,058 (7.3)
Not Sweden	783 (9.3)
**Family composition**	
Couple with children	2,678 (7.1)
Couple with no children	519 (6.2)
Single with children	808 (7.9)
Single	1,843 (8.3)
**Unemployment**	
0 days unemployed	5,697 (7.5)
From 1 to 90 days unemployed	151 (7.1)
**Economic sector**	
Private	3218 (7.7)
Public	2630 (7.2)
**Employment quality typology**	
Standard Employment Relationship/HI	593 (5.2)
Standard Employment Relationship	2,268 (7.6)
Sub-standard Employment Relationship	2,667 (8.1)
Precarious Employment Relationship	320 (8.0)
**Contractual relationship insecurity**	
Directly employed by an employer	5,788 (7.5)
Employed by an agency	60 (8.2)
**Contractual temporariness**	
Stable employment	4,351 (7.4)
Unstable employment	1,497 (7.8)
**Multiple jobs/economic sectors**	
1-2 jobs	5,436 (7.4)
3 or more jobs	279 (7.8)
3 or more jobs in 3 or more sectors	133 (8.2)
**Income level***	
<60% of the median	395 (7.6)
60-79% of the median	1,415 (8.9)
80-119% of the median	3,113 (7.7)
120-200% of the median	831 (5.7)
>200% of the median	94 (4.0)
**Unionization level**	
<70 %	124 (7.0)
70-90%	315 (6.8)
>90%	5,409 (7.5)

Missing values in the educational level (36, 0.05%) and country of birth (41, 0.05%). Note: Persons entitled to sickness absence benefits in 2016 are those having an annual income from paid employment of at least 24% of the price base amount of 2016; that is, 10,632 SEK. *Income level categories based on the median of the Swedish working population (375,300 SEK) after applying several inclusion and exclusion criteria (see methods section). Income category boundaries: <60% of the median (225,180 SEK), 60-79% of the median (225,180-296,487 SEK), 80-119% of the median (300,240-446,607 SEK), 120-199% of the median (450,360-746,847 SEK), and ≥200% of the median (750,600 SEK).

For peer review only

For peer review only - http://bmjopen.bmj.com/site/about/guidelines.xhtml

Supplementary Table 3. Incidence of a first sickness absence episode due to common mental disorders in 2017 in women and men in a cohort of 85,164 individuals with common mental disorders, aged 27-61, entitled to sickness absence benefits in 2016, by sociodemographic and employment characteristics at baseline (2016).

	Sickness absence benefits	
	**Women (7.1%)**	**Men (8.2%)**
	N (%)	N (%)
**Age**		
27-40 years	1782 (8.7)	1026 (10.2)
41-51 years	1204 (6.3)	697 (7.4)
52-61 years	726 (5.8)	413 (6.4)
**Education level**		
Elementary/high school education	2016 (8.0)	1486 (9.3)
Higher education <3 years	484 (6.3)	309 (8.2)
Higher education ≥3 years	1208 (6.2)	339 (5.5)
**Country of birth**		
Sweden	3195 (6.9)	1863 (8.0)
Not Sweden	512 (8.8)	271 (10.4)
**Family composition**		
Couple with children	1735 (6.8)	943 (7.8)
Couple without children	357 (6.2)	162 (6.2)
Single with children	647 (7.6)	161 (9.2)
Single without children	973 (7.7)	870 (9.2)
**Unemployment**		
0 days unemployed	3632 (7.1)	2065 (8.2)
From 1 to 90 days unemployed	80 (6.0)	71 (8.9)
**Economic sector**		
Private	1,594 (7.0)	1,624 (8.5)
Public	2,118 (7.1)	512 (7.4)
**Employment quality typology**		
Standard Employment Relationship/HI	247 (4.5)	346 (5.8)
Standard Employment Relationship	1375 (7.0)	893 (8.8)
Sub-standard Employment Relationship	1892 (7.7)	775 (9.1)
Precarious Employment Relationship	198 (7.1)	122 (10.1)
**Contractual relationship insecurity**		
Directly employed by an employer	3683 (7.1)	2105 (8.2)
Employed by an agency	29 (6.5)	31 (10.9)
**Contractual temporariness**		
Stable employment	2792 (7.0)	1559 (8.0)
Unstable employment	920 (7.2)	577 (9.0)
**Multiple jobs/economic sectors**		
1-2 jobs	3463 (7.1)	1973 (8.2)
3 or more jobs	175 (7.0)	104 (9.6)
3 or more jobs in 3 or more sectors	74 (7.3)	59 (9.6)
**Income level**		
<60% of the median	319 (7.3)	76 (8.9)
60-79% of the median	1111 (8.4)	304 (10.9)
80-119% of the median	1876 (7.1)	1237 (8.9)
120-200% of the median	378 (5.1)	453 (6.4)
>200% of the median	28 (2.9)	66 (4.9)
**Unionization level**		
<70 %	54 (5.8)	70 (8.2)
70-90%	161 (6.1)	154 (7.7)
>90%	3497 (7.2)	1912 (8.3)

Missing values in education level (women: 19, 0.04%; men: 17, 0.07%) and country of birth (women: 19, 0.04%; men: 22, 0.08%). Note: Persons entitled to sickness absence benefits in 2016 are those having an annual income from paid employment of at least 24% of the price base amount of 2016; that is, 10,632 SEK. *Income level categories based on the median of the Swedish working population (375,300 SEK) after applying several inclusion and exclusion criteria (see methods section). Income category boundaries: <60% of the median (225,180 SEK), 60-79% of the median (225,180-296,487 SEK), 80-119% of the median (300,240-446,607 SEK), 120-199% of the median (450,360-746,847 SEK), and ≥200% of the median (750,600 SEK).

For peer review only

Supplementary Table 4. Incidence of a first sickness absence episode due to common mental disorders in a cohort of 85,164 individuals with common mental disorders in 2017, aged 27-61, entitled to sickness absence benefits in 2016, by employment quality typology (Standard Employment Relationship, SER; Standard Employment Relationship with high income, SER/HI; Sub-standard Employment Relationship, SSER; Precarious Employment Relationship, PER; baseline, 2016).

	Sickness absence benefits			
	**SER/HI (5.2%)**	**SER (7.6%)**	**SSER (8.1%)**	**PER (8%)**
	N (%)	N (%)	N (%)	N (%)
**Age**				
27-40 years	180 (6.3)	938 (9.6)	1481 (9.5)	209 (8.8)
41-51 years	253 (4.8)	794 (6.9)	773 (7.1)	81 (7.9)
52-61 years	160 (4.8)	536 (6.3)	413 (6.3)	30 (5.1)
**Gender**				
Male	346 (5.8)	893 (8.8)	775 (9.1)	122 (10.1)
Female	247 (4.5)	1375 (7.0)	1892 (7.7)	198 (7.1)
**Education level**				
Elementary/high school education	209 (6.2)	1340 (8.5)	1731 (9.0)	222 (8.8)
Higher education <3 years	116 (5.8)	321 (7.3)	311 (7.0)	45 (7.1)
Higher education ≥3 years	268 (4.4)	606 (6.4)	620 (6.7)	53 (6.4)
**Country of birth**				
Sweden	527 (5.0)	1962 (7.4)	2282 (7.8)	287 (8.1)
Not Sweden	66 (6.4)	305 (9.6)	379 (10.1)	33 (7.1)
**Family composition**				
Couple with children	288 (4.8)	971 (7.3)	1286 (7.9)	133 (7.5)
Couple without children	65 (4.8)	241 (6.6)	202 (6.4)	11 (3.8)
Single with children	59 (4.7)	304 (7.8)	396 (8.7)	49 (8.8)
Single without children	181 (6.4)	752 (8.4)	783 (8.7)	127 (9.3)
**Unemployment**				
0 days unemployed	590 (5.2)	2253 (7.6)	2589 (8.2)	265 (7.8)
From 1 to 90 days unemployed	3 (5.6)	15 (6.3)	78 (6.2)	55 (9.6)
**Economic sector**				
Private	384 (5.5)	1,186 (8.5)	1,383 (7.9)	265 (8.3)
Public	209 (4.7)	1,082 (6.8)	1,284 (8.3)	55 (6.8)
**Contractual relationship insecurity**				
Directly employed by an employer	593 (5.2)	2263 (7.6)	2636 (8.1)	296 (7.9)
Employed by an agency	0	5 (13.2)	31 (6.7)	24 (10.9)
**Contractual temporariness**				
Stable employment	593 (5.2)	2246 (7.7)	1503 (8.3)	9 (3.5)
Unstable employment	0	22 (4.7)	1164 (7.8)	311 (8.3)
**Multiple jobs/economic sectors**				
1-2 jobs	579 (5.2)	2220 (7.6)	2479 (8.1)	158 (7.3)
3 or more jobs	11 (5.0)	36 (8.2)	155 (8.1)	77 (7.7)
3 or more jobs in 3 or more sectors	3 (2.5)	12 (6.3)	33 (6.7)	85 (10.3)
**Income level**				
<60% of the median	0	0	270 (8.1)	125 (6.6)
60-79% of the median	0	0	1313 (8.9)	102 (8.8)
80-119% of the median	0	2203 (7.7)	822 (7.7)	88 (10.0)
120-200% of the median	536 (5.4)	42 (7.1)	249 (6.3)	4 (6.5)
>200% of the median	57 (3.5)	23 (4.5)	13 (7.1)	1 (100.0)
**Unionization level**				
<70%	0	1 (5.0)	58 (6.8)	65 (7.2)
70-90%	2 (3.6)	26 (6.3)	223 (6.8)	64 (7.4)
>90%	591 (5.2)	2241 (7.6)	2386 (8.3)	191 (8.6)

Missing values in education level (SER/HI: 7, 0.06%; SER: 10, 0.03%; SSER: 17, 0.05%; PE: 2, 0.05%) and country of birth (SER/HI: 3, 0.03%; SER: 18, 0.06%; SSER: 20, 0.06%). Note: Persons entitled to sickness absence benefits in 2016 are those having an annual income from paid employment of at least 24% of the price base amount of 2016; that is, 10,632 SEK. *Income level categories based on the median of the Swedish working population (375,300 SEK) after applying several inclusion and exclusion criteria (see methods section). Income category boundaries: <60% of the median (225,180 SEK), 60-79% of the median (225,180-296,487 SEK), 80-119% of the median (300,240-446,607 SEK), 120-199% of the median (450,360-746,847 SEK), and ≥200% of the median (750,600 SEK).

For peer review only

Supplementary Table 5. Association between employment quality dimensions (2016, baseline) and sickness absence benefits due to CMD in 2017 (N=78,215) among people entitled to sickness absence benefits in 2016.

					Women (N=52,321)				Men (N=25,894)			
	Cases N(%)	OR (95% CI)	aOR (95% CI) [1]	aOR (95% CI) [2]	Cases N(%)	OR (95% CI)	aOR (95% CI) [1]	aOR (95% CI) [2]	Cases N(%)	OR (95% CI)	aOR (95% CI) [1]	aOR (95% CI) [2]
**Contractual relationship insecurity**												
Directly employed by an employer	5,788 (7.5)	1	1	1	3,683 (7.1)	1	1	1	2,105 (8.2)	1	1	1
Employed by an agency	60 (8.2)	1.10 (0.85-1.44)	1.05 (0.80-1.37)	0.96 (0.73-1.25)	29 (6.5)	0.91 (0.62-1.32)	0.89 (0.61-1.31)	0.85 (0.58-1.24)	31 (10.9)	1.36 (0.94-1.98)	1.19 (0.81-1.73)	1.08 (0.74-1.58)
**Contractual temporariness**												
Stable employment	4,351 (7.4)	1	1	1	2,792 (7.1)	1	1	1	1,559 (8.0)	1	1	1
Unstable employment	1,497 (7.8)	1.07 (1.01-1.14)	1.08 (1.02-1.15)	1.02 (0.95-1.09)	920 (7.2)	1.03 (0.95-1.11)	1.06 (0.98-1.15)	1.01 (0.92-1.10)	577 (9.0)	1.14 (1.03-1.26)	1.12 (1.01-1.24)	1.04 (0.93-1.16)
**Multiple jobs/economic sectors**												
1-2 jobs	5,436 (7.4)	1	1	1	3,463 (7.1)	1	1	1	1,973 (8.2)	1	1	1
3 or more jobs	279 (7.8)	1.05 (0.93-1.19)	1.06 (0.93-1.20)	1.05 (0.93-1.20)	175 (7.0)	0.99 (0.85-1.16)	1.02 (0.87-1.20)	1.00 (0.85-1.18)	104 (9.6)	1.19 (0.97-1.47)	1.17 (0.95-1.45)	1.18 (0.95-1.46)
3 or more jobs in 3 or more sectors	133 (8.2)	1.11 (0.93-1.33)	1.11 (0.93-1.34)	1.09 (0.91-1.31)	74 (7.3)	1.04 (0.82-1.32)	1.07 (0.84-1.36)	1.04 (0.81-1.32)	59 (9.6)	1.19 (0.91-1.56)	1.16 (0.88-1.53)	1.19 (0.90-1.57)
**Income level**												
80-119% of the median	3,113 (7.7)	1	1	1	1,876 (7.1)	1	1	1	1,237 (8.9)	1	1	1
<60% of the median	395 (7.6)	0.98 (0.88-1.09)	0.98 (0.88-1.10)	0.92 (0.82-1.03)	319 (7.3)	1.03 (0.91-1.17)	1.05 (0.93-1.19)	0.91 (0.81-1.04)	76 (8.9)	1.00 (0.78-1.27)	0.99 (0.77-1.26)	0.94 (0.73-1.20)
60-79% of the median	1,415 (8.9)	1.16 (1.09-1.24)	1.17 (1.09-1.24)	1.10 (1.03-1.18)	1,111 (8.4)	1.20 (1.11-1.30)	1.21 (1.12-1.31)	1.08 (1.00-1.17)	304 (10.9)	1.24 (1.09-1.42)	1.25 (1.10-1.43)	1.16 (1.01-1.33)
120-200% of the median	831 (5.8)	0.73 (0.67-0.79)	0.72 (0.67-0.78)	0.79 (0.72-0.85)	378 (5.1)	0.70 (0.63-0.79)	0.70 (0.63-0.79)	0.79 (0.70-0.88)	453 (6.4)	0.70 (0.62-0.78)	0.69 (0.62-0.78)	0.80 (0.71-0.89)
>200% of the median	94 (4.1)	0.50 (0.41-0.62)	0.50 (0.40-0.62)	0.61 (0.49-0.75)	28 (2.9)	0.39 (0.26-0.57)	0.39 (0.26-0.56)	0.48 (0.33-0.71)	66 (4.9)	0.52 (0.41-0.68)	0.51 (0.40-0.66)	0.71 (0.54-0.92)
**Unionization level**												
>90%	5,409 (7.5)	1	1	1	3,497 (7.2)	1	1	1	1,912 (8.3)	1	1	1
70-90%	315 (6.8)	0.90 (0.80-1.01)	0.86 (0.77-0.97)	0.82 (0.73-0.92)	161 (6.1)	0.84 (0.72-0.99)	0.82 (0.70-0.97)	0.83 (0.70-0.98)	154 (7.7)	0.92 (0.78-1.10)	0.84 (0.71-1.00)	0.80 (0.67-0.96)
<70%	124 (7.0)	0.92 (0.77-1.11)	0.87 (0.72-1.05)	0.82 (0.68-0.99)	54 (5.8)	0.80 (0.61-1.06)	0.76 (0.58-1.01)	0.77 (0.58-1.03)	70 (8.2)	0.99 (0.77-1.27)	0.87 (0.67-1.12)	0.84 (0.65-1.09)

OR: unadjusted Odds Ratio; [1] aOR: Odds Ratio adjusted mutually by the other employment quality dimensions in the baseline (2016); [2] aOR: Odds Ratio adjusted mutually by the other employment quality dimensions and age, gender, education, country of birth, family composition, economic sector, unemployment, and SA due to diagnoses other than CMD in the baseline (2016); Note: Persons entitled to sickness absence benefits are those having an annual income from paid employment of at least 24% of the price base amount of 2016; that is, 10,632 SEK. *Income level categories based on the median of the Swedish working population after applying the inclusion and some of the main exclusion criteria (see methods section). *Income level categories based on the median of the Swedish working population (375,300 SEK) after applying several inclusion and exclusion criteria (see methods section). Income category boundaries: <60% of the median (225,180 SEK), 60-79% of the median (225,180-296,487 SEK), 80-119% of the median (300,240-446,607 SEK), 120-199% of the median (450,360-746,847 SEK), and ≥200% of the median (750,600 SEK). Area of residence and occupation barely changed the estimates; therefore, they were not included in the models as confounders.

For peer review only

STROBE Statement—checklist of items that should be included in reports of observational studies

	Item No.	Recommendation	Page No.	Relevant text from manuscript
**Title and abstract**	1	(*a*) Indicate the study's design with a commonly used term in the title or the abstract	2	
		(*b*) Provide in the abstract an informative and balanced summary of what was done and what was found	2	
**Introduction**				
Background/rationale	2	Explain the scientific background and rationale for the investigation being reported	4	
Objectives	3	State specific objectives, including any prespecified hypotheses	4	
**Methods**				
Study design	4	Present key elements of study design early in the paper	5	
Setting	5	Describe the setting, locations, and relevant dates, including periods of recruitment, exposure, follow-up, and data collection	5	
Participants	6	(*a*) *Cohort study*—Give the eligibility criteria, and the sources and methods of selection of participants. Describe methods of follow-up<br>*Case-control study*—Give the eligibility criteria, and the sources and methods of case ascertainment and control selection. Give the rationale for the choice of cases and controls<br>*Cross-sectional study*—Give the eligibility criteria, and the sources and methods of selection of participants	5	
		(*b*) *Cohort study*—For matched studies, give matching criteria and number of exposed and unexposed<br>*Case-control study*—For matched studies, give matching criteria and the number of controls per case		
Variables	7	Clearly define all outcomes, exposures, predictors, potential confounders, and effect modifiers. Give diagnostic criteria, if applicable	5,6	
Data sources/ measurement	8*	For each variable of interest, give sources of data and details of methods of assessment (measurement). Describe comparability of assessment methods if there is more than one group	5-6	
Bias	9	Describe any efforts to address potential sources of bias	2 (fig. file)	Figure 2
Study size	10	Explain how the study size was arrived at	1 (fig. file)	Figure 1
Continued on next	11	Explain how quantitative variables were handled in the analyses. If applicable, describe which	6	

For peer review only - http://bmjopen.bmj.com/site/about/guidelines.xhtml

page		groupings were chosen and why		
Quantitative variables				
Statistical methods	12	(*a*) Describe all statistical methods, including those used to control for confounding	6	
		(*b*) Describe any methods used to examine subgroups and interactions	6	
		(*c*) Explain how missing data were addressed	N/A	
		(*d*) *Cohort study*—If applicable, explain how loss to follow-up was addressed<br>*Case-control study*—If applicable, explain how matching of cases and controls was addressed<br>*Cross-sectional study*—If applicable, describe analytical methods taking account of sampling strategy	N/A	
		(*e*) Describe any sensitivity analyses	6	
**Results**				
Participants	13*	(a) Report numbers of individuals at each stage of study—eg numbers potentially eligible, examined for eligibility, confirmed eligible, included in the study, completing follow-up, and analysed	1 (fig and supp files)	Figure 1 and Suppl. Table 1
		(b) Give reasons for non-participation at each stage	1 (fig file)	Figure 1
		(c) Consider use of a flow diagram	1 (fig file)	Figure 1
Descriptive data	14*	(a) Give characteristics of study participants (eg demographic, clinical, social) and information on exposures and potential confounders	8	Table 1
		(b) Indicate number of participants with missing data for each variable of interest	8 and 1-4 (supp file)	Footnote Table 1 and Suppl. Tables 1-4 (supp file)
		(c) *Cohort study*—Summarise follow-up time (eg, average and total amount)	N/A	
Outcome data	15*	*Cohort study*—Report numbers of outcome events or summary measures over time	9,11	Tables 2 and 3
		Case-control study—Report numbers in each exposure category, or summary measures of exposure		
		Cross-sectional study—Report numbers of outcome events or summary measures		
Main results	16	(*a*) Give unadjusted estimates and, if applicable, confounder-adjusted estimates and their precision (eg, 95% confidence interval). Make clear which confounders were adjusted for and why they were included	9,11	Tables 2 and 3
		(*b*) Report category boundaries when continuous variables were categorized	8,11 and 2-4 (supp file)	Tables 1,3 and Suppl. Tables 2-4 (supp file)
		(*c*) If relevant, consider translating estimates of relative risk into absolute risk for a meaningful time period	N/A	

For peer review only - http://bmjopen.bmj.com/site/about/guidelines.xhtml

Continued on next page

Other analyses	17	Report other analyses done—eg analyses of subgroups and interactions, and sensitivity analyses	N/A	
**Discussion**				
Key results	18	Summarise key results with reference to study objectives	12	First paragraph of the discussion
Limitations	19	Discuss limitations of the study, taking into account sources of potential bias or imprecision. Discuss both direction and magnitude of any potential bias	13	
Interpretation	20	Give a cautious overall interpretation of results considering objectives, limitations, multiplicity of analyses, results from similar studies, and other relevant evidence	12-13	
Generalisability	21	Discuss the generalisability (external validity) of the study results	14	
**Other information**				
Funding	22	Give the source of funding and the role of the funders for the present study and, if applicable, for the original study on which the present article is based	14	

*Give information separately for cases and controls in case-control studies and, if applicable, for exposed and unexposed groups in cohort and cross-sectional studies.

Note: An Explanation and Elaboration article discusses each checklist item and gives methodological background and published examples of transparent reporting. The STROBE checklist is best used in conjunction with this article (freely available on the Web sites of PLoS Medicine at http://www.plosmedicine.org/, Annals of Internal Medicine at http://www.annals.org/, and Epidemiology at http://www.epidem.com/). Information on the STROBE Initiative is available at www.strobe-statement.org.

For peer review only - http://bmjopen.bmj.com/site/about/guidelines.xhtml

BMJ Open

Unequal access? Use of sickness absence benefits by precariously employed workers with common mental disorders: A register-based cohort study in Sweden

Journal:	*BMJ Open*
Manuscript ID	bmjopen-2023-072459.R2
Article Type:	Original research
Date Submitted by the Author:	29-Jun-2023
Complete List of Authors:	Hernando-Rodriguez, Julio C.; Karolinska Institutet, Unit of Occupational and Environmental Medicine, Institute of Environmental Medicine<br>Matilla-Santander, Nuria; Karolinska Institutet, Unit of Occupational and Environmental Medicine, Institute of Environmental Medicine<br>Murley, Chantelle; Karolinska Institutet, Division of Insurance Medicine, Department of Clinical Neuroscience<br>Blindow, Katrina; Karolinska Institutet, Unit of Occupational and Environmental Medicine, Institute of Environmental Medicine<br>Kvart, Signild; Karolinska Institutet, Unit of Occupational and Environmental Medicine, Institute of Environmental Medicine<br>Almroth, Melody; Karolinska Institutet, Unit of Occupational and Environmental Medicine, Institute of Environmental Medicine; Stockholm University, Department of Public Health Sciences<br>Kreshpaj, Bertina; Karolinska Institutet, Unit of Occupational and Environmental Medicine, Institute of Environmental Medicine; University of Copenhagen, Section of Epidemiology, Department of Publich Health<br>Thern, Emelie; Karolinska Institute, Unit of Occupational Medicine, Institute of Environmental Medicine<br>Badarin, Kathryn; Karolinska Institutet, Unit of Occupational Medicine, Institute of Environmental Medicine<br>Muntaner, Carles; University of Toronto, Lawrence S. Bloomberg Faculty of Nursing; University of Toronto, Dalla Lana School of Public Health<br>Gunn, Virginia; Karolinska Institutet, Unit of Occupational and Environmental Medicine, Institute of Environmental Medicine; St Michael's Hospital Centre for Urban Health Solutions, Li Ka Shing Knowledge Institute, Unity Health Toronto<br>Padrosa, Eva; Consorci Parc de Salut MAR de Barcelona<br>Julià, Mireia; Consorci Parc de Salut MAR de Barcelona<br>Bodin, Theo; Karolinska Institutet, Unit of Occupational and Environmental Medicine, Institute of Environmental Medicine; Centre for Occupational and Environmental Medicine, Stockholm Region
**Primary Subject Heading**:	Occupational and environmental medicine
Secondary Subject Heading:	Public health, Epidemiology
Keywords:	MENTAL HEALTH, EPIDEMIOLOGY, OCCUPATIONAL & INDUSTRIAL MEDICINE, PUBLIC HEALTH

SCHOLARONE™
Manuscripts

I, the Submitting Author has the right to grant and does grant on behalf of all authors of the Work (as defined in the below author licence), an exclusive licence and/or a non-exclusive licence for contributions from authors who are: i) UK Crown employees; ii) where BMJ has agreed a CC-BY licence shall apply, and/or iii) in accordance with the terms applicable for US Federal Government officers or employees acting as part of their official duties; on a worldwide, perpetual, irrevocable, royalty-free basis to BMJ Publishing Group Ltd ("BMJ") its licensees and where the relevant Journal is co-owned by BMJ to the co-owners of the Journal, to publish the Work in this journal and any other BMJ products and to exploit all rights, as set out in our licence.

The Submitting Author accepts and understands that any supply made under these terms is made by BMJ to the Submitting Author unless you are acting as an employee on behalf of your employer or a postgraduate student of an affiliated institution which is paying any applicable article publishing charge ("APC") for Open Access articles. Where the Submitting Author wishes to make the Work available on an Open Access basis (and intends to pay the relevant APC), the terms of reuse of such Open Access shall be governed by a Creative Commons licence – details of these licences and which Creative Commons licence will apply to this Work are set out in our licence referred to above.

Other than as permitted in any relevant BMJ Author's Self Archiving Policies, I confirm this Work has not been accepted for publication elsewhere, is not being considered for publication elsewhere and does not duplicate material already published. I confirm all authors consent to publication of this Work and authorise the granting of this licence.

For peer review only

Unequal access? Use of sickness absence benefits by precariously employed workers with common mental disorders: A register-based cohort study in Sweden

Julio C. Hernando-Rodriguez, PhD[1], Nuria Matilla-Santander, PhD[1], Chantelle Murley, PhD[2], Katrina Blindow, MPH[1], Signild Kvart, MSc[1], Melody Almroth, PhD[1,3], Bertina Kreshpaj, PhD[1,4], Emelie Thern, PhD[1], Kathryn Badarin, PhD[1], Carles Muntaner, PhD[5,6,7], Virginia Gunn, PhD[1,8,9], Eva Padrosa, PhD[,10,11,12], Mireia Julià, PhD[10,11,12], Theo Bodin, PhD[1,13]

[1]Unit of Occupational Medicine, Institute of Environmental Medicine, Karolinska Institutet, Stockholm, Sweden; [2]Division of Insurance Medicine, Department of Clinical Neuroscience, Karolinska Institutet; Stockholm, Sweden; [3]Department of Public Health Sciences, Stockholm University; [4]Section of Epidemiology, Department of Public Health, University of Copenhagen, Copenhagen, Denmark; [5]Lawrence S. Bloomberg Faculty of Nursing, St. George Campus, Toronto, Canada; [6]Dalla Lana School of Public Health, University of Toronto, Toronto, Canada; [7]Department of Mental Health, The Johns Hopkins University Bloomberg School of Public Health, Baltimore, USA; [8]MAP Centre for Urban Health Solutions, Li Ka Shing Knowledge Institute, Unity Health Toronto, Toronto, Canada; [9]School of Nursing, Cape Breton University, Nova Scotia, Canada; [10]ESIMar (Mar Nursing School), Parc de Salut Mar, Universitat Pompeu Fabra-affiliated, Barcelona, Spain; [11]SDHEd (Social Determinants and Health Education Research Group), IMIM (Hospital del Mar Medical Research Institute), Barcelona, Spain; [12]GREDS (Research Group on Health Inequalities, Environment, and Employment Conditions Network), Universitat Pompeu Fabra, Barcelona, Spain; [13]Centre for Occupational and Environmental Medicine, Stockholm Region, Stockholm, Sweden

Corresponding author: Unit of Occupational Medicine, The Institute of Environmental Medicine (IMM), Karolinska Institutet, Solnavägen 4, 113 65, Stockholm, Sweden. julio.hernando@ki.se

Word count text (from introduction to conclusion): 3,938.

Number of tables: 3 (also 5 tables in the supplementary material in another file).

Number of figures: 2 (in another file).

For peer review only

For peer review only - http://bmjopen.bmj.com/site/about/guidelines.xhtml

ABSTRACT

Unequal access? Use of sickness absence benefits by precariously employed workers with common mental disorders: A register-based cohort study in Sweden

Objective

This study compares the use of sickness absence benefits (SABs) due to a common mental disorder (CMD) between precariously employed and non-precariously employed workers with CMDs.

Design

Register-based cohort study.

Participants

The study included 78,215 Swedish workers aged 27-61 who experienced CMDs in 2017, indicated by a new treatment with Selective Serotonin Reuptake Inhibitors (SSRIs). Excluded were those who emigrated or immigrated, were self-employed, had an annual employment-based income < 100 Swedish krona, had > 90 days of unemployment per year, had student status, had SABs due to CMDs during the exposure measurement (2016) and the two previous years, had an SSRI prescription one year or less before the start of the SSRI prescription in 2017, had packs of > 100 pills of SSRI medication, had a disability pension before 2017, were not entitled to SABs due to CMDs in 2016, and had no information about the exposure.

Outcome

The first incidence of SABs due to CMDs in 2017.

Results

The use of SABs due to a CMD was slightly lower among precariously employed workers compared to those in standard employment [adjusted Odds Ratio (aOR) 0.92, 95% confidence interval (CI) 0.81-1.05]. Particularly, women with three consecutive years in precarious employment had reduced SABs use [aOR 0.48, 95% CI 0.26-0.89], while men in precarious employment showed weaker evidence of association. Those in standard employment with high income also showed a lower use of SABs [aOR 0.74, 95% CI 0.67-0.81]. Low unionization and both low and high-income levels were associated with lower use of SABs, particularly among women.

Conclusions

The study indicates that workers with CMDs in precarious employment may use SABs to a lower extent. Accordingly, there is a need for (i) guaranteeing access to SABs for people in precarious employment and/or (ii) reducing involuntary forms of presenteeism.

Key terms: employment quality; precarious employment; SSRI; anxiety; depression; stress-related disorders; mental disorders; mental health; sick leave; welfare state; social insurance system; labour market; epidemiology; Sweden; longitudinal study; presenteeism.

For peer review only - http://bmjopen.bmj.com/site/about/guidelines.xhtml

Strengths and limitations of this study:

- This study utilizes population registers with high validity and low attrition rates.

- The linkage of work disability and pharmaceutical registers enables accurate measurement of sickness absence in individuals in formal employment receiving new CMDs treatment.

- The longitudinal design of this study minimizes the potential for reverse causation.

- Potential underestimation of findings by requiring three years of exposure to precarious employment (those with longer exposure tend to be healthier) is mitigated in the one-year analysis.

For peer review only

For peer review only - http://bmjopen.bmj.com/site/about/guidelines.xhtml

BACKGROUND

In the past decades, many countries, including Sweden, have experienced changes in labour market policies that prioritize organizational flexibility over employment security [1]. This led to an increase in precarious employment (PE) [2], which is characterized by employment insecurity, insufficient income, and a lack of rights and social protection [3,4]. The impact of these labour market changes on the health of the workforce and the use of sickness absence benefits (SABs) is not fully understood, especially, among workers in PE.

Mental disorders are one of the major causes of the global burden of disease [5]. In Sweden, half of the working-age population experiences a mental health condition during their lifetime; every year, 1 million workers have a mental disorder [6]. As a result, mental disorders are one of the leading diagnoses causing sickness absence (SA), especially among young people [7]. SA rates are not only driven by the health and work capacity of the working population but are influenced by contextual factors. Therefore, it has been suggested that low rates of SA in insecure jobs may reflect higher levels of presenteeism (i.e., working while being ill) due to workers' fear of dismissal rather than better health status [8,9].

In Sweden, the proportion of the working-age population who are at risk of not being entitled to SA is among the lowest (less than 1%) in the European Union [10]. Those who are not entitled to SA are often family workers employed in small family businesses and who do not receive a formal salary and, therefore, do not pay social contributions [10]. However, in 2016, the Swedish Social Insurance Inspectorate (ISF) deemed temporary employees' access to SABs to be legally uncertain, mainly due to the design of regulations that leave room for interpretation [11]. Workers without fixed schedules are particularly affected because this causes ambiguity as to what hours they would have worked had they not been sick, which opens the possibility to consider non-working periods as unemployment. The regulations of SABs are different for employees and unemployed persons, including entitlement requirements and the level of compensation. Moreover, the ISF found that temporary employees had fewer days of SABs than those permanently employed.

Previous studies have examined demographic characteristics such as age and gender, singular employment characteristics, trade union membership or work environment factors in relation to SA [12–15]. Some of these determinants of SA have been examined specifically among people with common mental disorders (CMDs – stress-related disorders, depression, and anxiety) [16]. To our knowledge, only two previous cross-sectional studies have analysed the relationship between PE and SA, and both of which found a positive association [17,18]. This is in line with previous studies confirming a relationship between PE and poor health, including a systematic review of longitudinal studies [19]. A recent Swedish study using the same data sources as the present study found that trajectories with constant PE increased the risk of CMDs [20].

Accordingly, we hypothesise that workers in PE would have a higher likelihood of using SABs due to CMDs when compared to those in standard employment due to their worse mental health status. However, workers in PE who formally qualify for SABs may still experience barriers to accessing their rights. To investigate the use of SABs, we include only individuals who have recently initiated treatment for CMDs and who formally qualify for SABs.

This study aims to compare the use of SABs due to CMDs between precariously employed and non-precariously employed workers with CMDs.

For peer review only - http://bmjopen.bmj.com/site/about/guidelines.xhtml

METHODS

Study population

This register-based study is based on the Swedish Work, Illness, and Labour-market Participation (SWIP) cohort of the total Swedish population. The SWIP cohort is obtained through the linkage of multiple registers and includes all individuals aged 16-65 years who were registered in Sweden in 2005 (around 5.7 million individuals), with follow-up until 2017. This study uses a subpopulation of 78,215 individuals aged 27-61 years in 2016 who experienced a CMD in 2017, indicated by the initiation of treatment with Selective Serotonin Reuptake Inhibitors (SSRIs). Included individuals had similar characteristics to the Swedish workforce, except for the higher share of women (Supplementary Table 1).

Sociodemographic and employment data were obtained from the Longitudinal Integration Database for Health Insurance and Labour Market Studies (LISA) for 2016. Data on SA (from day 15 onwards) were taken from the Micro Data for Analysis of Social Insurance (MiDAS) registers and included information such as ICD-10 diagnosis and length of SA for 2017. The Swedish Prescribed Drug Register informed the prescribed medicines dispensed from pharmacies for 2017.

Exclusion criteria were: (i) emigration or immigration (2012-2016), (ii) self-employment during 2014-2017 because SABs regulations differ [21], (iii) annual employment-based income < 100 SEK to ensure working status, (iv) > 90 days of unemployment per year to avoid measuring the effect of unemployment on the outcome, (v) student status during the autumn term to avoid misclassification as PE, (vi) SA due to CMDs during the exposure measurement (2016) and the two previous years (2014-2015) to reduce the risk of reverse causation, (vii) SSRI prescription one year or less before the start of SSRI prescription in 2017 [22,23], (viii) packs of > 100 pills of SSRI medication in 2017, as this is uncommon for new SSRI treatment, (ix) disability pension before 2017, (x) no entitlement to SABs due to CMDs in 2016, and (xi) incomplete information on the exposure variable in 2016 (2014-2016 in the sensitivity analysis) (Figure 1).

Exposure variable

The PE measure was based on the second version of the Swedish Register-based Operationalization of Precarious Employment (SWE-ROPE) [24]. The SWE-ROPE considers five items (i.e., contractual relationship insecurity, contractual temporariness, multiple jobs/economic sectors, income level and lack of unionization) that represent the three dimensions of PE (i.e., employment insecurity, income inadequacy and lack of rights and protection). For each individual, an annual score for each of the five items was calculated [25]. Scores ranged by item: contractual employment insecurity (agency employed to directly employed: from -1 and 0); contractual temporariness (unstable employment and stable employment: -2 and 0); multiple jobs/economic sectors (multiple jobs and sectors, multiple jobs, a single job: -2 to 0), income level (< 60% of the median, 60-79%, 80-119%, 120-200% and > 200%: -2 to +2) and lack of unionization (< 70% of collective bargaining agreement coverage, 70-90% and > 90%: -2 to 0). The median income (375,300 SEK in 2016) was based on the Swedish working population after applying the inclusion and some of the main exclusion criteria (i to vii and xi described above).

A final summative score was obtained by summing the total score of the five items. Lower final scores suggest PE, while a higher score suggests non-PE. The final summative score for 2016 was categorised into four employment quality typologies: precarious employment relationship (PER;

For peer review only - http://bmjopen.bmj.com/site/about/guidelines.xhtml

score<-3), sub-standard employment relationship (SSER; a score between -3 and -1), standard employment relationship (SER; score=0) and standard employment relationship with high income (SER/HI; score>0). The SER/HI group was defined in light of previous literature [20,26] and included more than 85% of individuals with high-income levels (120-199% of the median income).

Outcome variable

The outcome was the first occurrence of SABs due to CMDs starting in the 2 months spanning the month before and after the SSRI prescription date in 2017 (categorical outcome: 0, having SSRI and no SABs; 1, having SSRI co-occurring with SABs). This period was selected based on literature observing the effect of SSRI treatment after 3-4 weeks, so the risk of experiencing a new SA spell after that time may decrease [27–29]. The SSRI prescriptions included antidepressants from N06AB02 to N06AB10 according to the Anatomical Therapeutic Chemical (ATC) classification system. SA due to CMDs was identified by ICD-10 codes: depression (F32-F33), anxiety (F41) and stress-related disorders (F43) including exhaustion disorder, which is conceptually similar to burnout. This definition of CMDs is based on a previous paper about PE and the risk of CMDs [20]. Regarding the SABs, all people living in Sweden aged 16 and over who receive a minimum annual income from work of at least 24% of the price base amount (10,752 SEK in 2017) and who have a reduced work capacity of at least 25% of their usual working hours due to disease or injury are covered by the SA insurance system [30]. A physician's certificate is required after the first seven days of SA. SABs replace up to 80% of lost work income. After the first qualifying day without compensation, the employer pays SA for the first 13 days. Therefore, SA spells of less than 15 days are not registered in MiDAS. From the 15th day onwards, SABs are paid by the Social Insurance Agency. For the present study, SABs due to CMDs included only SA spells of at least 15 days.

Potential confounding variables

At baseline, age, education level, country of birth, family composition (civil status and having children), unemployment duration, economic sector, area of residence, occupation, and comorbidity (2016). Comorbidity was measured as having SA due to any diagnoses other than CMDs since SA episodes in the previous years showed to be a predictor for future episodes of SA [31].

Statistical analysis

Logistic regression models estimated the association between employment quality typologies and the use of SABs due to CMDs in 2017. A sensitivity analysis was conducted by conditioning models to have the same exposure for three consecutive years (2014-2016) to observe if stability could influence the results. Models were adjusted for the potential confounders mentioned above and identified using a Directed Acyclic Graph (DAG) (Figure 2). All analyses were conducted for the total population as well as women and men separately. The analysis is separated by gender because previous studies have found different patterns in SA among women and men [32,33]. Women have higher SA rates compared to men possibly due to the higher exposure to an unequal division of domestic work combined with paid work [32]. Work-family conflicts are related to higher ORs to report short SA (1-7 days) among men and longer SA among women (8-30 days) [33]. Interaction effects of gender and the main exposure were tested. We would like to clarity our approach to interpreting the results of the models by considering confidence intervals as "compatibility intervals" as recommended by Amrhein et al.

For peer review only - http://bmjopen.bmj.com/site/about/guidelines.xhtml

[34]. Rather than solely focusing on statistical significance, we examined the effect sizes that are most compatible with our data.

Data management and statistical analysis were performed using Stata SE v17.

Patient and public involvement

Patients and/or the public were not involved in the design, or conduct, or reporting, or dissemination plans of this research.

RESULTS

People in PER (5.10%) had a higher proportion of young-middle aged individuals (27-40 years) (59.4%), women (69.7%), low educational attainment (63.2%), born in Sweden (88.4%), couples with children (44.4%), zero days unemployed (85.6%), private sector (79.6%), unstable employment (93.5%), multiple job holding in different economic sectors (20.7%), income level below 60% of the median (47.5%), and low unionization (22.6%) (Table 1). The proportion of people having a first spell of SABs due to CMDs in 2017 was 7.5% among our study population and showed differences according to sociodemographic and employment characteristics in general (Supplementary Table 2), and by gender and employment quality typology (Supplementary Tables 3 and 4, respectively).

For peer review only - http://bmjopen.bmj.com/site/about/guidelines.xhtml

Table 1. Sociodemographic and employment characteristics at baseline (2016) in a cohort of 78,215 individuals with common mental disorders in 2017, aged 27-61, entitled to sickness absence benefits in 2016 by employment quality typology (Standard Employment Relationship, SER; Standard Employment Relationship with high income, SER/HI; Sub-standard Employment Relationship, SSER; Precarious Employment Relationship, PER; baseline, 2016).

	SER/HI (14.68%)	SER (38.04%)	SSER (42.18%)	PER (5,10%)
	N (%)	N (%)	N (%)	N (%)
**Age**				
27-40 years	2,878 (25.1)	9,817 (33.0)	15,612 (47.3)	2,368 (59.4)
41-51 years	5,239 (45.6)	11,432 (38.4)	10,844 (32.9)	1,026 (25.7)
52-61 years	3,366 (29.3)	8,504 (28.6)	6,537 (19.8)	592 (14.9)
**Gender**				
Male	5,999 (52.2)	10,133 (34.1)	8,554 (25.9)	1,208 (30.3)
Female	5,484 (47.8)	19,620 (65.9)	24,439 (74.1)	2,778 (69.7)
**Education level**				
Elementary/high school education	3,395 (29.6)	15,831 (53.2)	19,329 (58.6)	2,517 (63.2)
Higher education <3 years	1,989 (17.3)	4,384 (14.7)	4,446 (13.5)	637 (16.0)
Higher education ≥3 years	6,092 (53.1)	9,528 (32.0)	9,201 (27.9)	830 (20.8)
**Country of birth**				
Sweden	10,449 (91.0)	26,561 (89.3)	29,203 (88.6)	3,523 (88.4)
Not Sweden	1,031 (9.0)	3,174 (10.7)	3,770 (11.4)	463 (11.6)
**Family composition**				
Couple with children	6,045 (52.6)	13,320 (44.8)	16,338 (49.5)	1,768 (44.4)
Couple without children	1,354 (11.8)	3,630 (12.2)	3,151 (9.6)	286 (7.2)
Single with children	1,252 (10.9)	3,903 (13.1)	4,528 (13.7)	560 (14.0)
Single without children	2,832 (24.7)	8,900 (29.9)	8,976 (27.2)	1,372 (34.4)
**Unemployment**				
0 days unemployed	11,429 (99.5)	29,514 (99.2)	31,733 (96.2)	3,414 (85.6)
From 1 to 90 days unemployed	54 (0.5)	239 (0.8)	1260 (3.8)	572 (14.4)
**Economic sector**				
Private	6,995 (60.9)	13,908 (46.7)	17,578 (53.3)	3,172 (79.6)
Public	4,488 (39.1)	15,845 (53.3)	15,415 (46.7)	814 (20.4)
**Contractual relationship insecurity**				
Directly employed by an employer	11,473 (99.9)	29,715 (99.9)	32,529 (98.6)	3,765 (94.5)
Employed by an agency	10 (0.1)	38 (0.1)	464 (1.4)	221 (5.5)
**Contractual temporariness**				
Stable employment	11,483 (100.0)	29,282 (98.4)	18,070 (54.8)	260 (6.5)
Unstable employment	(0.0)	471 (1.6)	14,923 (45.2)	3,726 (93.5)
**Multiple jobs/economic sectors**				
1-2 jobs	11,143 (97.0)	29,123 (97.9)	30,584 (92.7)	2,168 (54.4)
3 or more jobs	221 (1.9)	440 (1.5)	1,916 (5.8)	994 (24.9)
3 or more jobs in 3 or more sectors	119 (1.0)	190 (0.6)	493 (1.5)	824 (20.7)
**Income level***				
<60% of the median	(0.0)	(0.0)	3,320 (10.1)	1,892 (47.5)
60-79% of the median	(0.0)	(0.0)	14,823 (44.9)	1,153 (28.9)
80-119% of the median	(0.0)	28,657 (96.3)	10,708 (32.5)	878 (22.0)
120-200% of the median	9,849 (85.8)	590 (2.0)	3,960 (12.0)	62 (1.6)
>200% of the median	1,634 (14.2)	506 (1.7)	182 (0.6)	1 (<0.1)
**Unionization level**				
<70%	(0.0)	20 (0.1)	852 (2.6)	901 (22.6)
70-90%	55 (0.5)	412 (1.4)	3,297 (10.0)	866 (21.7)
>90%	11,428 (99.5)	29,321 (98.5)	28,844 (87.4)	2,219 (55.7)

Missing values in the educational level (SER/HI: 7, 0.06%; SER: 10, 0.03%; SSER: 17, 0.05%; PE: 2, 0.05%) and country of birth (SER/HI: 3, 0.03%; SER: 18, 0.06%; SSER: 20, 0.06). Persons entitled to sickness absence benefits in 2016 are those having an annual income from paid employment of at least 24% of the price base amount of 2016; that is, 10,632 SEK. *Income level categories based on the median of the Swedish working population (375,300 SEK) after applying several inclusion and exclusion criteria (see methods section). Income category boundaries: <60% of the median (225,180 SEK), 60-79% of the median (225,180-296,487 SEK), 80-119% of the median (300,240-446,607 SEK), 120-199% of the median (450,360-746,847 SEK), and ≥200% of the median (750,600 SEK).

For peer review only - http://bmjopen.bmj.com/site/about/guidelines.xhtml
For peer review only

The association between 1-year exposure to employment quality typologies and the use of SABs due to CMDs exhibited an inverted U-shape pattern, with lower adjusted Odds Ratio (aOR) estimates found among individuals with an SER/HI (aOR: 0.74, 95% CI: 0.67-0.81), followed by near-significance estimates of those in a PER, compared to the SER group (aOR: 0.92, 95% CI: 0.81-1.05) (Table 2). This association between PER and SABs suggests 8% reduced risk. However, our data is also compatible with a range of risk differences, spanning from a reduction of 19% to a small increase of 5%. The SSER group showed no significant differences compared to the SER group. Women were less likely to use SABs due to CMDs in the PER (OR: 0.88, 95% CI: 0.75-1.04) and the SER/HI groups (aOR: 0.72, 95% CI: 0.63-0.83) compared to the SER group. Men in SSER and PER showed weaker evidence of association, except for those in the SER/HI group (aOR: 0.76, 95% CI: 0.66-0.86). Overall, regression models conditional on having the same exposure for three consecutive years showed similar results, in this case, lower use of SABs due to CMDs occurred among individuals with SER/HI (aOR: 0.74, 95% CI: 0.65-0.85). Women with three consecutive years of PER had the lowest probability of using SABs due to CMDs (aOR: 0.48, 95% CI: 0.26-0.89), followed by women and men with SER/HI compared to the SER group (aOR: 0.76, 95% CI: 0.62-0.92 and aOR: 0.73, 95% CI: 0.61-0.88, respectively). Given that area of residence and occupation barely changed the estimates, they were not included in the models as confounders.

Table 2. Association between employment quality typologies (Standard Employment Relationship, SER; Standard Employment Relationship with high income, SER/HI; Sub-standard Employment Relationship, SSER; Precarious Employment Relationship, PER; baseline, 2016) and sickness absence benefits due to common mental disorders in 2017 among people entitled to sickness absence benefits in 2016 (N=78,215).

					Models with three years of continuous exposure* (N=38,761)			
	N	Cases N (%)	OR (95% CI)	aOR (95% CI)	N	Cases N (%)	OR (95% CI)	aOR (95% CI)
**Employment quality typology**								
SER	29,753	2,268 (7.6)	1	1	17,353	1,207 (7.0)	1	1
SER/HI	11,483	593 (5.2)	0.66 (0.60-0.72)	0.74 (0.67-0.81)	6,924	330 (4.8)	0.67 (0.59-0.76)	0.74 (0.65-0.85)
SSER	32,993	2,667 (8.1)	1.07 (1.01-1.13)	0.99 (0.93-1.05)	14,065	1,055 (7.5)	1.08 (1.00-1.18)	1.01 (0.92-1.10)
PER	3,986	320 (8.0)	1.06 (0.94-1.20)	0.92 (0.81-1.05)	419	24 (5.7)	0.81 (0.54-1.23)	0.74 (0.48-1.12)
**Women (N=52,321)**								
SER	19,620	1,375 (7.0)	1	1	11,137	715 (6.4)	1	1
SER/HI	5,484	247 (4.5)	0.63 (0.54-0.72)	0.72 (0.63-0.83)	2,954	129 (4.4)	0.67 (0.55-0.81)	0.76 (0.62-0.92)
SSER	24,439	1,892 (7.7)	1.11 (1.04-1.20)	1.00 (0.93-1.08)	11,138	810 (7.3)	1.14 (1.03-1.27)	1.00 (0.90-1.12)
PER	2,778	198 (7.1)	1.02 (0.87-1.19)	0.88 (0.75-1.04)	296	11 (3.7)	0.56 (0.31-1.03)	0.48 (0.26-0.89)
**Men (N=25,894)**								
SER	10,133	893 (8.8)	1	1	6,216	492 (7.9)	1	1
SER/HI	5,999	346 (5.8)	0.63 (0.56-0.72)	0.76 (0.66-0.86)	3,970	201 (5.1)	0.62 (0.52-0.73)	0.73 (0.61-0.88)
SSER	8,554	775 (9.1)	1.03 (0.93-1.14)	0.97 (0.88-1.08)	2,927	245 (8.4)	1.06 (0.91-1.25)	1.01 (0.86-1.19)
PER	1,208	122 (10.1)	1.16 (0.95-1.42)	1.02 (0.83-1.25)	123	13 (10.6)	1.37 (0.77-2.46)	1.31 (0.73-2.36)

OR: unadjusted Odds Ratio; aOR: Odds Ratio adjusted by age, gender, education, country of birth, family composition, economic sector, unemployment, and SA due to diagnoses other than CMD in the baseline (2016); *Models conditioned to have the same exposure for three consecutive years (2014-2016). Note: Persons entitled to sickness absence benefits are those having an annual income from paid employment of at least 24% of the price base amount of 2016; that is, 10,632 SEK. Area of residence and occupation barely changed the estimates; therefore, they were not included in the models as confounders.

For peer review only - http://bmjopen.bmj.com/site/about/guidelines.xhtml

We tested interaction effects between gender and employment quality. Women in precarious employment had a 27% lower risk of sickness absence benefits compared to men in standard employment after controlling for the same potential confounding factors (data not shown). The effect of the interaction term was somewhat pronounced for women in PER, but, in general, differences between gender and employment quality categories were relatively small. However, when comparing the model without the interaction term to the model containing it, the likelihood ratio test was not statistically significant (p-value>0.3955) (data not shown). This suggests that including the interaction term does not improve the fit of the model. Therefore, we have kept the original model without the interaction term.

The employment quality items most strongly related to the use of SABs due to CMDs were income and unionization levels (Table 3). In the fully adjusted model, high income was associated with a reduced probability of SABs due to CMDs (120-199% of the median income - aOR: 0.79, 95% CI: 0.72-0.85; and more than 199% of the median income - aOR: 0.61, 95% CI: 0.49-0.75), while the lowest-income level showed slight differences (lower than 60% of the median income – aOR: 0.92, 95% CI: 0.82-1.03). Women with the highest-income levels exhibit lower aORs compared to men (aOR: 0.48, 95% CI: 0.33-0.71 and aOR: 0.71, 95% CI: 0.54-0.92, respectively). Low unionization coverage was associated with lower use of SABs (less than 70% of unionization level - aOR: 0.82, 95% CI: 0.68-0.99), particularly among women (0.77, 95% CI: 0.58-1.03, near to significance). Similar results were found for women and men with medium unionization coverage. The unadjusted ORs and two different model adjustments can be observed in Supplementary Table 5.

For peer review only - http://bmjopen.bmj.com/site/about/guidelines.xhtml

Table 3. Association between employment quality dimensions (2016, baseline) and sickness absence benefits due to common mental disorders in 2017 (N=78,215) among people entitled to sickness absence benefits in 2016.

			Women (N=52,321)		Men (N=25,894)	
	Cases N (%)	aOR (95% CI)	Cases N (%)	aOR (95% CI)	Cases N (%)	aOR (95% CI)
**Contractual relationship insecurity**						
Directly employed by employer	5,788 (7.5)	1	3,683 (7.1)	1	2,105 (8.2)	1
Employed by an agency	60 (8.2)	0.96 (0.73-1.25)	29 (6.5)	0.85 (0.58-1.24)	31 (10.9)	1.08 (0.74-1.58)
**Contractual temporariness**						
Stable employment	4,351 (7.4)	1	2,792 (7.1)	1	1,559 (8.0)	1
Unstable employment	1,497 (7.8)	1.02 (0.95-1.09)	920 (7.2)	1.01 (0.92-1.10)	577 (9.0)	1.04 (0.93-1.16)
**Multiple jobs/economic sectors**						
1-2 jobs	5,436 (7.4)	1	3,463 (7.1)	1	1,973 (8.2)	1
3 or more jobs	279 (7.8)	1.05 (0.93-1.20)	175 (7.0)	1.00 (0.85-1.18)	104 (9.6)	1.18 (0.95-1.46)
3 or more jobs in 3 or more sectors	133 (8.2)	1.09 (0.91-1.31)	74 (7.3)	1.04 (0.81-1.32)	59 (9.6)	1.19 (0.90-1.57)
**Income level**						
80-119% of the median	3,113 (7.7)	1	1,876 (7.1)	1	1,237 (8.9)	1
<60% of the median	395 (7.6)	0.92 (0.82-1.03)	319 (7.3)	0.91 (0.81-1.04)	76 (8.9)	0.94 (0.73-1.20)
60-79% of the median	1,415 (8.9)	1.10 (1.03-1.18)	1,111 (8.4)	1.08 (1.00-1.17)	304 (10.9)	1.16 (1.01-1.33)
120-200% of the median	831 (5.8)	0.79 (0.72-0.85)	378 (5.1)	0.79 (0.70-0.88)	453 (6.4)	0.80 (0.71-0.89)
>200% of the median	94 (4.1)	0.61 (0.49-0.75)	28 (2.9)	0.48 (0.33-0.71)	66 (4.9)	0.71 (0.54-0.92)
**Unionization level**						
>90%	5,409 (7.5)	1	3,497 (7.2)	1	1,912 (8.3)	1
70-90%	315 (6.8)	0.82 (0.73-0.92)	161 (6.1)	0.83 (0.70-0.98)	154 (7.7)	0.80 (0.67-0.96)
<70%	124 (7.0)	0.82 (0.68-0.99)	54 (5.8)	0.77 (0.58-1.03)	70 (8.2)	0.84 (0.65-1.09)

aOR: Odds Ratio adjusted mutually by the other employment quality dimensions and age, gender, education, country of birth, family composition, economic sector, unemployment, and SA due to diagnoses other than CMD in the baseline (2016); Note: Persons entitled to sickness absence benefits are those having an annual income from paid employment of at least 24% of the price base amount of 2016; that is, 10,632 SEK. *Income level categories based on the median of the Swedish working population after applying the inclusion and some of the main exclusion criteria (see methods section). *Income level categories based on the median of the Swedish working population (375,300 SEK) after applying several inclusion and exclusion criteria (see methods section). Income category boundaries: <60% of the median (225,180 SEK), 60-79% of the median (225,180-296,487 SEK), 80-119% of the median (300,240-446,607 SEK), 120-199% of the median (450,360-746,847 SEK), and ≥200% of the median (750,600 SEK). Area of residence and occupation barely changed the estimates; therefore, they were not included in the models as confounders.

For peer review only - http://bmjopen.bmj.com/site/about/guidelines.xhtml

DISCUSSION

This study indicated that having a PER was associated with a lower probability of using SABs due to CMDs compared to SER, especially among women. On the other side of the spectrum, we also found that SER/HI was strongly and negatively associated with SABs. The income and unionization level were the themes of employment quality most strongly related to using SABs due to CMDs: having high income and low unionization levels were associated with lower use of SABs due to CMDs, particularly, among women.

Our results on the negative association between PER and the use of SABs due to CMDs contrast with previous studies that found an increased prevalence of self-reported SA due to health problems among precariously employed workers, in Sweden and other countries [17,18]. In our present study, sickness presenteeism (SP) may explain the lower odds of SABs due to CMDs in the PER group. Precariously employed workers who typically have low-income jobs are likely to incur SP because they cannot afford to use SABs. Using SABs results in a period of reduced income since the SABs only partially replace wages [35]. Additionally, SP may stem from the fear of being dismissed due to employment insecurity or from the lack of union protection [9,36]. An earlier study found that workers who transitioned from temporary to permanent employment had an increased probability of SA [8]. Precariously employed workers in our study population had the lowest unionization levels and low unionization was related to lower use of SABs due to CMDs. These findings are consistent with previous studies in Germany and Norway where trade union members had higher SA than non-members [14,15]. Furthermore, sickness presenteeism can be partially explained by the social stigma surrounding mental health, which may originate from colleagues and employers [37]. This stigma can lead individuals to feel more validated in taking sickness absence when their illness is physical, rather than when it is related to mental health. A second possible explanation for the low use of SABs among precariously employed workers is related to legal uncertainty when the Social Insurance Agency assesses entitlement to SABs for non-standard employees [38]. If temporary employees have scheduled work, they receive SABs for the days that they would have worked had the illness not reduced their work capacity by at least 25%. Non-standard employees however often lack a formal schedule covering the days in question, e.g., on-demand employees get their shifts assigned on a day-to-day basis or have a schedule based on an oral agreement, and risk being considered unemployed in the event of sickness and cannot access SABs from the employer. To access SABs, work capacity must be assessed, and this is done differently according to employment status. The work capacity of employees is assessed only relative to their current employment for the first 90 days of SA and only later against all jobs in the entire labour market, but unemployed (or non-standard workers without scheduled hours) will be assessed relative to all jobs in the entire labour market from the start. In practice, this often means that they do not qualify for SABs, since other jobs may exist in the labour market where their current functional ability does not impose a sufficient reduction in work capacity. However, we may bear in mind that this explanation is less plausible for workers with CMDs that may have a reduced work capacity for any task.

In general, women had lower use of SABs in any employment quality typology compared to the SER, and the association was more pronounced in women than in men. These results were unexpected since women usually have higher SA rates than men [39]. However, our study population only included individuals with SSRI treatment (two-thirds of them women), which has not been studied before with this study design [40]. The lower use of SABs among women is plausible because it has been suggested that women may feel a greater responsibility to their

For peer review only - http://bmjopen.bmj.com/site/about/guidelines.xhtml

colleagues and avoid burdening them by cooperating more when performing work tasks compared to men [35]. Furthermore, another study showed that female-dominated jobs related to jobs with responsibility for others such as education, human health and social work activities are more prone to SP [41]. However, this explanation is unlikely to apply to our study since we accounted for the potential effect of women's concentration in certain sectors on SABs by controlling for the sector in the analysis. Women with a consecutive 3-year period in PER had the lowest use of SABs. This could be explained by the fact that women are more frequently employed in temporary employment, which has been related to fewer days of SABs than permanently employed and, probably, to lower access to SABs for the reasons previously mentioned [38].

SER/HI showed the highest income levels and we found that having a high income was related to a lower likelihood of using SABs. In this line, a Finnish study found that a higher position by education, occupational class and income was related to lower SA [42]. Another possible explanation for the lower use of SABs in employees in SER/HI is also SP, but for different reasons than the PER group. In a study conducted in Norway and Sweden, having supervisory responsibilities at work that no one else can perform was an influential factor for SP, which could be the case for the high earners in the present study [35]. Another driver for SP among high earners could be work satisfaction since they probably have jobs with more creativity, freedom and control [35,43]. Another explanation is that high earners have more knowledge-intensive occupations where they can work from home rather than take SA [44]. Lastly, individuals in SER/HI could plausibly be more likely to combine SSRIs with cognitive behavioural therapy (CBT) or mindfulness which could be, to a certain extent, protective of work capacity [45,46].

SA can also be conceptualised as a health status measure. Accordingly, an important consequence of SP is an increased risk of more severe and long-lasting health problems that, in turn, lead to longer periods of SA, regardless of the employment quality typology followed [47].

LIMITATIONS AND STRENGTHS

Limitations of this study could arise from the design of the study when analysing individuals with three years with the same exposure since those with PE are healthier enough to not exit the labour market, which could underestimate the results. However, we also analysed one year of exposure before SABs, finding comparable results which may overcome the previous limitation. Another limitation is the possibility of residual confounding when inferring the impact of employment quality on SABs. We cannot rule out that some people in the study population were not entitled to SABs since apart from the prior income requirement, it is necessary to have a reduced work capacity due to the CMD of at least 25%, which is not possible to assess in the datasets. Selection bias could be introduced among SER/HI because CBT and other psychotherapies could be more prevalent in competing with SSRIs. It could be thought that we could have included a longer period for outcome measurement. However, our main interest is using SSRIs as an indicator of having a new diagnosis of CMDs and our approach is conservative in considering the occurrence of SABs close to the prescription of SSRIs since there is mixed evidence about the long-term efficacy of SSRIs [29,48]. Another limitation is that we did not have access to a level of detail of work to explore whether a person in non-standard arrangements is scheduled for work or is between work periods. Further research with alternative data sources is needed in this regard.

For peer review only - http://bmjopen.bmj.com/site/about/guidelines.xhtml

One of the strengths of this study is that it is based on population registers with high validity and low attrition rates, which allows for accurate and objective measurement of PE through a multi-dimensional measure [49]. Similarly, the linkage of pharmaceutical and work disability registers with the exact dates of SSRI prescription and SABs allowed for an accurate assessment of the outcome. The study is based on a large subpopulation of the Swedish working population. Lastly, the longitudinal study design reduces reverse causation (i.e., health selection into precarious employment).

GENERALIZABILITY

The results of this study are specific to our Swedish study population with a prescription of SSRIs and may not be generalisable to other countries with large variations in the treatments available for CMDs, subsidisation of SSRIs costs, and SABs regulations. Additionally, it is important to note that this study specifically focuses on the effects of PER on the onset of CMDs, and it is possible that the onset of other health conditions, particularly those that are physically related (not subjected to stigma as mental health), may lead to different patterns of SABs.

CONCLUDING REMARKS

This study observed a trend of lower use of SABs among people with CMDs in PER and SER/HI than in SER, particularly, women with a period of three consecutive years in PER. Accordingly our findings suggest that precariously employed workers may face barriers to accessing SABs. More inclusive policies are recommended to reduce these obstacles and guarantee SABs for those who are in PER and/or reduce involuntary forms of SP.

Contributors

TB conceived the initial idea of the study and act as a guarantor of the work. All authors contributed to the design of the study. TB and JCH-R further developed the study design and discussed it with NM-S. Data management and data analysis were performed by JCH-R with support from NM-S. All authors contributed to the interpretation of the results. JCH-R drafted the first version of the manuscript, and all authors made subsequent revisions of the work, agreed on the text and findings, and approved the final version. The corresponding author certifies that all listed authors meet authorship criteria.

Funding

This research was funded by FORTE—Swedish Research Council for Health Working Life and Welfare. Grant number 2021-00034.

Acknowledgements

The authors are grateful to all the researchers of the PWR Consortium and acknowledge Anette Linnersjö for ordering and receiving data and the valuable input on the data management. The authors also acknowledge feedback provided by Cecilia Orellana and Fabrizio Méndez Rivero during various stages of the development of the study.

Competing interests

The authors declare no conflict of interest. The funder had no role in the design of the study; in the collection, analysis, or interpretation of data; in the writing of the manuscript, or in the decision to publish the results.

For peer review only - http://bmjopen.bmj.com/site/about/guidelines.xhtml

Patient and public involvement

Patients and/or the public were not involved in the design, or conduct, or reporting, or dissemination plans of this research.

Patient consent for publication

Not required.

Ethics approval

The Regional Ethics Board of Stockholm granted ethical permission for the study (no. 2017/1224-31/2 and 2018/1675-32). This is a register-based study and informed consent is not applicable.

Data availability statement

Data may be obtained from a third party and are not publicly available.

REFERENCES

1 Rodgers G. Labour Market Flexibility and Decent Work. 2007;:11.

2 Berglund T, Håkansson K, Isidorsson T. Occupational change on the dualised Swedish labour market. *Econ Ind Democr* 2022;**43**:918–42. doi:10.1177/0143831X20959714

3 Kreshpaj B, Orellana C, Burström B, *et al.* What is precarious employment? A systematic review of definitions and operationalizations from quantitative and qualitative studies. *Scand J Work Environ Health* 2020;**46**:235–47. doi:10.5271/sjweh.3875

4 Benach J, Muntaner C. Precarious employment and health: developing a research agenda. *J Epidemiol Community Health* 2007;**61**:276–7. doi:10.1136/jech.2005.045237

5 Global, regional, and national burden of 12 mental disorders in 204 countries and territories, 1990–2019: a systematic analysis for the Global Burden of Disease Study 2019. *Lancet Psychiatry* 2022;**9**:137–50. doi:10/gn7nxg

6 OECD. Mental Health and Work: Sweden. 2013. https://doi.org/10.1787/9789264188730-en (accessed 12 Jul 2022).

7 Swedish Social Insurance Agency [Försäkringskassan]. Social Insurance in Figures 2021. 2021.

8 Virtanen M, Kivimäki M, Elovainio M, *et al.* From insecure to secure employment: changes in work, health, health related behaviours, and sickness absence. *Occup Environ Med* 2003;**60**:948–53. doi:10.1136/oem.60.12.948

9 Johns G. Presenteeism in the workplace: A review and research agenda. *J Organ Behav* 2010;**31**:519–42. doi:10.1002/job.630

10 Matsaganis M, Özdemir E, Ward T, *et al.* Non-standard employment and access to social security benefits. 2015;:43.

For peer review only - http://bmjopen.bmj.com/site/about/guidelines.xhtml

11 Schill HP, Bjurström S, Pietrek J, *et al.* The Swedish sickness insurance and temporary employment. 2016;:5.

12 Allebeck P, Mastekaasa A. Chapter 5. Risk factors for sick leave - general studies. *Scand J Public Health* 2004;**32**:49–108. doi:10/dvr35c

13 Virtanen M, Kivimaki M, Joensuu M, *et al.* Temporary employment and health: a review. *Int J Epidemiol* 2005;**34**:610–22. doi:10.1093/ije/dyi024

14 Mastekaasa A. Unionization and Certified Sickness Absence: Norwegian Evidence. *ILR Rev* 2013;**66**:117–41. doi:10.1177/001979391306600105

15 Goerke L, Pannenberg M. Trade union membership and sickness absence: Evidence from a sick pay reform. *Labour Econ* 2015;**33**:13–25. doi:10.1016/j.labeco.2015.02.004

16 de Vries H, Fishta A, Weikert B, *et al.* Determinants of Sickness Absence and Return to Work Among Employees with Common Mental Disorders: A Scoping Review. *J Occup Rehabil* 2018;**28**:393–417. doi:10.1007/s10926-017-9730-1

17 Oke A, Braithwaite P, Antai D. Sickness Absence and Precarious Employment: A Comparative Cross-National Study of Denmark, Finland, Sweden, and Norway. *Int J Occup Environ Med* 2016;**7**:125–47. doi:10/gmx883

18 Matilla-Santander N, González-Marrón A, Martín-Sánchez JC, *et al.* Precarious employment and health-related outcomes in the European Union: a cross-sectional study. *Crit Public Health* 2020;**30**:429–40. doi:10.1080/09581596.2019.1587385

19 Ronnblad T, Gronholm E, Jonsson J, *et al.* Precarious employment and mental health: a systematic review and meta-analysis of longitudinal studies. *Scand J Work Env Health* 2019;**45**:429–43. doi:10.5271/sjweh.3797

20 Jonsson J, Muntaner C, Bodin T, *et al.* Low-quality employment trajectories and risk of common mental disorders, substance use disorders and suicide attempt: a longitudinal study of the Swedish workforce. *Scand J Work Env Health* 2021;**47**:208–16. doi:10/gmprvj

21 Sickness benefit for those with sole proprietorship. http://www.forsakringskassan.se/english/sick/sole-proprietorship/sickness-benefit-for-those-with-sole-proprietorship (accessed 29 Sep 2022).

22 Hutchins DS, Zeber JE, Roberts CS, *et al.* Initial Medication Adherence—Review and Recommendations for Good Practices in Outcomes Research: An ISPOR Medication Adherence and Persistence Special Interest Group Report. *Value Health* 2015;**18**:690–9. doi:10.1016/j.jval.2015.02.015

23 Aznar-Lou I, Iglesias-González M, Gil-Girbau M, *et al.* Impact of initial medication non-adherence to SSRIs on medical visits and sick leaves. *J Affect Disord* 2018;**226**:282–6. doi:10.1016/j.jad.2017.09.057

24 Bodin T, Jonsson J. Swedish Register Operationalization of Precarious Employment. 2020.https://github.com/TBodin/swe-rope (accessed 15 Mar 2021).

For peer review only - http://bmjopen.bmj.com/site/about/guidelines.xhtml

25 Jonsson J, Matilla-Santander N, Kreshpaj B, *et al.* Exploring multidimensional operationalizations of precarious employment in Swedish register data – a typological approach and a summative score approach. *Scand J Work Environ Health* 2021;**47**:117–26. doi:10.5271/sjweh.3928

26 Pollack R, Kreshpaj B, Jonsson J, *et al.* Low-quality employment trajectories and the risk of common mental health disorders among individuals with Swedish and foreign background - a register-based cohort study. *Scand J Work Environ Health* 2022;**48**:351–60. doi:10.5271/sjweh.4029

27 Overview - SSRI antidepressants. nhs.uk. 2021.https://www.nhs.uk/mental-health/talking-therapies-medicine-treatments/medicines-and-psychiatry/ssri-antidepressants/overview/ (accessed 21 Jul 2022).

28 Machado-Vieira R, Baumann J, Wheeler-Castillo C, *et al.* The Timing of Antidepressant Effects: A Comparison of Diverse Pharmacological and Somatic Treatments. *Pharmaceuticals* 2010;**3**:19–41. doi:10.3390/ph3010019

29 Cheng Q, Huang J, Xu L, *et al.* Analysis of Time-Course, Dose-Effect, and Influencing Factors of Antidepressants in the Treatment of Acute Adult Patients With Major Depression. *Int J Neuropsychopharmacol* 2020;**23**:76–87. doi:10.1093/ijnp/pyz062

30 Swedish Social Insurance Agency [Försäkringskassan]. Social Insurance in Figures 2020. 2020.

31 Roelen C a. M, Koopmans PC, Schreuder J a. H, *et al.* The history of registered sickness absence predicts future sickness absence. *Occup Med Oxf Engl* 2011;**61**:96–101. doi:10/ct43k7

32 Ármannsdóttir B, Mårdby A-C, Haukenes I, *et al.* Cumulative incidence of sickness absence and disease burden among the newly sick-listed, a cross-sectional population-based study. *BMC Public Health* 2013;**13**:329. doi:10.1186/1471-2458-13-329

33 Staland-Nyman C, Houkes I, de Rijk A, *et al.* Gender equality in domestic work and sickness absence - a population-based study on women and men in Sweden. *Women Health* 2021;**61**:325–36. doi:10.1080/03630242.2021.1872759

34 Amrhein V, Greenland S, McShane B. Scientists rise up against statistical significance. *Nature* 2019;**567**:305–7. doi:10.1038/d41586-019-00857-9

35 Johansen V, Aronsson G, Marklund S. Positive and negative reasons for sickness presenteeism in Norway and Sweden: a cross-sectional survey. *BMJ Open* 2014;**4**:e004123. doi:10.1136/bmjopen-2013-004123

36 Aronsson G, Gustafsson K. Sickness Presenteeism: Prevalence, Attendance-Pressure Factors, and an Outline of a Model for Research. *J Occup Environ Med* 2005;**47**:958–66.

37 Dewa CS, Lin E. Chronic physical illness, psychiatric disorder and disability in the workplace. *Soc Sci Med* 2000;**51**:41–50. doi:10.1016/S0277-9536(99)00431-1

For peer review only - http://bmjopen.bmj.com/site/about/guidelines.xhtml

38 Inspektionen för socialförsäkringen. Tidsbegränsade anställningar och sjukförsäkringen 2016. 2016.

39 *Socialförsäkringen i siffror 2022*. Stockholm: : Försäkringskassan 2022.

40 Lytsy P, Alexanderson K, Friberg E. Gender differences in treatment with antidepressants during first weeks of a sick-leave spell due to depressive episode. *Eur J Public Health* 2020;**30**:299–304. doi:10.1093/eurpub/ckz172

41 Marklund S, Gustafsson K, Bergström G, *et al.* Reasons for presenteeism in different occupational branches in Sweden: a population based cross-sectional study. *Int Arch Occup Environ Health* 2021;**94**:1385–95. doi:10.1007/s00420-021-01701-2

42 Piha K, Laaksonen M, Martikainen P, *et al.* Interrelationships between education, occupational class, income and sickness absence. *Eur J Public Health* 2010;**20**:276–80. doi:10.1093/eurpub/ckp162

43 Hansen CD, Andersen JH. Going ill to work – What personal circumstances, attitudes and work-related factors are associated with sickness presenteeism? *Soc Sci Med* 2008;**67**:956–64. doi:10.1016/j.socscimed.2008.05.022

44 Steidelmüller C, Meyer S-C, Müller G. Home-Based Telework and Presenteeism Across Europe. *J Occup Environ Med* 2020;**62**:998–1005. doi:10.1097/JOM.0000000000001992

45 Roshanaei-Moghaddam B, Pauly MC, Atkins DC, *et al.* Relative effects of CBT and pharmacotherapy in depression versus anxiety: is medication somewhat better for depression, and CBT somewhat better for anxiety? *Depress Anxiety* 2011;**28**:560–7. doi:10.1002/da.20829

46 Grossman P, Niemann L, Schmidt S, *et al.* Mindfulness-based stress reduction and health benefits: A meta-analysis. *J Psychosom Res* 2004;**57**:35–43. doi:10.1016/S0022-3999(03)00573-7

47 Taloyan M, Aronsson G, Leineweber C, *et al.* Sickness Presenteeism Predicts Suboptimal Self-Rated Health and Sickness Absence: A Nationally Representative Study of the Swedish Working Population. *PLOS ONE* 2012;**7**:e44721. doi:10.1371/journal.pone.0044721

48 The serotonin theory of depression: a systematic umbrella review of the evidence | Molecular Psychiatry. https://www.nature.com/articles/s41380-022-01661-0 (accessed 4 Oct 2022).

49 Ludvigsson JF, Svedberg P, Olén O, *et al.* The longitudinal integrated database for health insurance and labour market studies (LISA) and its use in medical research. *Eur J Epidemiol* 2019;**34**:423–37. doi:10.1007/s10654-019-00511-8

For peer review only - http://bmjopen.bmj.com/site/about/guidelines.xhtml

Figures legend:

Figure 1. Flow chart of the total study population.

This is a flow chart of the exclusion criteria applied (there is no legend)

Figure 2. Directed Acyclic Graph (DAG) of the relation between employment quality typologies (EQT) and sickness absence benefits (SABs) due to common mental disorders (CMDs).

For peer review only

For peer review only - http://bmjopen.bmj.com/site/about/guidelines.xhtml

Figure 1. Flow chart of the total study population.

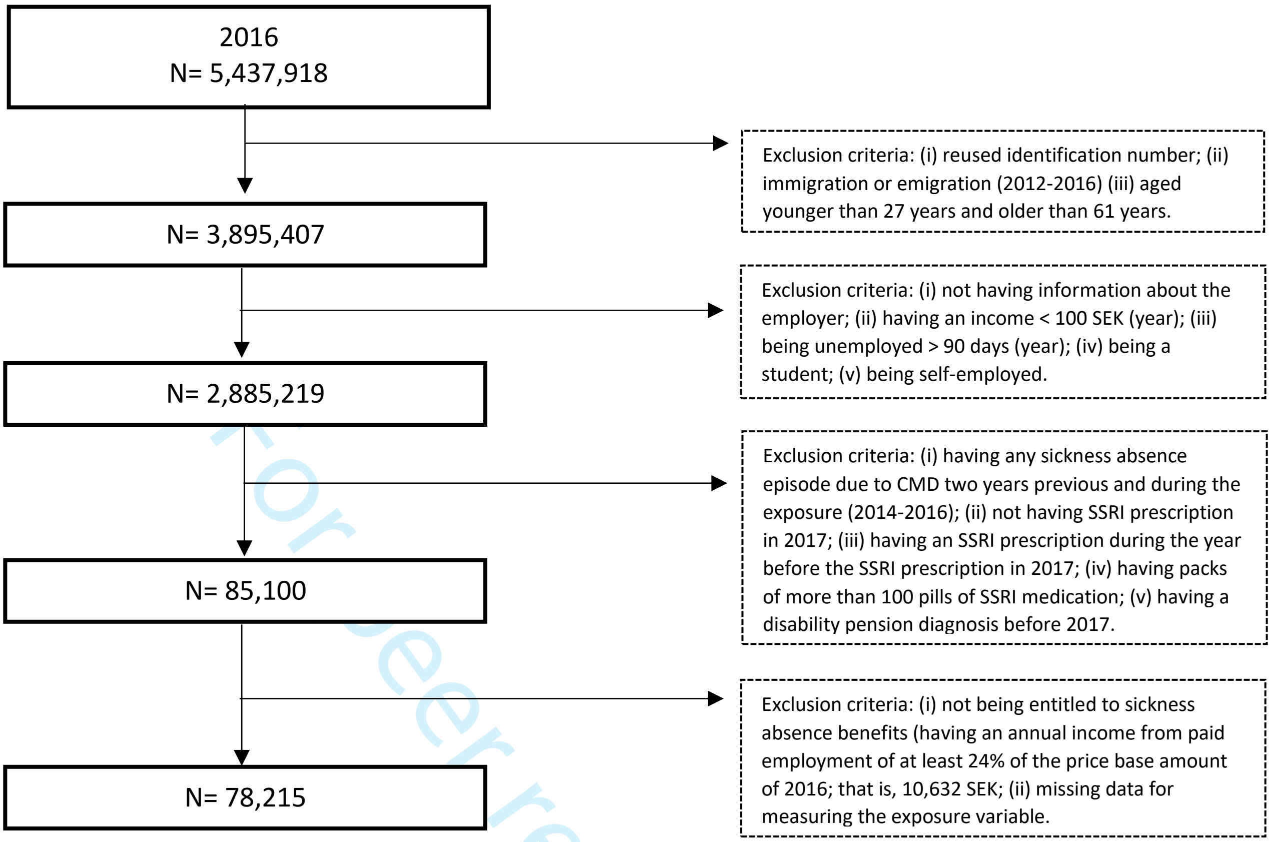

For peer review only

For peer review only - http://bmjopen.bmj.com/site/about/guidelines.xhtml

Figure 2. Directed Acyclic Graph (DAG) of the relation between employment quality typologies (EQT) and sickness absence benefits (SABs) due to common mental disorders (CMDs).

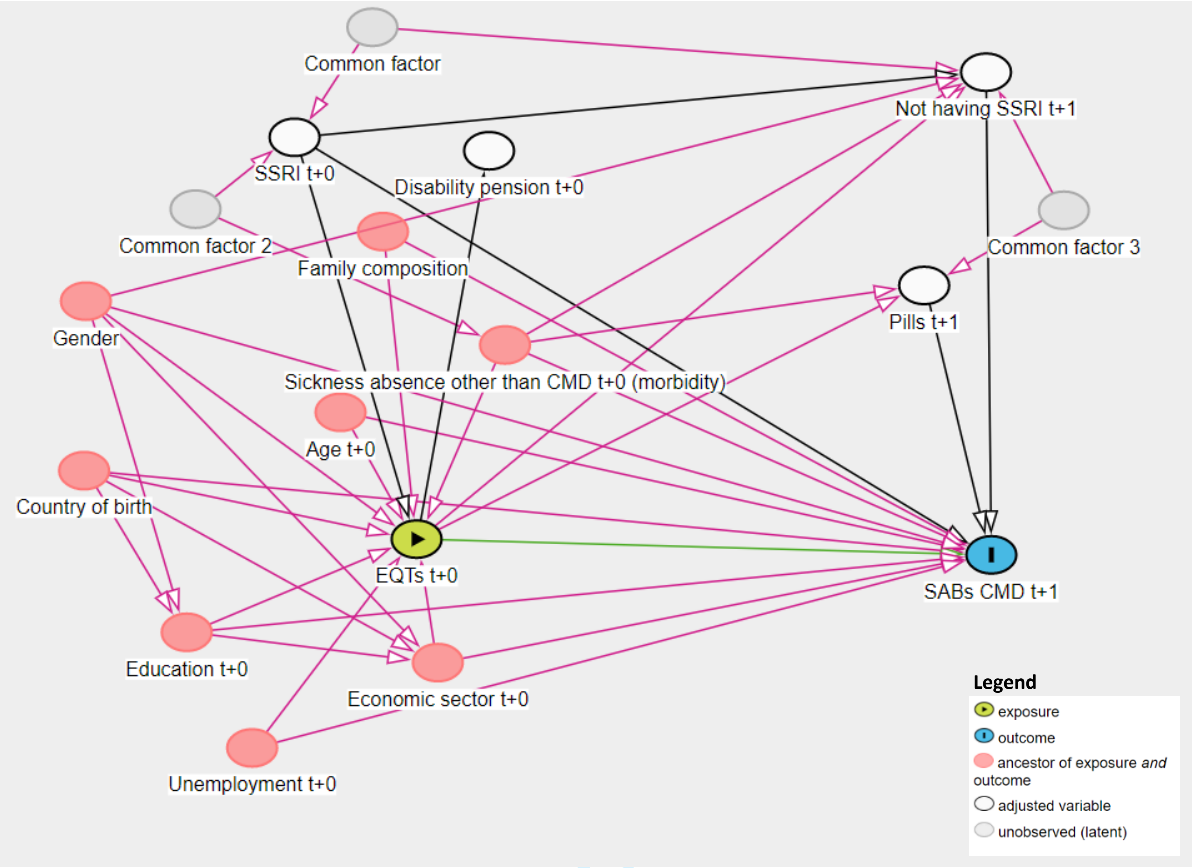

Minimal sufficient adjustment sets for estimating the direct effect of EQTs t+0 (baseline, 2016) on SABs due to CMD t+1 is age, gender, education, country of birth, family composition, economic sector, unemployment, and sickness absence due to diagnoses other than CMD, having Selective Serotonin Reuptake Inhibitors (SSRI) prescription during the year before the SSRI prescription in 2017, having disability pension before 2017, having packs of more than 100 pills in 2017 and not having Selective Serotonin Reuptake Inhibitors SSRI prescriptions close to the SABs in 2017.

For peer review only - http://bmjopen.bmj.com/site/about/guidelines.xhtml

Supplementary Table 1. Sociodemographic and employment-related characteristics at baseline (2016) of the Swedish working population and the study population with common mental disorders.

	Population*	Study population**
	N (%)	N (%)
**Age**		
27-40 years	1,044,971 (37.4)	30,675 (39.2)
41-51 years	976,697 (34.9)	28,541 (36.5)
52-61 years	774,838 (27.7)	18,999 (24.3)
**Gender**		
Male	1,412,310 (50.5)	25,894 (33.1)
Female	1,384,196 (49.5)	52,321 (66.9)
**Education level**		
Elementary/high school education	1,518,999 (54.4)	41,072 (52.5)
Higher education <3 years	429,648 (15.4)	11,456 (14.7)
Higher education ≥3 years	845,355 (30.3)	25,651 (32.8)
**Country of birth**		
Sweden	2,459,096 (88.0)	69,736 (89.2)
Not Sweden	335,518 (12.0)	8,438 (10.8)
**Family composition**		
Couple with children	1,400,506 (50.1)	37,471 (47.9)
Couple without children	352,101 (12.6)	8,421 (10.8)
Single with children	260,276 (9.3)	10,243 (13.1)
Single without children	783,622 (28.0)	22,080 (28.2)
**Unemployment**		
0 days unemployed	2,718,662 (97.2)	76,090 (97.3)
From 1 to 90 days unemployed	77,844 (2.8)	2,125 (2.7)
**Economic sector**		
Private	1,741,419 (62.3)	41,653 (53.3)
Public	1,055,087 (37.7)	36,562 (46.7)
TOTAL	2,796,506 (100.0)	78,215 (100.0)

*The entire study population meeting the inclusion and exclusion criteria (except having Selective Serotonin Reuptake Inhibitors prescriptions in 2017); **The final study population meeting the whole inclusion and exclusion criteria. Missing values in educational level (population: 2,504, 0.09%; study population: 41, 0.05%), country of birth (population: 1892, 0.07%; study population: 41, 0.05%) and family composition (population: 1, <0.01%).

For peer review only - http://bmjopen.bmj.com/site/about/guidelines.xhtml

Supplementary Table 2. Incidence of a first sickness absence episode due to common mental disorders in a cohort of 78,215 individuals with common mental disorders in 2017, aged 27-61, entitled to sickness absence benefits in 2016, by sociodemographic and employment characteristics at baseline (2016)

	**Sickness absence benefits (7.48%)**
	N (%)
**Age**	
27-40 years	2,808 (9.2)
41-51 years	1,901 (6.7)
52-61 years	1,139 (6.0)
**Gender**	
Male	2,136 (8.2)
Female	3,712 (7.1)
**Education level**	
Elementary/high school education	3,502 (8.5)
Higher education <3 years	793 (6.9)
Higher education ≥3 years	1,547 (6.0)
**Country of birth**	
Sweden	5,058 (7.3)
Not Sweden	783 (9.3)
**Family composition**	
Couple with children	2,678 (7.1)
Couple with no children	519 (6.2)
Single with children	808 (7.9)
Single	1,843 (8.3)
**Unemployment**	
0 days unemployed	5,697 (7.5)
From 1 to 90 days unemployed	151 (7.1)
**Economic sector**	
Private	3218 (7.7)
Public	2630 (7.2)
**Employment quality typology**	
Standard Employment Relationship/HI	593 (5.2)
Standard Employment Relationship	2,268 (7.6)
Sub-standard Employment Relationship	2,667 (8.1)
Precarious Employment Relationship	320 (8.0)
**Contractual relationship insecurity**	
Directly employed by an employer	5,788 (7.5)
Employed by an agency	60 (8.2)
**Contractual temporariness**	
Stable employment	4,351 (7.4)
Unstable employment	1,497 (7.8)
**Multiple jobs/economic sectors**	
1-2 jobs	5,436 (7.4)
3 or more jobs	279 (7.8)
3 or more jobs in 3 or more sectors	133 (8.2)
**Income level***	
<60% of the median	395 (7.6)
60-79% of the median	1,415 (8.9)
80-119% of the median	3,113 (7.7)
120-200% of the median	831 (5.7)
>200% of the median	94 (4.0)
**Unionization level**	
<70 %	124 (7.0)
70-90%	315 (6.8)
>90%	5,409 (7.5)

Missing values in the educational level (36, 0.05%) and country of birth (41, 0.05%). Note: Persons entitled to sickness absence benefits in 2016 are those having an annual income from paid employment of at least 24% of the price base amount of 2016; that is, 10,632 SEK. *Income level categories based on the median of the Swedish working population (375,300 SEK) after applying several inclusion and exclusion criteria (see methods section). Income category boundaries: <60% of the median (225,180 SEK), 60-79% of the median (225,180-296,487 SEK), 80-119% of the median (300,240-446,607 SEK), 120-199% of the median (450,360-746,847 SEK), and ≥200% of the median (750,600 SEK).

For peer review only

For peer review only - http://bmjopen.bmj.com/site/about/guidelines.xhtml

Supplementary Table 3. Incidence of a first sickness absence episode due to common mental disorders in 2017 in women and men in a cohort of 85,164 individuals with common mental disorders, aged 27-61, entitled to sickness absence benefits in 2016, by sociodemographic and employment characteristics at baseline (2016).

	Sickness absence benefits	
	Women (7.1%)	Men (8.2%)
	N (%)	N (%)
**Age**		
27-40 years	1782 (8.7)	1026 (10.2)
41-51 years	1204 (6.3)	697 (7.4)
52-61 years	726 (5.8)	413 (6.4)
**Education level**		
Elementary/high school education	2016 (8.0)	1486 (9.3)
Higher education <3 years	484 (6.3)	309 (8.2)
Higher education ≥3 years	1208 (6.2)	339 (5.5)
**Country of birth**		
Sweden	3195 (6.9)	1863 (8.0)
Not Sweden	512 (8.8)	271 (10.4)
**Family composition**		
Couple with children	1735 (6.8)	943 (7.8)
Couple without children	357 (6.2)	162 (6.2)
Single with children	647 (7.6)	161 (9.2)
Single without children	973 (7.7)	870 (9.2)
**Unemployment**		
0 days unemployed	3632 (7.1)	2065 (8.2)
From 1 to 90 days unemployed	80 (6.0)	71 (8.9)
**Economic sector**		
Private	1,594 (7.0)	1,624 (8.5)
Public	2,118 (7.1)	512 (7.4)
**Employment quality typology**		
Standard Employment Relationship/HI	247 (4.5)	346 (5.8)
Standard Employment Relationship	1375 (7.0)	893 (8.8)
Sub-standard Employment Relationship	1892 (7.7)	775 (9.1)
Precarious Employment Relationship	198 (7.1)	122 (10.1)
**Contractual relationship insecurity**		
Directly employed by an employer	3683 (7.1)	2105 (8.2)
Employed by an agency	29 (6.5)	31 (10.9)
**Contractual temporariness**		
Stable employment	2792 (7.0)	1559 (8.0)
Unstable employment	920 (7.2)	577 (9.0)
**Multiple jobs/economic sectors**		
1-2 jobs	3463 (7.1)	1973 (8.2)
3 or more jobs	175 (7.0)	104 (9.6)
3 or more jobs in 3 or more sectors	74 (7.3)	59 (9.6)
**Income level**		
<60% of the median	319 (7.3)	76 (8.9)
60-79% of the median	1111 (8.4)	304 (10.9)
80-119% of the median	1876 (7.1)	1237 (8.9)
120-200% of the median	378 (5.1)	453 (6.4)
>200% of the median	28 (2.9)	66 (4.9)
**Unionization level**		
<70 %	54 (5.8)	70 (8.2)
70-90%	161 (6.1)	154 (7.7)
>90%	3497 (7.2)	1912 (8.3)

Missing values in education level (women: 19, 0.04%; men: 17, 0.07%) and country of birth (women: 19, 0.04%; men: 22, 0.08%). Note: Persons entitled to sickness absence benefits in 2016 are those having an annual income from paid employment of at least 24% of the price base amount of 2016; that is, 10,632 SEK. *Income level categories based on the median of the Swedish working population (375,300 SEK) after applying several inclusion and exclusion criteria (see methods section). Income category boundaries: <60% of the median (225,180 SEK), 60-79% of the median (225,180-296,487 SEK), 80-119% of the median (300,240-446,607 SEK), 120-199% of the median (450,360-746,847 SEK), and ≥200% of the median (750,600 SEK).

For peer review only

Supplementary Table 4. Incidence of a first sickness absence episode due to common mental disorders in a cohort of 85,164 individuals with common mental disorders in 2017, aged 27-61, entitled to sickness absence benefits in 2016, by employment quality typology (Standard Employment Relationship, SER; Standard Employment Relationship with high income, SER/HI; Sub-standard Employment Relationship, SSER; Precarious Employment Relationship, PER; baseline, 2016).

	Sickness absence benefits			
	**SER/HI (5.2%)**	**SER (7.6%)**	**SSER (8.1%)**	**PER (8%)**
	N (%)	N (%)	N (%)	N (%)
**Age**				
27-40 years	180 (6.3)	938 (9.6)	1481 (9.5)	209 (8.8)
41-51 years	253 (4.8)	794 (6.9)	773 (7.1)	81 (7.9)
52-61 years	160 (4.8)	536 (6.3)	413 (6.3)	30 (5.1)
**Gender**				
Male	346 (5.8)	893 (8.8)	775 (9.1)	122 (10.1)
Female	247 (4.5)	1375 (7.0)	1892 (7.7)	198 (7.1)
**Education level**				
Elementary/high school education	209 (6.2)	1340 (8.5)	1731 (9.0)	222 (8.8)
Higher education <3 years	116 (5.8)	321 (7.3)	311 (7.0)	45 (7.1)
Higher education ≥3 years	268 (4.4)	606 (6.4)	620 (6.7)	53 (6.4)
**Country of birth**				
Sweden	527 (5.0)	1962 (7.4)	2282 (7.8)	287 (8.1)
Not Sweden	66 (6.4)	305 (9.6)	379 (10.1)	33 (7.1)
**Family composition**				
Couple with children	288 (4.8)	971 (7.3)	1286 (7.9)	133 (7.5)
Couple without children	65 (4.8)	241 (6.6)	202 (6.4)	11 (3.8)
Single with children	59 (4.7)	304 (7.8)	396 (8.7)	49 (8.8)
Single without children	181 (6.4)	752 (8.4)	783 (8.7)	127 (9.3)
**Unemployment**				
0 days unemployed	590 (5.2)	2253 (7.6)	2589 (8.2)	265 (7.8)
From 1 to 90 days unemployed	3 (5.6)	15 (6.3)	78 (6.2)	55 (9.6)
**Economic sector**				
Private	384 (5.5)	1,186 (8.5)	1,383 (7.9)	265 (8.3)
Public	209 (4.7)	1,082 (6.8)	1,284 (8.3)	55 (6.8)
**Contractual relationship insecurity**				
Directly employed by an employer	593 (5.2)	2263 (7.6)	2636 (8.1)	296 (7.9)
Employed by an agency	0	5 (13.2)	31 (6.7)	24 (10.9)
**Contractual temporariness**				
Stable employment	593 (5.2)	2246 (7.7)	1503 (8.3)	9 (3.5)
Unstable employment	0	22 (4.7)	1164 (7.8)	311 (8.3)
**Multiple jobs/economic sectors**				
1-2 jobs	579 (5.2)	2220 (7.6)	2479 (8.1)	158 (7.3)
3 or more jobs	11 (5.0)	36 (8.2)	155 (8.1)	77 (7.7)
3 or more jobs in 3 or more sectors	3 (2.5)	12 (6.3)	33 (6.7)	85 (10.3)
**Income level**				
<60% of the median	0	0	270 (8.1)	125 (6.6)
60-79% of the median	0	0	1313 (8.9)	102 (8.8)
80-119% of the median	0	2203 (7.7)	822 (7.7)	88 (10.0)
120-200% of the median	536 (5.4)	42 (7.1)	249 (6.3)	4 (6.5)
>200% of the median	57 (3.5)	23 (4.5)	13 (7.1)	1 (100.0)
**Unionization level**				
<70%	0	1 (5.0)	58 (6.8)	65 (7.2)
70-90%	2 (3.6)	26 (6.3)	223 (6.8)	64 (7.4)
>90%	591 (5.2)	2241 (7.6)	2386 (8.3)	191 (8.6)

Missing values in education level (SER/HI: 7, 0.06%; SER: 10, 0.03%; SSER: 17, 0.05%; PE: 2, 0.05%) and country of birth (SER/HI: 3, 0.03%; SER: 18, 0.06%; SSER: 20, 0.06%). Note: Persons entitled to sickness absence benefits in 2016 are those having an annual income from paid employment of at least 24% of the price base amount of 2016; that is, 10,632 SEK. *Income level categories based on the median of the Swedish working population (375,300 SEK) after applying several inclusion and exclusion criteria (see methods section). Income category boundaries: <60% of the median (225,180 SEK), 60-79% of the median (225,180-296,487 SEK), 80-119% of the median (300,240-446,607 SEK), 120-199% of the median (450,360-746,847 SEK), and ≥200% of the median (750,600 SEK).

For peer review only

Supplementary Table 5. Association between employment quality dimensions (2016, baseline) and sickness absence benefits due to CMD in 2017 (N=78,215) among people entitled to sickness absence benefits in 2016.

					Women (N=52,321)				Men (N=25,894)			
	Cases N(%)	OR (95% CI)	aOR (95% CI) [1]	aOR (95% CI) [2]	Cases N(%)	OR (95% CI)	aOR (95% CI) [1]	aOR (95% CI) [2]	Cases N(%)	OR (95% CI)	aOR (95% CI) [1]	aOR (95% CI) [2]
**Contractual relationship insecurity**												
Directly employed by an employer	5,788 (7.5)	1	1	1	3,683 (7.1)	1	1	1	2,105 (8.2)	1	1	1
Employed by an agency	60 (8.2)	1.10 (0.85-1.44)	1.05 (0.80-1.37)	0.96 (0.73-1.25)	29 (6.5)	0.91 (0.62-1.32)	0.89 (0.61-1.31)	0.85 (0.58-1.24)	31 (10.9)	1.36 (0.94-1.98)	1.19 (0.81-1.73)	1.08 (0.74-1.58)
**Contractual temporariness**												
Stable employment	4,351 (7.4)	1	1	1	2,792 (7.1)	1	1	1	1,559 (8.0)	1	1	1
Unstable employment	1,497 (7.8)	1.07 (1.01-1.14)	1.08 (1.02-1.15)	1.02 (0.95-1.09)	920 (7.2)	1.03 (0.95-1.11)	1.06 (0.98-1.15)	1.01 (0.92-1.10)	577 (9.0)	1.14 (1.03-1.26)	1.12 (1.01-1.24)	1.04 (0.93-1.16)
**Multiple jobs/economic sectors**												
1-2 jobs	5,436 (7.4)	1	1	1	3,463 (7.1)	1	1	1	1,973 (8.2)	1	1	1
3 or more jobs	279 (7.8)	1.05 (0.93-1.19)	1.06 (0.93-1.20)	1.05 (0.93-1.20)	175 (7.0)	0.99 (0.85-1.16)	1.02 (0.87-1.20)	1.00 (0.85-1.18)	104 (9.6)	1.19 (0.97-1.47)	1.17 (0.95-1.45)	1.18 (0.95-1.46)
3 or more jobs in 3 or more sectors	133 (8.2)	1.11 (0.93-1.33)	1.11 (0.93-1.34)	1.09 (0.91-1.31)	74 (7.3)	1.04 (0.82-1.32)	1.07 (0.84-1.36)	1.04 (0.81-1.32)	59 (9.6)	1.19 (0.91-1.56)	1.16 (0.88-1.53)	1.19 (0.90-1.57)
**Income level**												
80-119% of the median	3,113 (7.7)	1	1	1	1,876 (7.1)	1	1	1	1,237 (8.9)	1	1	1
<60% of the median	395 (7.6)	0.98 (0.88-1.09)	0.98 (0.88-1.10)	0.92 (0.82-1.03)	319 (7.3)	1.03 (0.91-1.17)	1.05 (0.93-1.19)	0.91 (0.81-1.04)	76 (8.9)	1.00 (0.78-1.27)	0.99 (0.77-1.26)	0.94 (0.73-1.20)
60-79% of the median	1,415 (8.9)	1.16 (1.09-1.24)	1.17 (1.09-1.24)	1.10 (1.03-1.18)	1,111 (8.4)	1.20 (1.11-1.30)	1.21 (1.12-1.31)	1.08 (1.00-1.17)	304 (10.9)	1.24 (1.09-1.42)	1.25 (1.10-1.43)	1.16 (1.01-1.33)
120-200% of the median	831 (5.8)	0.73 (0.67-0.79)	0.72 (0.67-0.78)	0.79 (0.72-0.85)	378 (5.1)	0.70 (0.63-0.79)	0.70 (0.63-0.79)	0.79 (0.70-0.88)	453 (6.4)	0.70 (0.62-0.78)	0.69 (0.62-0.78)	0.80 (0.71-0.89)
>200% of the median	94 (4.1)	0.50 (0.41-0.62)	0.50 (0.40-0.62)	0.61 (0.49-0.75)	28 (2.9)	0.39 (0.26-0.57)	0.39 (0.26-0.56)	0.48 (0.33-0.71)	66 (4.9)	0.52 (0.41-0.68)	0.51 (0.40-0.66)	0.71 (0.54-0.92)
**Unionization level**												
>90%	5,409 (7.5)	1	1	1	3,497 (7.2)	1	1	1	1,912 (8.3)	1	1	1
70-90%	315 (6.8)	0.90 (0.80-1.01)	0.86 (0.77-0.97)	0.82 (0.73-0.92)	161 (6.1)	0.84 (0.72-0.99)	0.82 (0.70-0.97)	0.83 (0.70-0.98)	154 (7.7)	0.92 (0.78-1.10)	0.84 (0.71-1.00)	0.80 (0.67-0.96)
<70%	124 (7.0)	0.92 (0.77-1.11)	0.87 (0.72-1.05)	0.82 (0.68-0.99)	54 (5.8)	0.80 (0.61-1.06)	0.76 (0.58-1.01)	0.77 (0.58-1.03)	70 (8.2)	0.99 (0.77-1.27)	0.87 (0.67-1.12)	0.84 (0.65-1.09)

OR: unadjusted Odds Ratio; [1] aOR: Odds Ratio adjusted mutually by the other employment quality dimensions in the baseline (2016); [2] aOR: Odds Ratio adjusted mutually by the other employment quality dimensions and age, gender, education, country of birth, family composition, economic sector, unemployment, and SA due to diagnoses other than CMD in the baseline (2016); Note: Persons entitled to sickness absence benefits are those having an annual income from paid employment of at least 24% of the price base amount of 2016; that is, 10,632 SEK. *Income level categories based on the median of the Swedish working population after applying the inclusion and some of the main exclusion criteria (see methods section). *Income level categories based on the median of the Swedish working population (375,300 SEK) after applying several inclusion and exclusion criteria (see methods section). Income category boundaries: <60% of the median (225,180 SEK), 60-79% of the median (225,180-296,487 SEK), 80-119% of the median (300,240-446,607 SEK), 120-199% of the median (450,360-746,847 SEK), and ≥200% of the median (750,600 SEK). Area of residence and occupation barely changed the estimates; therefore, they were not included in the models as confounders.

For peer review only

STROBE Statement—checklist of items that should be included in reports of observational studies

	Item No.	Recommendation	Page No.	Relevant text from manuscript
**Title and abstract**	1	(*a*) Indicate the study's design with a commonly used term in the title or the abstract	2	
		(*b*) Provide in the abstract an informative and balanced summary of what was done and what was found	2	
**Introduction**				
Background/rationale	2	Explain the scientific background and rationale for the investigation being reported	4	
Objectives	3	State specific objectives, including any prespecified hypotheses	4	
**Methods**				
Study design	4	Present key elements of study design early in the paper	5	
Setting	5	Describe the setting, locations, and relevant dates, including periods of recruitment, exposure, follow-up, and data collection	5	
Participants	6	(*a*) *Cohort study*—Give the eligibility criteria, and the sources and methods of selection of participants. Describe methods of follow-up<br>*Case-control study*—Give the eligibility criteria, and the sources and methods of case ascertainment and control selection. Give the rationale for the choice of cases and controls<br>*Cross-sectional study*—Give the eligibility criteria, and the sources and methods of selection of participants	5	
		(*b*) *Cohort study*—For matched studies, give matching criteria and number of exposed and unexposed<br>*Case-control study*—For matched studies, give matching criteria and the number of controls per case		
Variables	7	Clearly define all outcomes, exposures, predictors, potential confounders, and effect modifiers. Give diagnostic criteria, if applicable	5,6	
Data sources/ measurement	8*	For each variable of interest, give sources of data and details of methods of assessment (measurement). Describe comparability of assessment methods if there is more than one group	5-6	
Bias	9	Describe any efforts to address potential sources of bias	2 (fig. file)	Figure 2
Study size	10	Explain how the study size was arrived at	1 (fig. file)	Figure 1
Continued on next	11	Explain how quantitative variables were handled in the analyses. If applicable, describe which	6	

For peer review only

page Quantitative variables		groupings were chosen and why		
Statistical methods	12	(*a*) Describe all statistical methods, including those used to control for confounding	6	
		(*b*) Describe any methods used to examine subgroups and interactions	6	
		(*c*) Explain how missing data were addressed	N/A	
		(*d*) *Cohort study*—If applicable, explain how loss to follow-up was addressed<br>*Case-control study*—If applicable, explain how matching of cases and controls was addressed<br>*Cross-sectional study*—If applicable, describe analytical methods taking account of sampling strategy	N/A	
		(*e*) Describe any sensitivity analyses	6	
**Results**				
Participants	13*	(a) Report numbers of individuals at each stage of study—eg numbers potentially eligible, examined for eligibility, confirmed eligible, included in the study, completing follow-up, and analysed	1 (fig and supp files)	Figure 1 and Suppl. Table 1
		(b) Give reasons for non-participation at each stage	1 (fig file)	Figure 1
		(c) Consider use of a flow diagram	1 (fig file)	Figure 1
Descriptive data	14*	(a) Give characteristics of study participants (eg demographic, clinical, social) and information on exposures and potential confounders	8	Table 1
		(b) Indicate number of participants with missing data for each variable of interest	8 and 1-4 (supp file)	Footnote Table 1 and Suppl. Tables 1-4 (supp file)
		(c) *Cohort study*—Summarise follow-up time (eg, average and total amount)	N/A	
Outcome data	15*	*Cohort study*—Report numbers of outcome events or summary measures over time	9,11	Tables 2 and 3
		Case-control study—Report numbers in each exposure category, or summary measures of exposure		
		Cross-sectional study—Report numbers of outcome events or summary measures		
Main results	16	(*a*) Give unadjusted estimates and, if applicable, confounder-adjusted estimates and their precision (eg, 95% confidence interval). Make clear which confounders were adjusted for and why they were included	9,11	Tables 2 and 3
		(*b*) Report category boundaries when continuous variables were categorized	8,11 and 2-4 (supp file)	Tables 1,3 and Suppl. Tables 2-4 (supp file)
		(*c*) If relevant, consider translating estimates of relative risk into absolute risk for a meaningful time period	N/A	

For peer review only - http://bmjopen.bmj.com/site/about/guidelines.xhtml
For peer review only

Continued on next page

Other analyses	17	Report other analyses done—eg analyses of subgroups and interactions, and sensitivity analyses	N/A	
**Discussion**				
Key results	18	Summarise key results with reference to study objectives	12	First paragraph of the discussion
Limitations	19	Discuss limitations of the study, taking into account sources of potential bias or imprecision. Discuss both direction and magnitude of any potential bias	13	
Interpretation	20	Give a cautious overall interpretation of results considering objectives, limitations, multiplicity of analyses, results from similar studies, and other relevant evidence	12-13	
Generalisability	21	Discuss the generalisability (external validity) of the study results	14	
**Other information**				
Funding	22	Give the source of funding and the role of the funders for the present study and, if applicable, for the original study on which the present article is based	14	

*Give information separately for cases and controls in case-control studies and, if applicable, for exposed and unexposed groups in cohort and cross-sectional studies.

Note: An Explanation and Elaboration article discusses each checklist item and gives methodological background and published examples of transparent reporting. The STROBE checklist is best used in conjunction with this article (freely available on the Web sites of PLoS Medicine at http://www.plosmedicine.org/, Annals of Internal Medicine at http://www.annals.org/, and Epidemiology at http://www.epidem.com/). Information on the STROBE Initiative is available at www.strobe-statement.org.

For peer review only

For peer review only - http://bmjopen.bmj.com/site/about/guidelines.xhtml
